# A single-cell and spatial genomics atlas of human skin fibroblasts reveals shared disease-related fibroblast subtypes across tissues

**DOI:** 10.1038/s41590-025-02267-8

**Published:** 2025-09-24

**Authors:** Lloyd Steele, Bayanne Olabi, Kenny Roberts, Pavel V. Mazin, Simon Koplev, Catherine Tudor, Benjamin Rumney, Chloe Admane, Treasa Jiang, Donovan Correa-Gallegos, Keerthi Priya Chakala, Aljes Binkevich, Nusayhah Hudaa Gopee, Alexander Predeus, Martin Prete, Elena Winheim, Karl Annusver, Agnes Forsthuber, Luc Francis, Sophie Frech, Clarisse Ganier, Thomas Layton, Yingzi Liu, Hao Yuan, Johann E. Gudjonsson, Beate M. Lichtenberger, Satveer Mahil, Jagdeep Nanchahal, Edel A. O’Toole, Maksim V. Plikus, Yuval Rinkevich, Emanuel Rognoni, Catherine H. Smith, Sarah A. Teichmann, Maria Kasper, April R. Foster, Mohammad Lotfollahi, Muzlifah Haniffa

**Affiliations:** 1 Wellcome Sanger Institute, Wellcome Genome Campus, Cambridge, UK; 2 Trinity College, University of Cambridge, Cambridge, UK; 3 Department of Dermatology and NIHR Newcastle Biomedical Research Centre, Newcastle, UK; 4 Cambridge Stem Cell Institute, University of Cambridge, Cambridge, UK; 5 St John’s Institute of Dermatology, King’s College London and Guy’s & St Thomas’ NHS Foundation Trust, London, UK; 6 Institute for Stroke and Dementia Research (ISD), University Hospital (Klinikum LMU), Munich, Germany; 7 Department of Cell and Molecular Biology, Karolinska Institutet, Stockholm, Sweden; 8 Department of Dermatology, Medical University of Vienna, Vienna, Austria; 9 Institut Pasteur, Immunology department, Meta-organism Unit, Paris, France; 10 Kennedy Institute of Rheumatology, Nuffield Department of Orthopaedics, Rheumatology, and Musculoskeletal Sciences, University of Oxford, Oxford, UK; 11 Department of Developmental and Cell Biology, Charlie Dunlop School of Biological Sciences, University of California, Irvine, CA USA; 12 Sue & Bill Gross Stem Cell Research Center, University of California, Irvine, CA USA; 13 Department of Dermatology, University of Michigan Medical School, Ann Arbor, MI USA; 14 Department of Internal Medicine, Division of Rheumatology, University of Michigan Medical School, Ann Arbor, MI USA; 15 Centre for Cell Biology and Cutaneous Research, The Faculty of Medicine and Dentistry, Blizard Institute, Queen Mary University of London, London, UK; 16 Chinese Institutes for Medical Research, Institute of Regenerative Biology and Medicine, Beijing, China; 17 Capital Medical University, Beijing, China; 18 Department of Medicine, University of Cambridge, Cambridge, UK; 19 CIFAR Macmillan Multi-scale Human Program, CIFAR, Toronto, Ontario Canada

**Keywords:** Immunology, Immunological disorders, Cancer

## Abstract

Fibroblasts sculpt the architecture and cellular microenvironments of various tissues. Here we constructed a spatially resolved atlas of human skin fibroblasts from healthy skin and 23 skin diseases, with comparison to 14 cross-tissue diseases. We define six major skin fibroblast subtypes in health and three that are disease-specific. We characterize two fibroblast subtypes further as they are conserved across tissues and are immune-related. The first, F3: fibroblastic reticular cell-like fibroblast (*CCL19*^+^*CD74*^+^*HLA-DRA*^+^), is a fibroblastic reticular cell-like subtype that is predicted to maintain the superficial perivascular immune niche. The second, F6: inflammatory myofibroblasts (*IL11*^+^*MMP1*^+^*CXCL8*^+^*IL7R*^+^), characterizes early human skin wounds, inflammatory diseases with scarring risk and cancer. F6: inflammatory myofibroblasts were predicted to recruit neutrophils, monocytes and B cells across multiple human tissues. Our study provides a harmonized nomenclature for skin fibroblasts in health and disease, contextualized with cross-tissue findings and clinical skin disease profiles.

## Abstract (web summary)

Steele et al. use single-cell RNA sequencing and spatial transcriptomics to provide an atlas of adult human skin fibroblasts in healthy and diseased human skin.

## Main

Fibroblasts are crucial cells for shaping tissue architecture and immune cell niches^1–3^. Studying the heterogeneity of fibroblast subtypes has been challenging due to the scarcity of unique surface markers and their tendency to adopt activated phenotypes during in vitro culture^1,2^. Single-cell RNA sequencing (scRNA-seq) and spatial transcriptomics technologies have overcome these challenges, enabling the dissection of fibroblast heterogeneity in human tissues^4–9^.

While recent studies have described fibroblast states in human skin, they have not spatially resolved their tissue microanatomical location. Very few, if any, have interrogated fibroblasts in diverse disease conditions in the skin and across human tissues^10–18^. Consequently, the fibroblast composition and function in human skin; how it changes across a range of diseases (inflammatory, cancer and fibrosis/scarring); and how these populations relate to other human tissues is still unclear.

In this study, we integrated published large-scale scRNA-seq datasets of healthy human skin and 23 skin diseases and generated spatial transcriptomics data from two different modalities to construct a high-resolution spatially resolved atlas of more than 350,000 adult human skin fibroblasts. We provide a consensus annotation of skin fibroblasts based on gene expression profiles and spatial locations, and contextualize these findings with fibroblast data from other healthy and diseased human tissues. Our scRNA-seq and spatial datasets resources are freely available for download and interactive data exploration at https://cellatlas.io/studies/skin-fibroblast.

## Results

### Human skin fibroblast subtypes are located in distinct tissue microenvironments

**Fig. 1:**
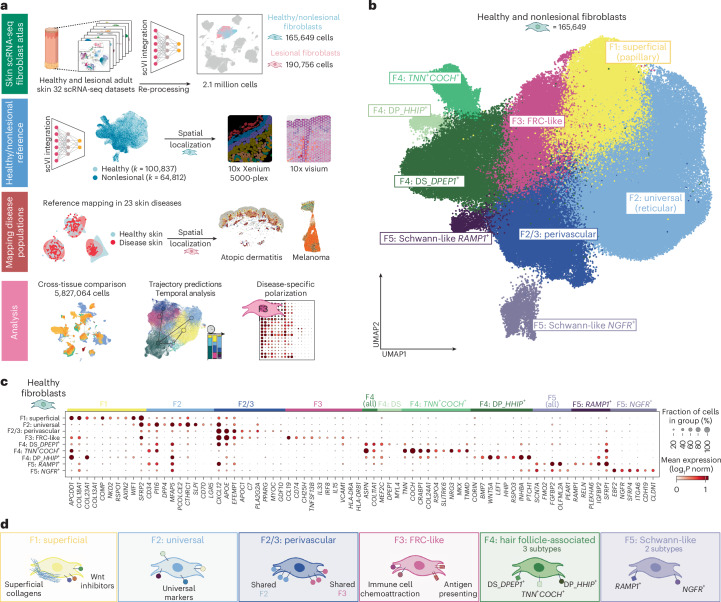
Identification of fibroblast subtypes in healthy skin. **a**, Overview of study methodology, including skin atlas integration to delineate fibroblasts, construction of a healthy/nonlesional reference, mapping of 23 diseases to the healthy reference atlas and downstream analysis for cross-tissue comparison. **b**, Uniform Manifold Approximation and Projection (UMAP) of healthy and nonlesional skin fibroblasts colored by fibroblast subtype. DS, dermal sheath; DP, dermal papilla. **c**, Dotplot of marker gene expression for healthy fibroblasts. ‘All’ indicates a marker for a general population, but which contains subtypes. Supplementary Data Fig. 1a provides additional differentially expressed genes for fibroblast subtypes. **d**, Summary of skin fibroblast subtypes in healthy steady-state tissue. Illustrations in **a** and **d** were partly created using BioRender.com.

**Extended Data Fig. 1:**
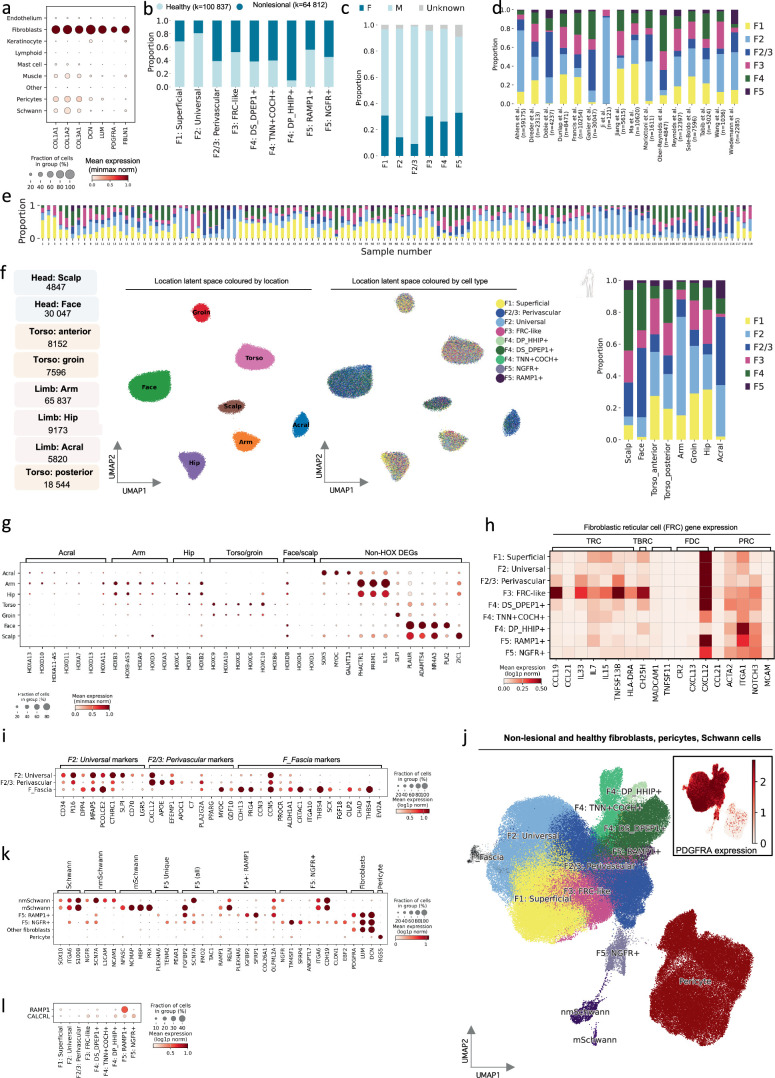
Metadata and integration with Schwann cells, pericytes, and fascial cells. **a**) Marker genes for fibroblast selection**. b**) Proportion of fibroblasts by site status, **c**) sex, **d**) dataset, and **e**) sample. **f**) Anatomical location latent space generated by CellDISECT colored by anatomic location and cell subtype (see Methods). Proportion of fibroblasts by anatomic location. **g**) Differentially expressed HOX genes and other differentially expressed genes by anatomic site using CellDISECT anatomic site clusters. **h**) Heatmap of reported marker genes for fibroblastic reticular cell (FRC) subsets by skin fibroblast subtype. TRC: T reticular cell. TBRC: T-B border reticular cells. FDC: Follicular dendritic cell. PRC: Perivascular reticular cell**. i**) Dotplot of marker genes for fascial fibroblasts and expression of *F2* and *F2/3* markers**. j**) UMAP of additional integration including Schwann cells, fascial fibroblasts, and pericytes. Inlet: UMAP color by expression of normalized *PDGFRA* expression (general fibroblast marker). **k**) Dotplot of Schwann cell, pericyte, and *F5: Schwann-like* fibroblast gene expression. **l**) Dotplot of expression of CGRP receptor components by fibroblast subtype.

We re-processed and integrated 2.1 million cells from scRNA-seq data of adult human skin, comprising 32 datasets and 251 donors (Fig. 1a and Supplementary Table 1)^15,16,18–47^ using single-cell variational inference (scVI) (Methods)^48^. After quality control, 357,276 high-quality fibroblasts were selected based on canonical marker gene expression (Fig. 1a and Extended Data Fig. 1a).

**Fig. 2:**
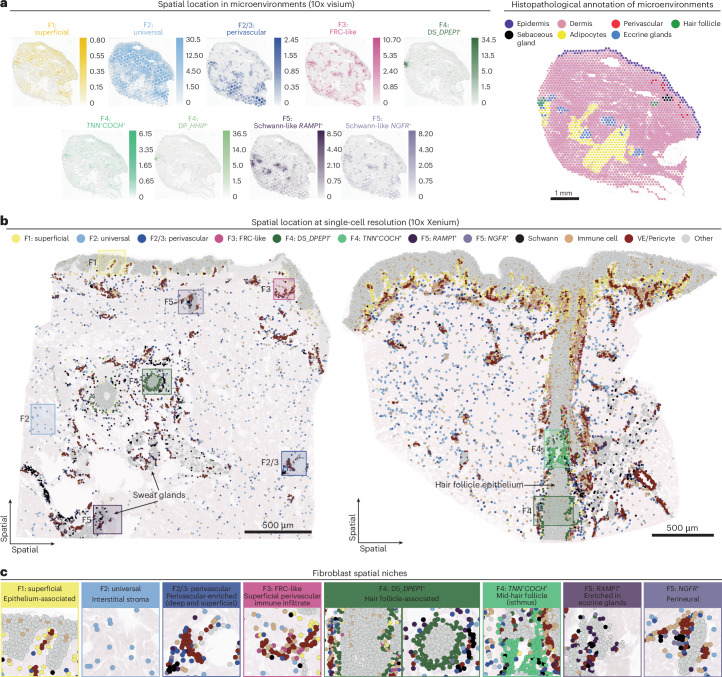
Skin fibroblasts occupy unique spatial and functional niches. **a**, Spatial location of fibroblast subtypes in microenvironments (cell2location abundance predictions (10x Genomics Visium)) in a single section of healthy human skin (left). Histopathological annotation of tissue microenvironments (right). **b**, Spatial location of fibroblasts at single-cell resolution (10x Genomics Xenium 5000-gene panel) for skin sections from nonlesional skin of atopic dermatitis (noninflamed (left) and noninflamed post-treatment (right)), colored by cell type. **c**, Summary of fibroblast niches: Xenium cell types overlying H&E-stained image (manual approximations).

**Extended Data Fig. 2:**
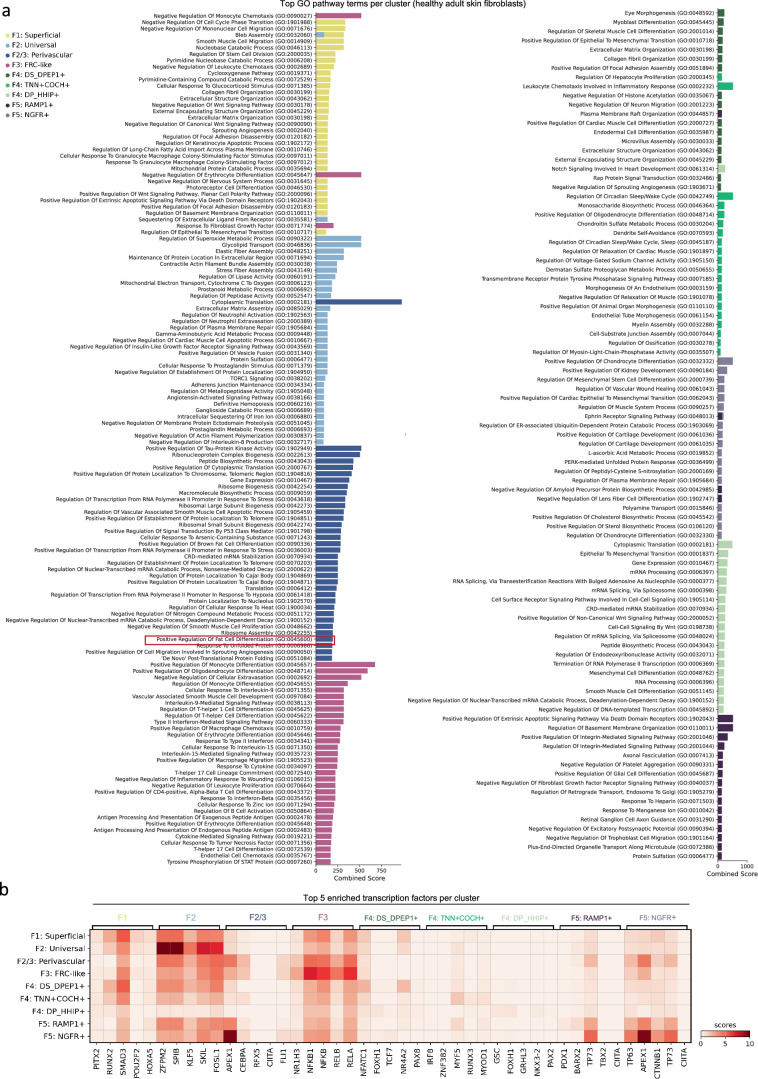
Pathway analysis and transcription factor enrichment for healthy fibroblast populations. **a**) Gene set enrichment analysis for healthy/nonlesional fibroblasts using GSEAPY**. b**) Heatmap of transcription factor activity inference.

**Extended Data Fig. 3:**
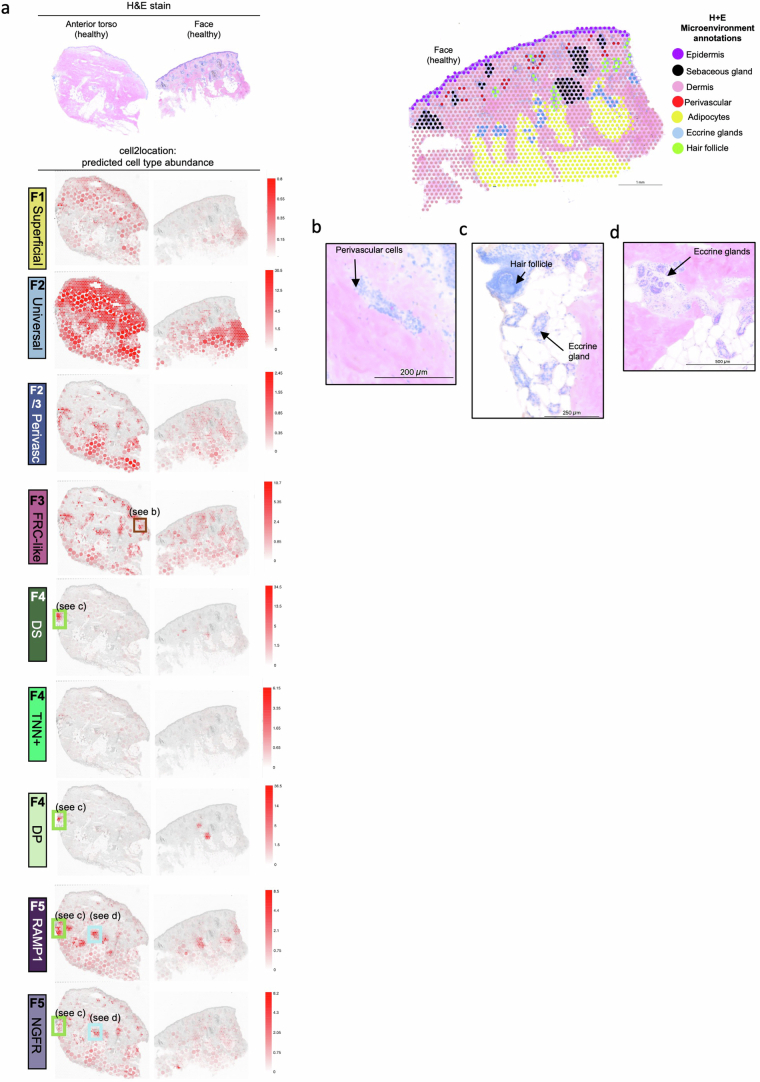
Fibroblast location by deconvolution of fibroblast spots. **a**) H&E stain of skin slides used for 10x Genomics Visium, cell type abundances predicted by cell2location, and histopathological annotations. H&E: Hematoxylin and eosin **b-d**) Close-up regions of H&E stained image for regions annotated in panel **a**.

**Extended Data Fig. 4:**
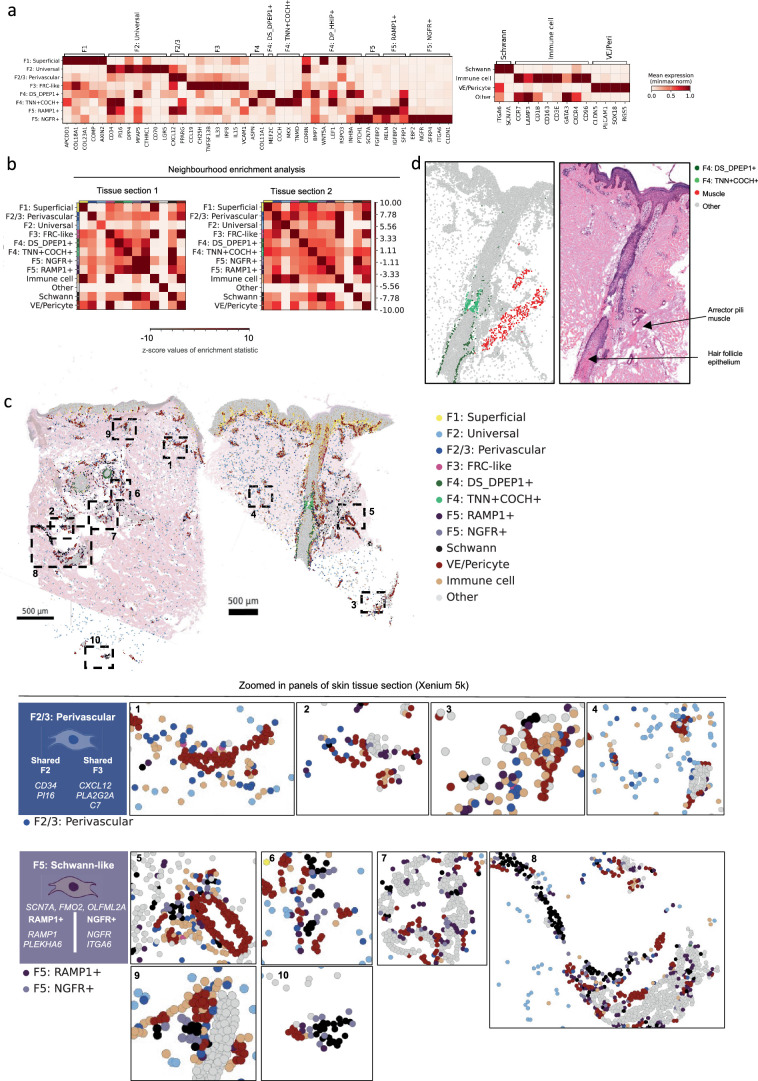
Additional Xenium data. **a**) Heatmap of marker gene expression in 10x Genomics Xenium data for fibroblast and non-fibroblast populations. **b**) Neighborhood enrichment scores for each tissue section shown in Fig. 2b. **c**) 10x Genomics Xenium section of nonlesional atopic dermatitis skin highlighting the location of *F2/3: Perivascular* and *F5: Schwann-like* fibroblasts at higher zoom. **d**) Xenium data colored by cell type annotation and H&E stain of skin indicating position of *F4: TNN* + *COCH* + , superior to the insertion of the arrector pili muscle.

In healthy skin, we identified six major fibroblast subtypes based on differential gene expression (Supplementary Data Fig. 1a and Supplementary Table 2) and pathway enrichment analysis (Extended Data Fig. 2a and Methods). The six fibroblast subtypes were observed across different covariates (Extended Data Fig. 1b–g and Supplementary Note 1). Complementary spatial transcriptomic methods validated the presence of each of the six fibroblast subtypes and revealed their distinct microanatomical locations (Fig. 2a–c, Extended Data Figs. 3 and 4 and Supplementary Fig. 2).

Two of the six fibroblast populations (F1: superficial (papillary) and F2: universal (reticular)) were uniformly present throughout skin at different tissue depths. F1: superficial (papillary) fibroblasts localized adjacent to the skin epithelium in the papillary dermis (Fig. 2b,c) and expressed genes encoding superficial dermal collagens (*COL13A1*, *COL18A1* and *COL23A1*) and Wnt signaling inhibitors (*APCDD1*, *WIF1* and *NKD2*) (Fig. 1c). A Wnt-mediated synergistic interplay between superficial dermal fibroblasts and basal epithelial cells has been reported to reciprocally maintain cellular identity^14,49^.

F2: universal (reticular) fibroblasts were located deeper in the skin, interspersed between large collagen fibers in the reticular dermis (Fig. 2b,c). This population was characterized by high expression of marker genes of universal PI16^+^ fibroblasts (*PI16*, *CD34* and *MFAP5)*^4^, a fibroblast subtype found in many human tissues and postulated to represent a precursor fibroblast cell state^4,50^. Transcription factor activity inference^51^ identified *KLF5* in F2: universal fibroblasts (Extended Data Fig. 2b), which has been reported to drive the universal Pi16^+^ state^52^. As fascial fibroblasts (F_Fascia) are proposed as a potential progenitor cell in mouse skin^53^, we included these cells in an additional integration, identifying that F_Fascia formed a subset of F2: universal (Extended Data Fig. 1i,j and Supplementary Note 2).

The remaining fibroblast subsets were more focal in localization, being associated with vascular or adnexal structures. We thus used hematoxylin and eosin (H&E) staining to illustrate these microenvironments. F3: fibroblastic reticular cell (FRC)-like fibroblasts were located predominantly in the superficial perivascular region in proximity to immune cells (Fig. 2b and Extended Data Figs. 3a,b and 4a,b). F3: FRC-like fibroblasts transcriptomically resembled FRCs, which are specialized fibroblasts found in lymphoid organs/structures that maintain immune niches (Extended Data Fig. 1h)^54^, expressing genes that attract and compartmentalize immune cells (*CCL19*, *CXCL12* and *CH25H*), maintain immune cell survival and function (*IL33*, *IL15*, *TNFSF13B* and *VCAM1*) and enable antigen presentation (*CD74* and major histocompatibility complex (MHC)-II molecules)^54^.

**Fig. 3:**
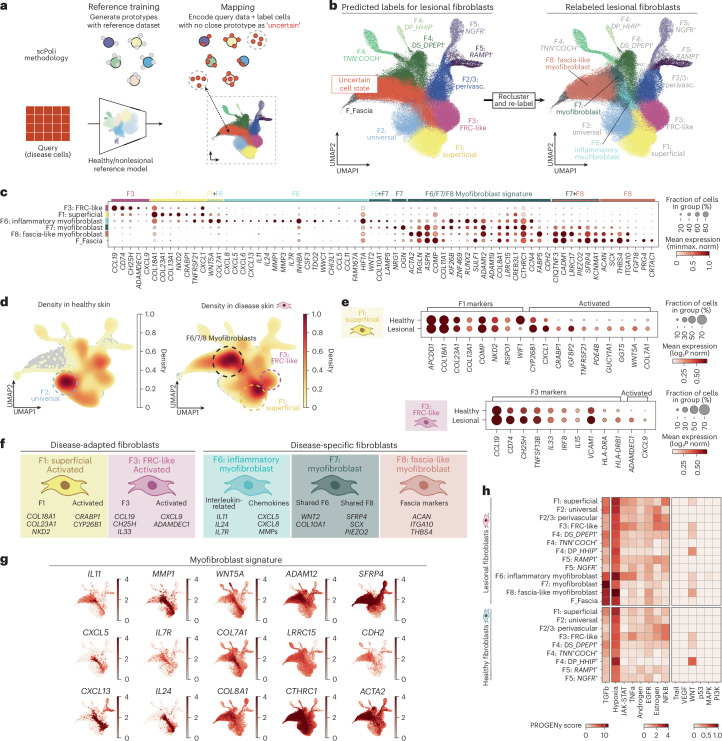
Prototype meta-learning to identify disease-adapted and disease-specific populations. **a**, Overview of reference-mapping approach used for integration, where lesional/diseased data were mapped using a pretrained model. **b**, UMAP of scPoli embeddings colored by predicted cell-type labels and relabeled populations after re-clustering. **c**, Dotplot of marker gene expression for disease-adapted and disease-specific fibroblast populations. Supplementary Data Fig. 1c provides additional differentially expressed genes for disease-associated fibroblast subtypes. **d**, Density of cells in embedding by site status. **e**, Gene expression in F1 and F3 fibroblasts from health and disease, including differentially expressed genes in lesional/diseased states. **f**, Summary of disease-adapted and disease-specific populations. **g**, Feature maps for genes associated with myofibroblast subtypes. Color bars indicate expression (log_1_*P* norm). **h**, PROGENy pathway scores for fibroblasts from lesional and healthy samples. Illustrations in **f** were partly created using BioRender.com.

F2/3: perivascular fibroblasts also localized with immune cells but, unlike F3: FRC-like fibroblasts, were additionally enriched in deep perivascular regions and other sites (Fig. 2a–c and Extended Data Fig. 4b,c). A fraction of F2/3: perivascular fibroblasts showed elevated expression of *PPARG* (Fig. 1c) and pathway analysis suggested a role in adipocyte differentiation (Extended Data Fig. 2a). The capability to differentiate into adipocytes is characteristic of the reticular fibroblast (equivalent to F2: universal) lineage^14,55,56^. F2/3: perivascular fibroblasts shared select gene expression with both F2: universal and F3: FRC-like fibroblasts (Fig. 3c).

F4: hair follicle-associated fibroblasts (*ASPN*^+^*COL11A1*^+^) encompassed three subclusters that were associated with specific regions of the hair follicle (Fig. 2b,c). The first is a well-characterized^19^ dermal sheath (DS) population (F4: DS_DPEP1^+^) that wraps around the lower/mid hair follicle (Fig. 2b). The second is a novel F4: *TNN*^+^*COCH*^+^ subtype, expressing tendon-associated genes (*MKX* and *TNMD*) and observed at the isthmus (mid-hair shaft) (Fig. 2b and Extended Data Fig. 4d). The third F4: DP_HHIP^+^ subtype uniquely expressed dermal papilla marker genes (*CORIN*, *HHIP*, *RSPO3* and *LEF1*)^57–59^.

F5: Schwann-like fibroblasts (SCN7A, *FMO2*, *FGFBP2* and *OLFML2A*) contained two subclusters (F5: *NGFR*^+^ and F5: *RAMP1*^+^) (Extended Data Fig. 1k)*.* F5: *RAMP1*^+^ fibroblasts were enriched near innervated eccrine glands and expressed genes encoding the receptor complex for the neuropeptide *CGRP* (Fig. 2b,c and Extended Data Figs. 1l, 3c,d and 4c), suggesting a possible interface with the nervous system. F5: *NGFR*^+^ colocalized with Schwann cells, suggesting that they are a nerve-associated population. Fibroblasts have been described in the endoneurium and perineurium of nerve fibers from imaging studies^60^, and ‘Schwann-like fibroblasts’ have recently been reported in human skin scRNA-seq data^17^.

We confirmed that our six fibroblast subtypes were distinct from Schwann cells and pericytes (Extended Data Fig. 1j,k and Supplementary Note 2). In addition, we harmonized our skin fibroblast annotation with a previous classification^15^ (Supplementary Data Fig. 1b).

Overall, we provide a new framework for healthy human skin fibroblast annotation based on gene expression profiles (Fig. 1) and spatial location (Fig. 2) that integrates previous fibroblast descriptions in skin and across tissues. Our findings of transcriptionally defined fibroblast subtypes in distinct microanatomical locations suggest a role for regional fibroblasts in supporting distinct niche functions.

### Skin disease-adapted and disease-specific fibroblasts

We next sought to identify how fibroblast states change in diseased skin. We used scPoli^61^, a deep-learning model for integration and identification of novel cell states in single-cell transcriptome data (Methods) (Fig. 3a).

**Extended Data Fig. 5:**
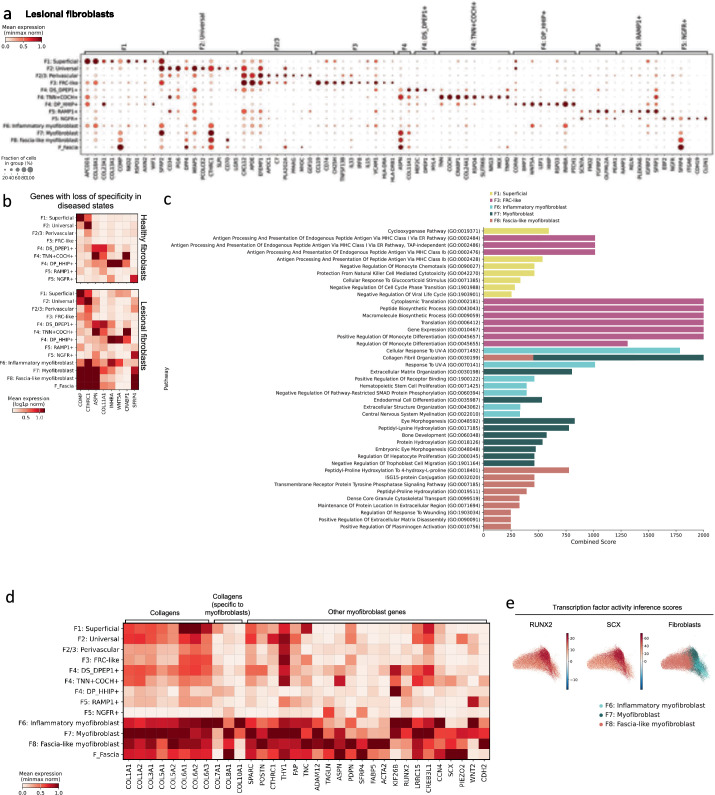
Disease-adapted and disease-specific fibroblast additional data. **a**) Dotplot of expression of marker genes used for healthy fibroblasts in lesional/diseased skin fibroblasts**. b**) Heatmap of genes that were used as marker genes for healthy fibroblasts but that were less specific when diseased fibroblasts were included. **c**) Gene set enrichment analysis for disease-associated fibroblast populations. **d**) Heatmap of gene expression for select collagens, common fibroblast activated genes, and additional markers for skin myofibroblasts. **e**) UMAP visualization of transcription factor activity scores (*RUNX2*, *SCX*).

We mapped fibroblasts from skin diseases to our healthy/nonlesional F1–F5 fibroblast reference. Out of 190,756 fibroblasts from diseased states, 121,167 diseased cells were confidently assigned existing F1–F5 cell labels (Extended Data Fig. 5a,b). The remaining 69,589 fibroblasts from the disease data were classified as uncertain (unlabeled) by scPoli (Fig. 3b). Manual annotation based on differential gene expression (Supplementary Data Fig. 1c and Supplementary Table 3) and pathway analysis (Extended Data Fig. 5c) revealed two ‘disease-adapted’ and three ‘disease-specific’ fibroblast subtypes (Fig. 3b–f).

‘Disease-adapted’ fibroblasts resembled a healthy fibroblast subtype counterpart (Fig. 3e) and were expanded in disease settings (Fig. 3d). The first disease-adapted fibroblast subtype resembled F1: superficial fibroblasts in healthy skin (Fig. 3e). The F1-like disease population upregulated genes suggestive of regenerative function (*CRABP1*, *CYP26B1* and *WNT5A*)^53,62,63^. *CRABP1* and *CYP26B1* are markers of superficial/upper wound fibroblasts in mice^53,62^, which are thought to be the source of wound-induced hair follicle neogenesis^64^, and involved in retinoic acid degradation. *CRABP1*^+^ fibroblasts are also associated with regeneration in reindeer skin^65^ and early-gestational human skin^66^.

The second disease-adapted fibroblast subtype resembled F3: FRC-like fibroblasts and upregulated *CXCL9* and/or *ADAMDEC1* (Fig. 3e). *CXCL9* is a chemoattractant for *CXCR3*^+^ cells and has been reported as an activation marker for *FRCs* in lymphoid tissues^54,67^.

‘Disease-specific’ fibroblasts (F6: inflammatory myofibroblasts, F7: myofibroblasts and F8: fascia-like myofibroblasts) did not have a healthy skin fibroblast counterpart and highly expressed a myofibroblast gene signature. This myofibroblast signature included contractility (*ACTA2*), extracellular matrix (ECM) (*COL3A1*, *COL5A1*, *COL8A1*, *POSTN* and *CTHRC1*) and other myofibroblast-associated genes (*LRRC15*, *SFRP4*, *ASPN*, *RUNX2* and *SCX*) (Fig. 3c,g and Extended Data Fig. 5d,e)^16,68,69^.

F6: inflammatory myofibroblasts additionally expressed immune-related genes such as interleukins (*IL11* and *IL24*), chemokines (*CXCL5*, *CXCL8*, *CXCL13* and *CCL11*) and matrix metalloproteinases that can remodel tissue to facilitate immune cell infiltration (*MMP1*)^70^ (Fig. 3c). JAK–STAT and hypoxic signaling genes were also elevated (Fig. 3h).

F7: myofibroblasts and F8: fascia-like myofibroblasts were distinguished by a higher expression of ECM and TGFβ signaling genes, as well as the mechanotransducer *PIEZO2* (Fig. 3c,h). F8: fascia-like myofibroblasts were distinguished by expression of F_Fascia-associated genes (Fig. 3c).

Overall, our results indicate that healthy fibroblasts can acquire a regenerative phenotype in F1: superficial fibroblasts (*CRABP1*^+^*CYP27B1*^+^), a distinct polarization in F3: FRC-like fibroblasts (*CXCL9*^+^/*ADAMDEC1*^+^) and potentially give rise to myofibroblast states (*ACTA2*^+^*COL8A1*^+^*SFRP4*^+^) in diseased skin.

### Fibroblast compositional signatures characterize distinct scarring risk stroma

**Fig. 4:**
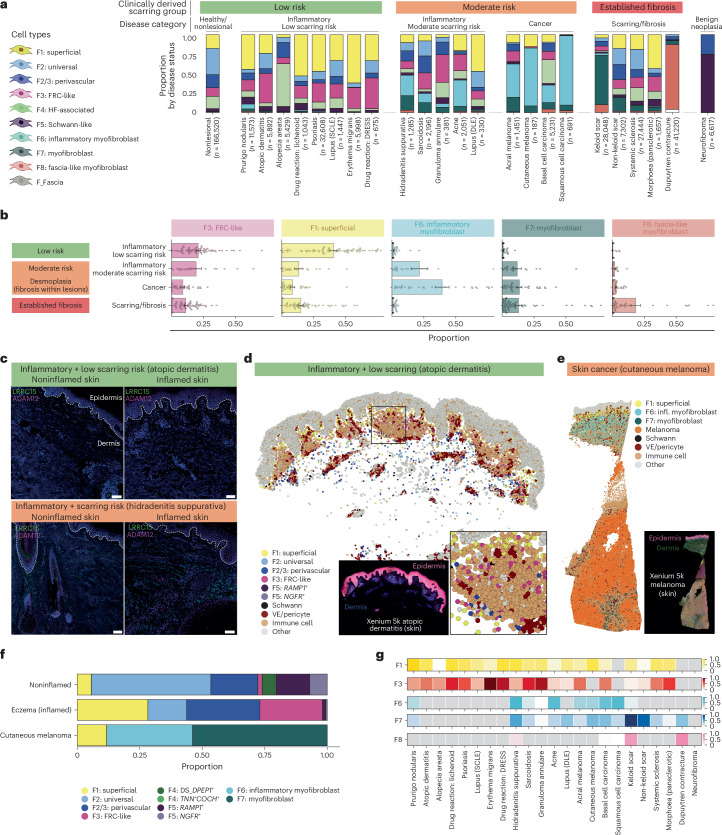
Fibroblast compositional signatures characterize the stroma of distinct skin diseases and scarring risk categories. **a**, Proportion of fibroblast populations by individual disease. Labels overlying each bar indicate the disease category. **b**, Proportion of disease-adapted and disease-specific fibroblast subtypes by disease category (mean ± s.e.m.). Scarring risk group was based on clinical profiles (Methods). **c**, Immunofluorescence of LRRC15 (green) and ADAM12 (magenta) showing myofibroblast populations only in inflamed hidradenitis suppurativa skin (right-most) (from two representative atopic dermatitis and hidradenitis suppurativa inflamed and noninflamed samples). Scale bar, 100 µm. **d**, Xenium 5k data for lesional/inflamed atopic dermatitis skin, with cells colored by cell type. **e**, Xenium 5k data for cutaneous melanoma, with cells colored by cell type. **f**, Proportion of fibroblast populations by disease status for Xenium 5k data. **g**, Gene module scores for each disease-associated fibroblast subtype across diseases with row normalization (0–1). VE, vascular endothelium; SCLE, subacute cutaneous lupus erythematosus; DLE, discoid lupus erythematosus.

**Extended Data Fig. 6:**
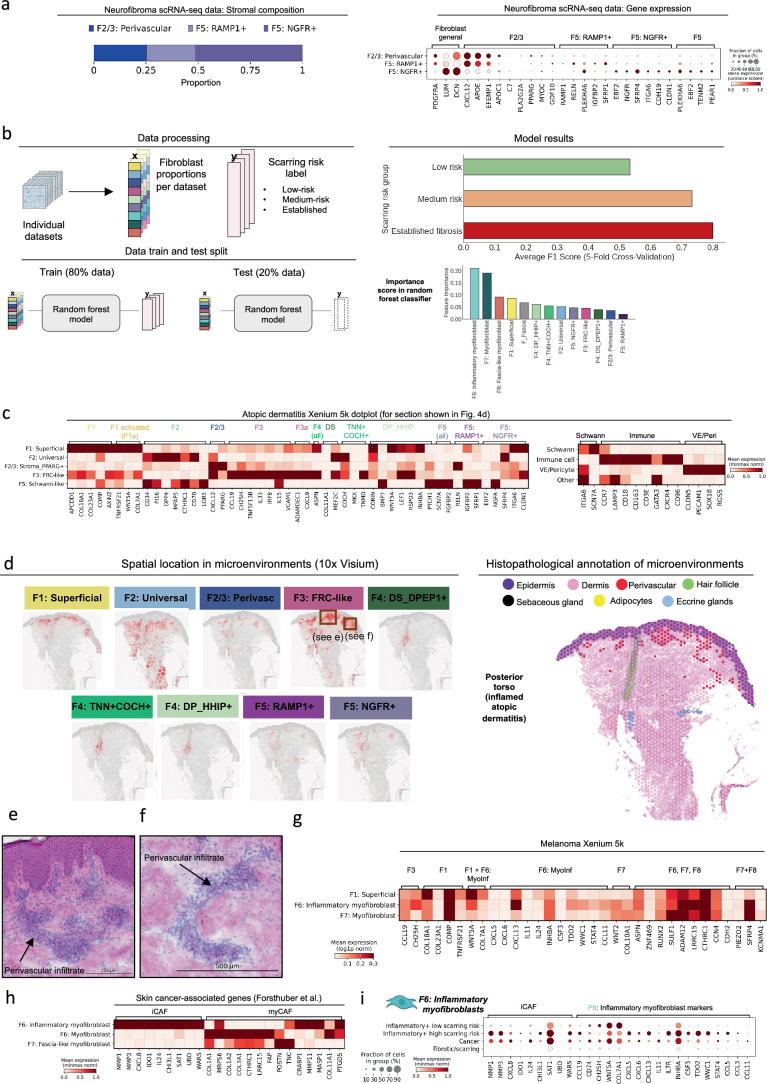
Disease stroma profiles. **a**) Fibroblast proportions in neurofibroma and dotplot of expression of *F2/3* and *F5* marker genes for fibroblasts in neurofibroma. **b**) Methodology for random forest classifier. Results: F1-score for scarring risk category classification and importance scores by fibroblast subtype**. c**) Heatmap of marker genes for fibroblast and non-fibroblast populations in Xenium 5k data for lesional/inflamed atopic dermatitis skin. **d**) Cell2location predictions and histopathological annotation for lesional (inflamed) atopic dermatitis skin profiled by 10x Visium. Same scale used for fibroblast subtypes as shown in Extended Data Fig. 3a**. e,f**) Zoomed in regions of perivascular inflammatory infiltrate (predicted location of *F3: FRC-like* fibroblasts). **g**) Heatmap of marker genes for fibroblast populations in Xenium 5k data for cutaneous melanoma. CAF: cancer-associated fibroblast. **h**) Heatmap of expression of skin CAF genes from Forsthuber et al. in our skin fibroblast subtypes (scRNA-seq data). **i**) Dotplot of expression of inflammatory CAF marker genes and our reported *F6: Inflammatory myofibroblast* marker genes in *F6: Inflammatory myofibroblasts* from different disease categories.

We next leveraged the diverse clinical profiles of skin diseases to assess whether fibroblast subtypes provide molecular insights into disease endotypes with respect to scarring. We assigned the 23 skin diseases into three clinically determined risk of scarring groups: low scarring risk, moderate scarring risk, and established scarring/fibrosis (see Methods) (Fig. 4a). We excluded neurofibroma from this analysis as it was the only case of benign neoplasia, consisting primarily of F5: Schwann-like and F2/3: perivascular fibroblasts (Extended Data Fig. 6a).

We identified distinct fibroblast compositions for each scarring risk category (Fig. 4b). Low scarring risk diseases were characterized by a high prevalence of F1: superficial (*CRABP*^+^*CYP27B1*^+^) and F3: FRC-like fibroblasts (*CXCL9*^+^/*ADAMDEC1*^+^) (Fig. 4b), without notable F6–F8 myofibroblast populations. This finding agrees with the regenerative-associated gene profile of disease-associated F1: superficial fibroblasts and a role for F3: FRC-like fibroblasts in maintaining immune niches.

Diseases with scarring risk were characterized by a uniquely high prevalence of F6: inflammatory myofibroblasts, which was not observed in low scarring risk or established fibrosis (Fig. 4b). F7: myofibroblasts were observed at a similar prevalence in diseases with scarring risk and established fibrosis. These data point toward F6: inflammatory myofibroblast as a population influencing scarring risk, but which are largely absent in established fibrosis. F8: fascia-like myofibroblasts were also elevated in established fibrosis but were predominantly observed in Dupuytren contracture, a fibroproliferative disease of the palmar fascia (Fig. 4a).

We used two further approaches to demonstrate the role for distinct fibroblast subtypes predicting scarring risk. First, we trained a random forest classifier and identified that F6: inflammatory myofibroblasts and F7: myofibroblasts were the most important fibroblast subtypes for predicting scarring risk category (Extended Data Fig. 6b). Second, we profiled a well-recognized myofibroblast marker (LRRC15)^68^ at the protein level. LRRC15 was evident in inflammation with scarring risk (inflamed hidradenitis suppurativa skin) but not in noninflamed skin or inflamed skin without scarring risk (atopic dermatitis skin) (Fig. 4c).

Having established that disease-associated fibroblasts are enriched in distinct scarring categories, we next used spatial transcriptomics to validate these fibroblast populations in distinct scarring risk stroma (Fig. 4d–f and Supplementary Fig. 3)^71^. In keeping with scRNA-seq data (Fig. 4a), F3: FRC-like fibroblasts were expanded in inflamed atopic dermatitis skin (low risk), without major myofibroblasts (Fig. 4d,f and Extended Data Fig. 6c). We localized the F3: FRC-like population to the superficial perivascular immune niche (Fig. 4d), which we further validated using 10x Visium data (Extended Data Fig. 6d–f). In melanoma (scarring risk), aside from F1, the entire stroma comprised F6: inflammatory myofibroblasts and F7: myofibroblasts (Fig. 4e,f and Extended Data Fig. 6g). F7: myofibroblasts showed a matrix-producing phenotype (*COL1A1*, *COL3A1* and *POSTN*) that characterizes myofibroblastic cancer-associated fibroblasts (CAFs) (myoCAFs)^72,73^. F6: inflammatory myofibroblasts demonstrated high expression of inflammatory CAF (iCAF) marker genes^72^ (*MMP1*, *MMP3*, *CXCL8* and *IL24*), which was observed in both cancer and inflammatory diseases with scarring risk (Extended Data Fig. 6h,i).

Finally, to complement our analysis of fibroblast proportions by disease, we assessed transcriptomic variability of disease-associated fibroblast subtypes by calculating gene module scores for each disease using defined marker genes to define transcriptomic variability across different disease conditions (Fig. 4g and Methods). The F6: inflammatory myofibroblast signature score was highest in hidradenitis suppurativa, acne and keratinocytic skin cancers.

Overall, our findings support distinct stromal composition in skin diseases associated with differential scarring risk. F6: inflammatory myofibroblasts were observed in diseases with scarring risk but relatively infrequently observed in established fibrosis, raising the possibility that they may be an intermediate differentiation state toward F7: myofibroblasts.

### Origin of skin disease-specific myofibroblasts

The differentiation process of healthy fibroblasts into myofibroblasts remains poorly understood in human tissues despite its clinical relevance. Fibroblasts are tissue resident, and thus intermediate states of myofibroblast differentiation are likely to be captured in the molecular snapshots of skin diseases analyzed. We therefore performed trajectory analysis of fibroblasts in diseased skin to gain further insights into myofibroblast differentiation, before utilizing time-resolved human wound data as a validation of dynamic changes in stromal composition.

**Fig. 5:**
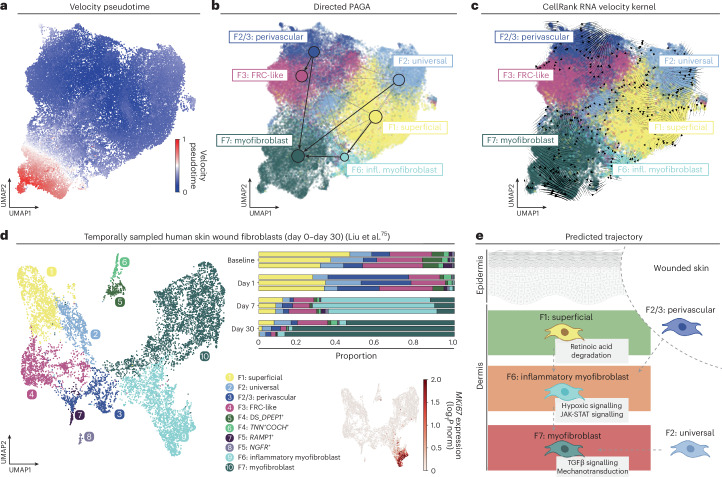
Origin of skin disease-specific fibroblast subtypes. **a**–**c**, Velocity pseudotime (**a**), directed PAGA overlaid on UMAP (**b**) and velocity kernel from CellRank2 for lesional fibroblasts (**c**). For further details see Methods. **d**, UMAP visualization of fibroblast subtypes from human skin wounds data colored by cell type (left) and MKI67 (encodes Ki-67) expression (bottom right). Proportions of fibroblast populations by time point (top right), where each bar represents a donor at a given time point. **e**, Schematic of predicted trajectories. Dashed arrows indicate predictions with multiple lines of evidence. Fibroblast populations are colored by the predominant scarring/fibrosis risk observed in an earlier analysis: green (prevalent in low-risk scarring stroma), orange (prevalent in scarring risk stroma and cancer), red (prevalent in established scarring/fibrotic disorders). Gray boxes indicate signaling pathways identified in our gene expression/pathway analysis. Schematic in **e** was partly created using BioRender.com.

**Extended Data Fig. 7:**
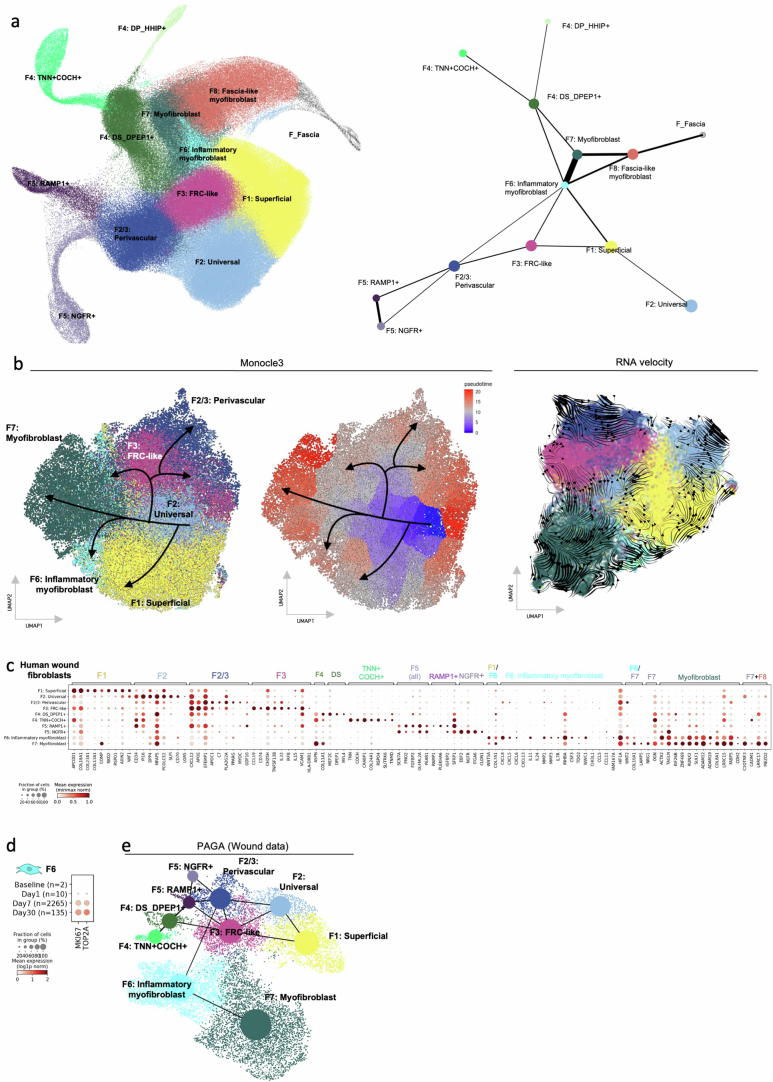
Additional trajectory inference. **a**) UMAP and partition-based graph abstraction (PAGA) for all fibroblast subtypes. **b**) Trajectory inference from Monocle 3 (using *F2: Universal* as the root state) (left), pseudotime (middle), and RNA velocity from scVelo (right)**. c**) Marker gene expression for fibroblasts identified from human wound data. **d**) Dotplot of MKI67 expression by time point in *F6: Inflammatory myofibroblasts*. **e**) PAGA analysis of human wound data.

We first included all fibroblast subtypes in a partition-based graph abstraction (PAGA) analysis (Extended Data Fig. 7a), and then focused further analyses on fibroblast populations found across diseases on hair-bearing and hairless skin (Methods). F7: myofibroblasts were a terminally differentiated myofibroblast state (Fig. 5a–c), consistent with their presence in established fibrosis. We observed two potential sources for F7: myofibroblasts in skin across analyses (Fig. 5b,c and Extended Data Fig. 7b). One trajectory arose directly from the F2: universal lineage. A second trajectory originated from F1: superficial fibroblasts, transitioning to F7: myofibroblasts via an intermediate F6: inflammatory myofibroblast state (Fig. 5a–c and Extended Data Fig. 7b). These two inferred trajectories are consistent with in vivo lineage tracing studies in mice^53,55,74^.

To investigate these predicted trajectories in real time, we leveraged a human skin wound dataset of 58,823 cells (Methods)^75^. Skin tissue had been collected from healthy human volunteers at baseline (pre-wound) and subsequently from healing wounds. At baseline, myofibroblasts were not present, but on day 1 post-wounding, a small number of F6: inflammatory myofibroblasts were observed (Fig. 5d and Extended Data Fig. 7c). By day 7, F6: inflammatory myofibroblasts were the predominant population. By day 30, F7: myofibroblasts had become the predominant population, consistent with a role in established fibrosis/scarring. Overall, our results point toward F6: inflammatory myofibroblasts as an intermediate differentiation state toward F7: myofibroblasts in human skin, with potential plasticity in fibroblast origin (Fig. 5d,e and Extended Data Fig. 7e).

### Human cross-tissue disease fibroblast populations

We next investigated if the skin fibroblast subtypes we identified were conserved across other human tissues. Previous studies have reported fibroblast states that are found across human tissues^4–6^. However, because these studies each defined fibroblast subtypes with different nomenclature and gene markers, it is unclear how these populations relate to each other and to the skin fibroblast populations reported in our study. To answer this and perform an overarching analysis across tissues and diseases, we undertook two approaches.

**Fig. 6:**
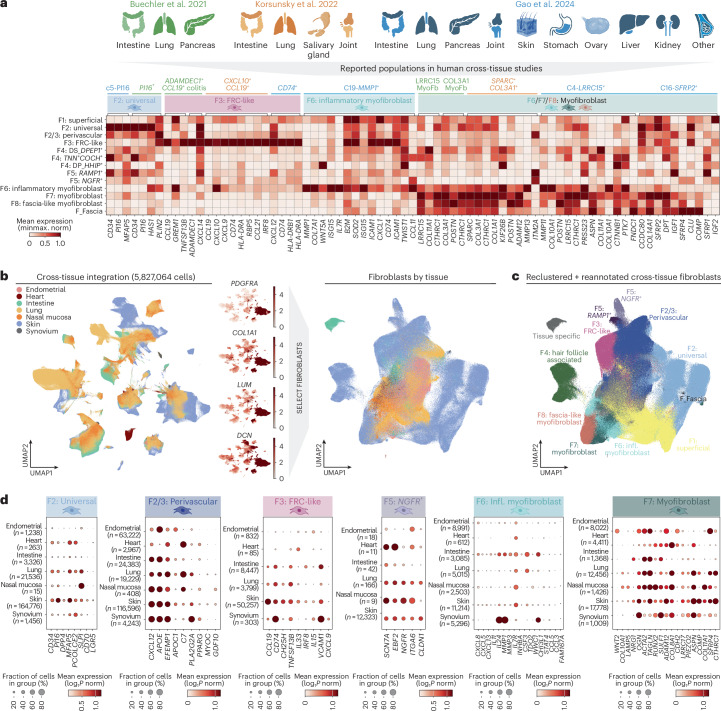
Human cross-tissue disease fibroblast populations. **a**, Human tissues previously included in cross-tissue fibroblast studies and the fibroblast subtypes they have identified (above heatmap), colored by study (Buechler et al.^4^ in green, Korsunsky et al.^6^ in orange and Gao et al.^5^ in blue). Heatmap shows gene expression of marker genes previously reported for cross-tissue fibroblast populations in our lesional skin fibroblast subtypes. Immediately above the heatmap we show the skin fibroblast subtype with most similar gene expression to reported cross-tissue populations. **b**, UMAP visualization for cross-tissue integration (left) and for fibroblasts specifically (right), colored by tissue. Color bars indicate expression (log_1_*P* norm). **c**, UMAP visualization for fibroblasts colored by re-annotated clusters (Methods). **d**, Dotplots of expression of marker genes we previously used for skin fibroblasts in cross-tissue atlas clusters by tissue type. Note that not all genes were available as the endometrial dataset contained ~17,000 genes. Illustrations in **a** were partly created using BioRender.com.

**Extended Data Fig. 8:**
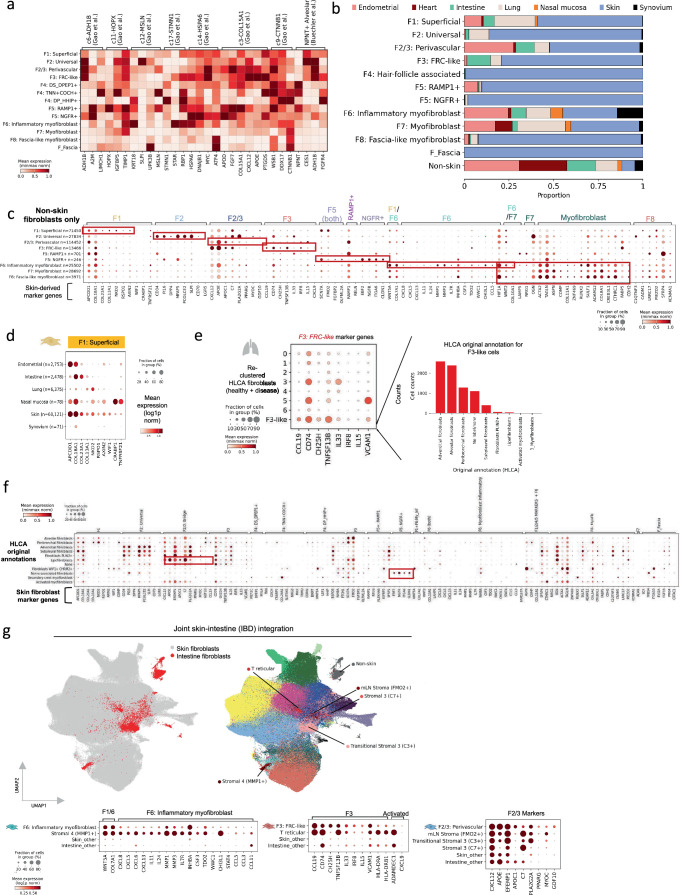
Additional cross-tissue populations and human skin-intestine fibroblast integration. **a**) Heatmap of marker genes for additional reported shared/universal fibroblast populations from Gao et al. **b**) Proportion of fibroblasts by tissue in renamed fibroblast clusters from cross-tissue atlas. **c**) Dotplot of skin fibroblast marker genes for cross-tissue fibroblast clusters (excluding skin fibroblasts). Skin fibroblasts were excluded to avoid skin fibroblasts driving the gene expression signature for each cluster. **d**) Expression of *F1* marker genes by tissue in fibroblasts annotated as *F1: Superficial f*ibroblasts in our cross-tissue atlas. **e**) Dotplot of *F3* marker gene expression in clustered HLCA data and bar plot showing original annotations for fibroblasts that we re-annotated as ‘F3-like’. HLCA: Human Lung Cell Atlas. **f**) Dotplot of skin marker genes in HLCA data (original annotations from HLCA). **g**) UMAP visualization for joint human skin-intestine fibroblast integration using labeled data from the gut atlas (Elmentaite et al). Dotplot of marker genes for *F6: Inflammatory myofibroblasts* (from skin) in *F6: Inflammatory myofibroblasts* (skin) and *Stromal 4 (MMP1*+*)* (intestine) populations; dotplot of marker genes for *F3: FRC-like* (from skin) in *F3: FRC-like* (skin) and *T reticular cells (*intestine) populations (same scale for both plots); and dotplot of marker genes for *F2/3: Perivascular* in *F2/3: Perivascular* (skin) and various intestinal populations (same scale for all plots).

The first was to assess the expression of marker genes for cross-tissue fibroblast subtypes in our skin data^4–6^. This identified that reported cross-tissue populations from previous studies are likely present in human skin, consistent with F2: universal, F3: FRC-like, F6: inflammatory myofibroblast, and F7: myofibroblast subtypes (Fig. 6a and Extended Data Fig. 8a).

The second approach was to integrate published datasets of ~5.8 million cells from human skin, lung, intestine, synovium, endometrium, heart and nasal mucosa (Methods and Fig. 6b). This approach uses the whole transcriptome profile, instead of restricted marker genes, and thus more comprehensively defines cell state similarity. Approximately 1 million fibroblasts were selected for downstream analysis based on expression of canonical marker genes (Fig. 6b). In the cross-tissue integrated dataset, we were able to discern shared fibroblast states across tissues, as well as fibroblasts that were unique to certain tissues (Fig. 6b,c and Extended Data Fig. 8b). In addition to known cross-tissue populations identified above (Fig. 6a), we found evidence for F2/3: perivascular (*CXCL12*, *APOC1* and *PPARG*) and F5*: NGFR*^+^ (Schwann-like; *SCN7A*, *NGFR*, *ITGA6* and *EBF2*) fibroblasts across human tissues, including in lung, gut and nasal mucosa (Fig. 6c,d and Extended Data Fig. 8b,c). Further interrogation of labeled intestine and lung datasets supported these results (Supplementary Note 3). F1: superficial showed variable gene expression by tissue, which may reflect distinct epithelia patterning across sites (Extended Data Fig. 8d).

Overall, our results point toward the presence of previously reported cross-tissue fibroblast states in skin, despite major differences in the biophysical properties of different human tissues. We additionally suggest F2/3: perivascular and F5: *NGFR*^+^ (nerve-associated) fibroblasts as novel cross-tissue populations.

### Cross-tissue fibroblasts regulate distinct immune niches in skin

Fibroblast-mediated processes such as fibrosis and maintenance of immune cell niches are observed across multiple human tissues. We therefore asked whether disease-associated fibroblast states identified in skin were similarly enriched by disease category in non-skin tissues (Methods). We focused on F3: FRC-like and F6: inflammatory myofibroblasts based on their conserved states across tissues and potential immune-interacting roles from pathway enrichment analysis (Extended Data Figs. 2a and 5c). Then, to predict functional interactions with immune cells, we utilized our skin data to perform cell–cell communication analysis.

**Fig. 7:**
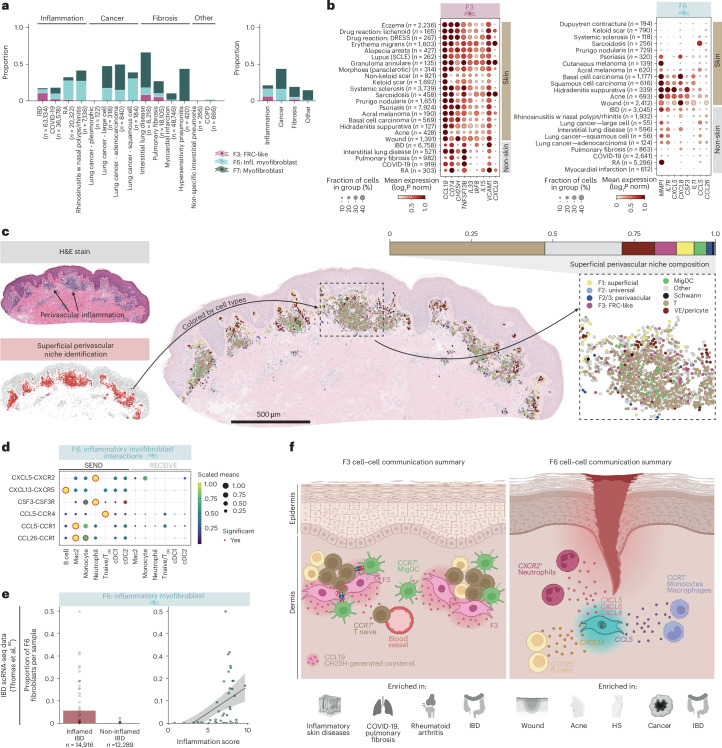
Cross-tissue F3: FRC-like fibroblasts and F6: inflammatory myofibroblasts regulate skin immune niches. **a**, Proportion of disease-associated F3: FRC-like fibroblasts, F6: inflammatory myofibroblasts and F7: myofibroblasts in non-skin tissues by disease (left) and disease category (right). **b**, Dotplot of F3: FRC-like expression across diseases (skin and non-skin) (left). Dotplot of F6: inflammatory myofibroblasts gene expression across diseases (skin and non-skin) (right). Diseases with a minimum of 50 cells. **c**, H&E slide of lesional atopic dermatitis skin with annotation of perivascular infiltrate regions (top left). Niche identification and proportion of cells in the perivascular superficial niche (bottom left). Composition of the perivascular superficial niche in 10x Genomics Xenium. Insert: zoomed in version of perivascular niche cluster. **d**, Cell–cell communication analysis for F6: inflammatory myofibroblasts and skin immune cells (Methods). T_CM_, T central memory. **e**, Proportion of F6: inflammatory myofibroblasts in IBD by intestinal tissue inflammation status and linear regression of proportion with inflammation scores with 95% CI. **f**, Schematic summary of cell–cell interactions for F3: FRC-like fibroblasts and F6: inflammatory myofibroblasts. Schematic in **f** were created using BioRender.com.

**Extended Data Fig. 9:**
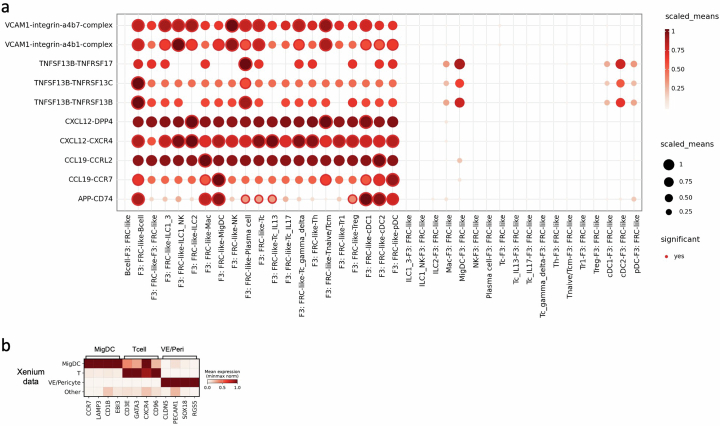
*F3: FRC-like* fibroblast cell–cell communication. **a**) Cell–cell communication results from CellphoneDB, including only significant interactions and genes identified as *F3: FRC-like* marker genes previously. **b**) Dotplot of marker genes for non-fibroblast cells in inflamed atopic dermatitis skin (superficial perivascular niche).

Across tissues, we observed that F3: FRC-like fibroblasts were present in both inflammatory disorders and fibrotic processes with immune-mediated pathology, including lung (COVID-19 and interstitial lung disease) and intestine (inflammatory bowel disease (IBD)) (Fig. 7a,b)^76^. While FRC-like fibroblasts were not reported in the Human Lung Cell Atlas (HLCA), following re-clustering we identified an F3: FRC-like population in lung (Extended Data Fig. 8e,f), consistent with a previous report^77^. We also confirmed equivalence of F3: FRC-like fibroblasts to T reticular cells (an FRC subset) in IBD^78^ (Supplementary Note 3 and Extended Data Fig. 8g). Receptor–ligand analysis suggested interactions of skin F3: FRC-like fibroblasts with migrating dendritic cells (MigDCs) (*CCL19*-*CCR7*) and T cell subsets (*CXCL12*-*CXCR4*) (Extended Data Fig. 9a), suggesting that F3: FRC-like fibroblasts maintain T lymphoid populations and facilitate T cell–dendritic cell interactions analogous to T reticular cells in lymphoid tissue. We corroborated these findings using NicheCompass for niche identification in inflamed atopic dermatitis skin^79^, which revealed *CCR7*^+^ MigDCs and *CXCR4*^+^ T cells within the *F3* superficial perivascular niche (Fig. 7c and Extended Data Fig. 9b).

F6: inflammatory myofibroblasts were abundant in cancer and inflammation but relatively uncommon in established fibrosis (Fig. 7a,b), consistent with skin data (Fig. 4b). Inflammatory myofibroblasts are well described in IBD^80,81^, and we confirmed equivalence of these cells to skin F6: inflammatory myofibroblasts (Supplementary Note 3 and Extended Data Fig. 8g). To further assess the clinical relevance of F6 in IBD, we used an scRNA-seq dataset with clinical metadata^82^. F6: inflammatory myofibroblasts were significantly elevated in inflamed tissue, compared to non-inflamed tissue, and their prevalence correlated with clinical inflammation severity scores (Fig. 7e). Receptor–ligand analysis suggested that F6: inflammatory myofibroblasts recruit and maintain neutrophils (*CXCL5/6/8*-*CXCR2* and *CSF3*-*CSF3R*), macrophages/monocytes (*CCL5/26*-*CCR1* and *CSF3*-*CSF3R*) and B cells (*CXCL13*/*CXCR5*) in the skin (Fig. 7c and Methods). These genes were highly expressed in the skin during wound healing, acne and hidradenitis suppurativa, as well as in IBD and lung cancer (Fig. 7b), suggesting a similar mechanism for recruitment of immune cells across tissues.

Overall, our results suggest that F3: FRC-like (*CCL19*^+^*CD74*^+^*TNFRSF13B*^+^ and *IL33*/*IL15*) and F6: inflammatory myofibroblasts (*IL11*^+^*MMP1*^+^*CXCL5*^+^*IL7R*^+^) mediate distinct immune niches driving pathology in the skin and other tissues (Fig. 7f).

### F3: FRC-like fibroblasts in adult and prenatal human skin

**Fig. 8:**
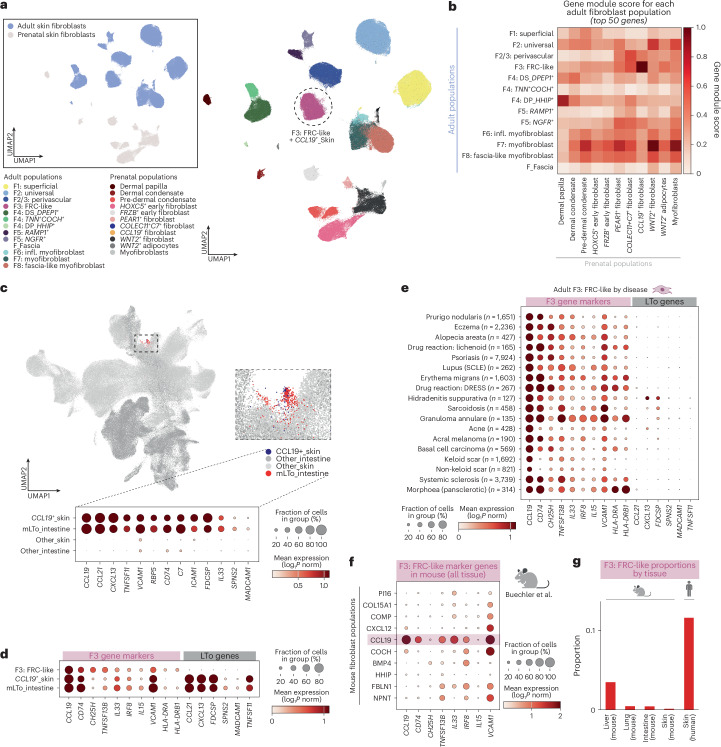
Adult skin F3: FRC-like fibroblasts potentially arise from prenatal skin LTo cells. **a**, UMAP visualization for prenatal human skin and adult skin colored by cell type and (insert) age group. **b**, Heatmap of adult skin gene module scores applied to prenatal skin fibroblasts. **c**, UMAP visualization for prenatal human skin and intestine. Insert: cluster of LTo-like cells. (m)LTo: (mesenchymal) lymphoid tissue organizer. Dotplot of marker gene expression for LTo-like cells by tissue. **d**, Dotplot of expression of F3: FRC-like fibroblasts and LTo-associated marker genes in healthy adult skin F3: FRC-like fibroblasts, prenatal skin CCL19^+^ fibroblasts and intestinal mLTo cells. **e**, Dotplot of expression of marker genes for adult skin F3: FRC-like fibroblasts and LTo-associated marker genes in diseased adult skin, by disease, for diseases with a minimum of 100 F3: FRC-like fibroblasts. DRESS, drug reaction with eosinophilia and systemic symptoms. **f**, Dotplot of expression of F3: FRC-like fibroblast marker genes in mouse steady-state cross-tissue atlas (Buechler et al.^4^). **g**, Proportion of Ccl19^+^ fibroblasts (labels from original study) in mouse in different healthy tissues (including mouse flank skin) and of F3: FRC-like fibroblasts in healthy human skin. Illustrations in **f** and **g** were partly created using BioRender.com.

Given the similar transcriptomic profiles of adult skin F3: FRC-like and intestinal T reticular cells, we hypothesized that these fibroblasts had similar origins in their respective tissues. Intestinal *FRCs* are found in the Peyer’s patch and are thought to arise from prenatal lymphoid tissue organizer (LTo) cells^67^; however, the skin does not harbor the equivalent of Peyer’s patch and the origin of F3: FRC-like cells remains unknown. To explore *F3* ontogeny from a developmental perspective, we first integrated adult and prenatal skin fibroblasts^66^ and identified the corresponding fibroblast populations (Supplementary Note 4). This identified that adult F3: FRC-like fibroblasts correlated with prenatal skin *CCL19*^*+*^ fibroblasts (Fig. 8a,b).

We next queried whether prenatal *CCL19*^*+*^ fibroblasts were equivalent to LTo-like cells by jointly integrating human prenatal skin and intestinal data^78^. Notably, prenatal skin *CCL19*^+^ cells and prenatal intestinal mesenchymal LTo cells clustered together (Fig. 8c). Prenatal skin *CCL19*^+^ cells expressed known mesenchymal LTo markers^78^, including *CCL21*, *CXCL13*, *MADCAM1*, *FDCSP* and *TNFSF11* (RANKL) (Fig. 8c), suggesting that prenatal skin *CCL19*^+^ cells may give rise to adult skin F3: FRC-like cells in a manner analogous to intestinal LTo cells.

We next investigated the LTo gene program in adult skin F3: FRC-like fibroblasts. The LTo gene program (including *CXCL13* and *FDCSP*) was not expressed in healthy adult skin but could be upregulated in specific skin diseases (Fig. 8d,e), particularly hidradenitis suppurativa. Tertiary lymphoid structures (TLS), for which *CXCL13* is an important chemokine^83^, are not well recognized in adult human skin, but have recently been reported in hidradenitis suppurativa specifically^83^, suggesting that the LTo gene program contributes to this process.

**Extended Data Fig. 10:**
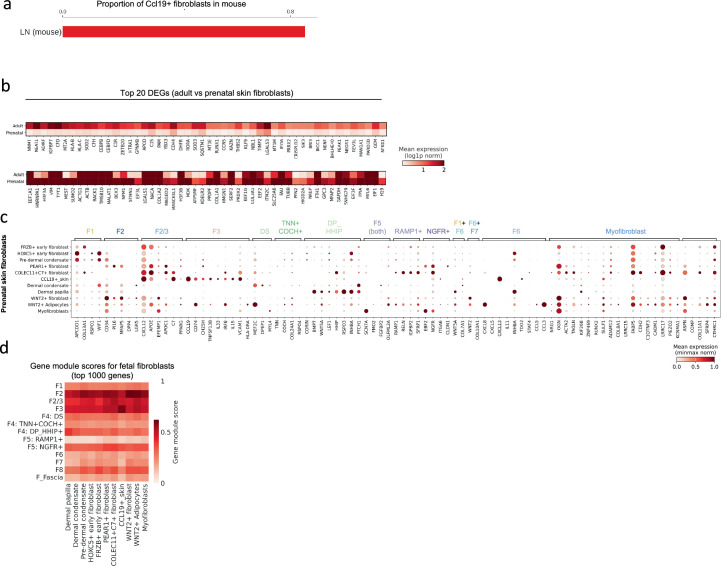
Prenatal and cross-species *F3* comparison. **a**) Proportion of fibroblasts in lymph node tissue that were annotated as the *Ccl19*+ subtype in mouse cross-tissue fibroblast atlas. **b**) Top 20 differential expressed genes (DEGs) for adult and prenatal skin fibroblasts. **c**) Dotplot of expression of adult skin fibroblast marker genes in prenatal skin. **d**) Heatmap of adult skin gene module scores applied to prenatal skin fibroblasts using top 1000 genes.

We next asked whether F3: FRC-like fibroblasts were unique to adult human skin, as LTo-like fibroblasts have not been reported in mouse embryonic skin^84^. Our comparative analysis showed that adult F3: FRC-like cells correspond to mouse *Ccl19*^+^ fibroblasts^4^ (Fig. 8f). Mouse *Ccl19*^+^ fibroblasts were found predominantly in lymphoid organs (Extended Data Fig. 10a), but were also present in other tissues such as lung (Fig. 8g), whereas F3: FRC-like fibroblasts were relatively abundant in healthy human skin, the equivalent *Ccl19*^+^ fibroblasts were notably rarer in healthy mouse skin (Fig. 8g). In summary, we suggest F3: FRC-like fibroblasts are enriched in human skin and not observed or absent in murine skin.

## Discussion

We harmonize skin fibroblast subtype nomenclature in health and disease, spatially resolve distinct fibroblast anatomical niches, and identify conserved fibroblast subtypes in human diseases affecting multiple tissues^5^.

FRCs are the paradigm of immunomodulatory fibroblasts^3^, maintaining discrete immune structures and facilitating specific immune cell interactions in lymphoid organs, with increasing evidence that they are present across human tissues^4,6^. Our data suggest that F3: FRC-like fibroblasts are located in the superficial perivascular niche in human skin and have an analogous role to T zone reticular FRCs, mediating T-DC interactions. In keeping with previous murine studies^85^, we find that F3: FRC-like fibroblasts show a uniquely high prevalence in human skin. This enrichment of F3: FRC-like fibroblasts in human skin would explain both the absence of fibroblasts in murine skin TLS-like structures^86^ and why the prominent perivascular infiltrate structures that characterize many human inflammatory skin diseases are not reported in mice^85^.

Inflammatory myofibroblasts were recently reported in a large-scale integration of predominantly CAFs^5^ and have been independently described in IBD^78,80,81^. We identify that the same inflammatory myofibroblast phenotype (*IL11*^+^*MMP1*^+^*CXCL8*^+^*IL7R*^+^) can be observed in early human skin wounds, skin cancer and inflammatory skin diseases with scarring risk. Consistent with the immune milieu in early wounds, acne and hidradenitis suppurativa, we suggest that these fibroblasts recruit immune cells such as neutrophils and monocytes. Neutrophils are also reported to be recruited by inflammatory myofibroblasts in IBD^81^. Our study also suggests that F6: inflammatory myofibroblasts are an intermediate myofibroblast differentiation state in human skin, which agrees with recent work in mouse skin^53^ and lung^74^. Further work validating myofibroblast trajectories in human skin is needed as current trajectory inference methods are limited for predicting multiple cell states converging to a final phenotype, and lineage plasticity is likely in fibroblasts, with both tissue-specific and universal populations suggested to give rise to myofibroblasts^87^. Notably, skin could serve as an exemplar tissue to further investigate myofibroblast development in humans in vivo given the ability to sample tissue temporally with low morbidity.

A limitation of our study is that we relied on the uncertainty mechanism incorporated in scPoli to identify disease-associated populations. Our cross-tissue analysis using semi-supervised integration may underestimate tissue-specific differences between fibroblasts, and further investigation using methods such as contrastive analysis may be valuable. LTo-like cells in prenatal skin were rare and more definitive lineage tracing methods are required to understand prenatal to adult fibroblast transitions.

In summary, our annotated skin fibroblast dataset of 357,276 cells provides a foundational resource for fibroblast transcriptomic states in health and across distinct disease categories in skin tissue. Further comments on annotation can be made via the centralized community annotation platform (https://celltype.info/project/388).

## Methods

### Single cell: atlas data preprocessing, integration and selecting fibroblasts

Raw scRNA-seq data were downloaded and aligned using STARsolo (GRCh38-2020-A reference) unless already available in local storage^29,40^. We included publicly available data generated from fresh skin biopsies using the 10x Genomics Chromium platform. We collected essential metadata (sample ID, dataset ID, site status, patient status, sex and anatomic location) as more extensive metadata are being collected as part of the Human Skin Cell Atlas.

CellBender v.0.3 was used to correct for ambient messenger RNA^88^. To remove low-quality cells, we included only cells with >200 genes, >1,000 and <300,000 total unique molecular identifiers and a mitochondrial gene percentage of <15%. We calculated doublet scores using scrublet on a per sample basis using scrublet and removed cells with a doublet score of >0.3 (ref. ^89^).

Integration of all cells was performed using scVI using raw counts^48^. For feature (highly variable gene (HVG)) selection, we did not consider the following genes: mitochondrial genes; cell cycle genes, from https://github.com/haniffalab/skin_fibroblast_atlas/blob/main/misc/cc_genes.csv); hypoxic genes; and ribosomal genes. We selected 6,000 HVGs as features, and batch-aware HVG selection was performed by setting the batch key to sample ID. In future analyses post-integration, all genes were considered.

The following hyperparameters were used for the scVI model: number of layers: 2; number of latent dimensions: 30; gene likelihood: zero-inflated negative binomial distribution, and dispersion: gene-batch. An early stopping patience of five epochs was used. The batch key was ‘sampleID’ and no other covariates were passed to the model for correction.

We constructed a *k*-nearest neighbors (*k*-NN) graph (*k* = 30) using the scVI embedding and performed community detection (Leiden algorithm) with resolution 0.1 for the dataset with all skin cells. Visualization in two dimensions was performed using UMAP with the initialized positions from PAGA implemented in scanpy^90,91^. We then selected the fibroblast cluster for further analysis based on canonical marker gene expression, including *PDGFRA*, *DCN* and *LUM*) (Extended Data Fig. 1a). We did not include a distinct stress response cluster (*MT2A*, *MT1M*, *MT1X*, *HSP90AA1*, *JUNB*, *GADD45B* and *IER3*) as this population was not evident on Xenium analysis and thus likely related to cell dissociation. For a sensitivity analysis with additional cell types, we also selected Schwann and pericyte clusters.

### Single-cell analysis: fibroblast-only integration in health

We repeated integration using scVI for healthy and phenotypically normal (nonlesional) skin fibroblasts only. We used the same workflow as above. Visualization in two dimensions was performed using UMAP with positions initialized from PAGA.

We show gene expression values post-normalization. Normalization is a two-step procedure involving depth normalization and variance stabilization. We used the shifted logarithm with a scaling factor of 10,000 based on strong performance in a recent benchmarking paper^92^.

### Single-cell analysis: fibroblast cluster annotations in health

For each cluster, we calculated differentially expressed genes (DEGs) using the *t*-test with the scanpy rank_genes_groups function. The top DEGs for each population are shown in Supplementary Fig. 1a and Supplementary Table 2. For selecting marker genes to present for each population in Fig. 1c, we selected genes with the highest specificity of expression for that cluster from visualization.

We selected our nomenclature for fibroblasts based on our previous report of F1–F3 fibroblasts^40^. We refined the names based on spatial location and our in-depth characterization of cell states, including across tissues. We renamed F1 as F1: superficial, F2 to F2: universal and F3 to F3: FRC-like. In this work, we defined novel fibroblast subtypes. F2/3 fibroblasts expressed select F2 (*CD34* and *PI16*) and F3 markers (*CXCL12*, *PLA2G2A* and *C7*), but additionally expressed *PPARG*. F2/3 fibroblasts also showed a distinct location enriched in both superficial and deep dermal perivascular structures, compared to F2 (deep dermis but not around vasculature) and F3 (superficial perivascular regions) (Extended Data Fig. 4c). We therefore named this population F2/F3: Perivascular. We additionally identified novel F4 and F5 populations. We grouped three subtypes as F4 based on transcriptomic (*ASPN*/*COL11A1*) and spatial similarity (localization around the hair follicle). We grouped two subtypes as F5 based on shared expression of genes also expressed by Schwann cells, including *SCN7A*, *FMO2* and *OFLML2A*, and spatial association with nerve structures.

We took two approaches to minimize the possibility of missing a rare distinct cluster. First, we repeated unsupervised clustering at a higher resolution and assessed whether any clusters showed distinct gene expression. Second, we reviewed reported marker genes for fibroblast clusters in previously reported scRNA-seq data for human skin to identify if a novel distinct population has been reported.

### Single-cell analysis: transcription factor activity inference

Transcription factor activity inference was performed using decoupler^51^, using the get_collectri (human) and run_ulm functions. This analysis was performed using the data subset of 6,000 HVGs.

### Single-cell analysis: gene set enrichment analysis

Gene set enrichment analysis was implemented using GSEAPY^93^. We used the top 500 DEGs per fibroblast subtype and the GO_Biological_Process_2023 gene set. A cutoff statistical significance of 0.01 was used.

### Spatial transcriptomics: Visium data generation

We generated new spot-based spatial transcriptomic data using the 10x Genomics Visium Spatial Gene Expression platform from frozen OCT-embedded human adult skin tissue (*n* = 1 lesional atopic dermatitis and *n* = 2 nonlesional atopic dermatitis). All research ethics committee and regulatory approvals were in place for the collection of research samples at Newcastle and for their storage at the Newcastle Dermatology Biobank (REC reference no. 19/NE/0004). Skin samples were sectioned at 15-µm thickness and the optimal tissue permeabilization time was determined as 14 min. H&E images were taken using a Zeiss AxioImager with apotome microscope (Carl Zeiss Microscopy) and Brightfield imaging (Zeiss Axiocam 105 48-color camera module) at ×20 magnification. The ZEN blue edition v.3.1 (Carl Zeiss Microscopy) software was used to acquire the H&E images following z-plane and light balance adjustment and image tile stitching. Spatial gene expression libraries were sequenced using an Illumina NovaSeq 6000 to achieve a minimum number of 50,000 read pairs per tissue covered spot.

### Spatial transcriptomics: Visium deconvolution

The 10x Genomics Visium data were mapped using Spaceranger v.1.3.0 using GRCh38-2020-A reference. Visium provides whole transcriptome coverage over a 55-μm diameter spot area. We therefore used the cell2location (v.0.1.3) to deconvolute the cell types predicted to be present in a given spot^94^. We constructed a reference signature using sample as batch_key. We included our fibroblast atlas and other skin cell types from Reynolds et al.^40^. Then we performed deconvolution with the following parameters: detection_alpha = 20,N_cells_per_location = 30 andmax_epochs 50_000. Values of all other parameters were kept default. Following the cell2location tutorial, we used 5% quantile of posterior distribution (q05_cell_abundance_w_sf) as predicted cell-type abundances.

### Spatial transcriptomics: Xenium data generation

Nonlesional (*n* = 2; one sample nonlesional pre-treatment, one sample nonlesional post-treatment) and lesional (*n* = 1; inflamed) atopic dermatitis human adult skin tissue was used to generate in situ gene expression data using the 10x Genomics Xenium in situ 5k-plex platform. All research ethics committees and regulatory approvals were in place for the collection and storage of research samples at St John’s Institute of Dermatology, Guy’s Hospital, London (REC reference no. EC00/128).

### Spatial transcriptomics: Xenium data preprocessing and integration

The 10x Genomics Xenium data were filtered to exclude cells with <10 genes per cell. Integration of all cells was performed using scVI using raw counts^48^. Batch-aware HVG selection was performed by setting the batch key to sample ID. We selected 2,000 HVGs as features for the integration. As Xenium data are more sparse than scRNA data, we used a simpler encoder (number of layers: 1; number of latent dimensions: 10) than scRNA. The batch key was ‘sampleID’ and no other covariates were passed to the model for correction.

For normalization, we again used the shifted log transformation for count normalization. We constructed a *k*-NN graph (*k* = 20, given the smaller dataset size) using the scVI embedding. We performed community detection (Leiden algorithm) with resolution 0.1 and used the same markers as scRNA data to select the fibroblast cluster.

We used marker genes from scRNA-seq data to label fibroblast populations through manual annotation. Cell coordinates colored by cell type were visualized using squidpy (sq.pl.spatial_scatter)^95^.

We used the same integration strategy for newly generated atopic dermatitis data and publicly available cutaneous melanoma data. Cutaneous melanoma data were downloaded from https://www.10xgenomics.com/datasets/xenium-prime-ffpe-human-skin.

### Spatial transcriptomics: Xenium niche identification

NicheCompass was run after selecting 1,024 spatially variable genes. The number of neighbors selected per cell was 8, and otherwise default settings were used^79^.

### Spatial transcriptomics: Xenium neighborhood enrichment

To calculate neighborhood enrichment scores, we first constructed an adjacency matrix for indicating which cells were connected using the spatial_neighbors functions in squidpy. We determined the neighborhood as a cell as cells within 20 µm of an index cell. We then applied neighborhood enrichment analysis (nhood_enrichment function in squidpy) to quantify which cell types were more frequently colocalized than expected by chance. Heatmaps of enrichment scores were visualized using sq.pl.nhood_enrichment.

### Single-cell analysis: fibroblast-only integration in disease

To identify fibroblast clusters in disease, we mapped lesional data to the healthy/nonlesional reference described above. We used a state-of-the-art deep meta-learning model (scPoli^61^). This approach first trains a model using the healthy/nonlesional (reference) to generate centroids (Fig. 3a). Then, cells in the query that are distinct to centroids generated from the reference are marked as uncertain, which facilitates the discovery of new cell types/states in the query data (Fig. 3a). This permits automated cell annotation while highlighting cells that could not be mapped to the reference through prototypical learning^61^.

Due to the proposed role of hypoxia in myofibroblast differentiation^53^, we included hypoxic genes for consideration in feature selection. To ensure this selection did not bias results, we repeated an scVI integration using the same methodology as for healthy fibroblasts (Supplementary Fig. 4a,b).

### Single-cell analysis: fibroblast cluster annotations in disease

Our definition of ‘uncertain’ cells was derived from scPoli. scPoli utilizes Euclidean distances of query cells from prototypes (or centroids) generated from the reference to yield an uncertainty associated with each cell. To manually annotate cells labeled as uncertain, we calculated DEGs for each Leiden cluster and also assessed expression of healthy fibroblast marker genes in each population. The top DEGs for each cluster considered as disease-associated or disease-specific are shown in Supplementary Fig. 1c and Supplementary Table 3. We also considered which populations were enriched in disease.

### Validation of fibroblast cluster annotations

To validate our reported fibroblast subtypes, we used two approaches. First, we used skin scRNA-seq datasets not used in the original integration^96^. Using the scPoli model to generate embeddings and transfer labels to these populations, we identified expected populations from earlier analysis (Supplementary Fig. 4c). Second, we used Xenium data to validate the existence of the same clusters in which cell gene expression profiles are generated in situ, without tissue dissociation.

#### Single-cell analysis: CellDISECT

For CellDISECT, we used 6,000 HVGs and raw counts as input. Model architecture and training schedule is listed in the provided code.

#### Single-cell analysis: PROGENy analysis

We used the top 500 human genes per pathway and default settings for the multivariate linear model (decoupler.run_mlm)^97^.

#### Single-cell analysis: clinical classification of scarring risk and proportions by category

We broadly grouped individual diseases into disease categories (inflammatory + low scarring risk, inflammatory + high scarring risk, cancer, established scarring/fibrosis) based on clinical disease features. Of note, clear separation of diseases is not possible because of a well-recognized link between inflammation and fibrosis^98^. For example, an inflammatory component to systemic sclerosis is well recognized and first-line treatments typically include immunosuppressants, but this disease also features established fibrosis. Additionally, we included sarcoidosis and granuloma annulare (both disorders of granulomatous inflammation) in ‘inflammatory high scarring risk’ as fibrosis can occur within granulomas^99^. Additionally, pulmonary sarcoidosis is a well-recognized cause of pulmonary fibrosis; however, most cases of cutaneous sarcoidosis and granuloma annulare do not scar. Prurigo nodularis was classified as low scarring risk as it is unclear whether scarring arises secondary to scratching. We considered cancer as moderate scarring risk as fibrosis can occur within lesions (desmoplasia)^100^ and self-resolving melanoma can result in scarring^101^.

For fibroblast proportions by scarring category, we calculated the s.e.m. for each category using the mean proportion for each donor. The s.e.m. for each disease category was derived from the s.d. of donor-level proportions divided by the square root of the number of donors in that category.

#### Immunofluorescence validation (LRRC15 and ADAM12)

All research ethics committees and regulatory approvals were in place for the collection and storage of atopic dermatitis skin samples at the St John’s Institute of Dermatology, Guy’s Hospital, London (REC reference no. EC00/128) and hidradenitis suppurativa skin samples at Newcastle Dermatology Biobank (REC reference no. 19/NE/0004). Fresh-frozen OCT-embedded skin samples were sectioned at 10-µm thickness directly onto superfrost microscope slides and stored at −80 °C. Slides were air dried at room temperature for 10 min and then fixed using 4% PFA for 10 min. Next, a blocking solution of 5% normal goat serum with 0.01% Triton X-100 was applied to the tissue sections and incubated for 1 h at room temperature. Slides were then incubated with primary antibodies overnight at 4 °C. The next day, slides were washed with 1× PBS and incubated with secondary antibodies for 1 h at room temperature. Then, 4,6-diamidino-2-phenylindole (DAPI) was used to demarcate nuclei and slides were mounted with DAKO mounting medium before applying coverslips and leaving slides to dry overnight. Skin sections were imaged using a Leica SP8 confocal microscope.

#### Gene module scoring

To calculate gene scores, we used the score_genes() function in scanpy. We used arguments of ctrl_size = 1,000 and n_bins = 25. We used the marker genes reported for each population, rather than more extensive gene lists, based on the rationale that larger gene programs would be more likely to include tissue-specific gene expression and thus underestimate transcriptomic similarity across tissues.

#### Single cell: predictive modeling of stromal subtype composition

We trained a random forest classifier using fibroblast subtype composition as input to predict scarring risk group. We evaluated performance in terms of average F1 score, a widely used metric for evaluating classification performance^102^, computed using classification_report from scikit-learn. We applied fivefold stratified cross-validation to train and evaluate a RandomForestClassifier (100 estimators, random_state = 42). To identify cell types that are most predictive of scarring status, we extracted the final trained model’s feature importances.

#### Single cell: trajectory inference

For trajectory inference, we used both velocity-based (RNA velocity (scVelo and CellRank2 Velocity kernel))^103,104^ and graph-based (PAGA and Monocle 3)^90,105^ approaches. We used data for which we could calculate RNA velocity using velocyto and scvelo^104,106^.

We first used PAGA (as implemented in scanpy)^90^, plotted using a threshold of 0.1 and applied to the whole dataset. In future analyses, we excluded F5: Schwann-like fibroblasts, which seemed to be distinct (Extended Data Fig. 8a) and F_Fascia, which were observed in few diseases (Fig. 4a). As healing and scarring is observed on non-hair-bearing sites, we also did not include F4: hair follicle-associated fibroblasts. We generated new scVI embeddings for the lesional fibroblasts and re-calculated the *k*-NN graph using the top 2,000 HVGs, followed by UMAP visualization. Velocity pseudotime was calculated using scvelo^104^. We re-calculated the PAGA plot using only lesional fibroblasts, again using a threshold of 0.1.

For Monocle 3 (v.1.3.7)^105^, the expression count matrix along with the corresponding cell and gene metadata from the processed anndata object in scanpy was used to create Monocle object (cell_data_set object). The cell_data_set object was then pre-processed using default settings and aligned to correct for batch effects based on the ‘dataset_id’. Dimensionality reduction was performed using ‘UMAP’ as the reduction method. Cells were clustered with a resolution of 1 × 10 − 6 and ‘UMAP’ as the reduction method. A trajectory graph was learned by adjusting parameters such as geodesic distance ratio (0.5) and minimal branch length (10) to optimize for large datasets. Finally, cells were arranged in pseudotime by manually selecting root nodes from the F2: universal population. We used F2: universal as the root state in Monocle 3 based on velocity pseudotime results and previous work^4,55^. The ordered and learned graph object was then used to plot the pseudotime trajectory plots. The RNA velocity kernel was calculated using CellRank2.

#### Single cell: wound integration

We obtained post-alignment skin wound data from the authors of ref. ^75^ and processed these using CellBender as previously described for skin fibroblast data. To annotate skin wound fibroblasts, we integrated the unlabeled skin wound cells with our labeled integrated skin dataset using scanVI. We used the same model architecture (30 latent dimensions, two layers) and the same number of input HVGs (*n* = 6,000). We selected only fibroblasts for further analysis, using the same downstream strategy as used for skin fibroblasts previously.

#### Single cell: cross-tissue fibroblast state comparisons and integration

For cross-tissue marker gene comparisons, we selected reported populations from Korsunsky et al., Buecher et al. and Gao et al.^4–6^ As Gao et al. reported six universal and five shared populations, we show matching populations in the main figure and other populations in the extended figure.

For cross-tissue integration, we concatenated our labeled skin data with other tissues (raw count data). HLCA data^8^, gut atlas data^78^ and Human Endometrial Cell Atlas data^107^ were available locally. We downloaded nasal tissue data from https://ngdc.cncb.ac.cn/gsa-human/browse/HRA000772 (ref. ^108^), heart data from https://data.humancellatlas.org/explore/projects/e9f36305-d857-44a3-93f0-df4e6007dc97, rheumatoid arthritis from https://www.immport.org/shared/study/SDY998 (ref. ^109^) and additional intestinal data from the Gene Expression Omnibus (GEO) under accession code GSE282122. We used the same number of input HVGs (*n* = 6,000).

We integrated data in a semi-supervised manner using scANVI^110^, where skin cell types were labeled and cells from other tissues were unlabeled. We used the same scANVI hyperparameters as for wound data but with a smaller number of maximum epochs (*n* = 10) due to the larger dataset size. We then calculated *k*-NN (*k* = 30) and performed low-resolution Leiden clustering (resolution 0.1). We selected a fibroblast cluster based on canonical marker genes, which also contained the labeled skin fibroblasts.

To annotate clusters, we labeled clusters by the majority skin fibroblast population (Fig. 6c and Extended Data Fig. 8b) (for example if F3: FRC-like was the predominant skin fibroblast subtype, we labeled the cluster as F3: FRC-like*)*. We then assessed gene expression markers for each cluster, excluding skin fibroblasts, to ensure that skin fibroblasts did not drive the gene expression signature for that cluster. We also plotted gene expression for each cluster by tissue using our previously reported marker genes for each cluster.

To assess F3: FRC-like fibroblasts in HLCA data, we performed the same clustering strategy as previously described for skin fibroblasts and then plotted F3: FRC-like marker genes by cluster.

#### Single cell: association of IBD F6: inflammatory myofibroblasts with clinical severity

Inflammation severity scores were obtained from GEO under accession code GSE282122. A linear regression model was fitted using ordinary least squares with the F6 proportion as the dependent variable and inflammation severity score as the independent variable using seaborn’s regplot function.

#### Single cell: cell–cell interactions

We used CellPhoneDB v.5 (method 2) for cell–cell communication analysis^111^. We combined our fibroblast data with skin immune cells from our previously published scRNA-seq data from skin with more granular immune cell annotations (Reynold*s* et al.)^40^. We restricted interactions to marker genes for F3: FRC-like fibroblasts and F6: inflammatory myofibroblasts. We visualized the results using ktplotspy.

#### Single cell: prenatal skin-intestine and adult skin comparison

For prenatal skin and prenatal intestine, we concatenated raw count adata objects from intestine^78^ with our prenatal skin data from a previous publication^66^. We ran scVI using the same parameters as for the healthy/nonlesional integration. We used the same strategy for the adult skin and prenatal skin integration.

#### Single cell: mouse FRC-like/Ccl19 comparison

For mouse comparisons, we downloaded the mouse steady-state atlas from https://www.fibroxplorer.com/download. We loaded the data as an adata object using pandas2ri in rpy2. We used labels for *Ccl19*^+^ fibroblast from the original study.

### Reporting summary

Further information on research design is available in the Nature Portfolio Reporting Summary linked to this article.

## Online content

Any methods, additional references, Nature Portfolio reporting summaries, source data, extended data, supplementary information, acknowledgements, peer review information; details of author contributions and competing interests; and statements of data and code availability are available at 10.1038/s41590-025-02267-8.

## Supplementary information

Supplementary Information Supplementary Figs. 1–4, Tables 1–3 and Notes.

Reporting Summary

## Data Availability

Newly generated sequencing data (Visium) for inflamed atopic dermatitis from this study are available from the European Genome-phenome Archive at EGAS00001006482 (sample EGAN00004379723). Healthy Visium data are available from Array Express at E-MTAB-15458. Xenium data are available from https://www.ebi.ac.uk/biostudies/bioimages/studies/S-BIAD2214. Our processed data can be downloaded and explored on an online webportal at https://cellatlas.io/studies/skin-fibroblast. Publicly available scRNA-seq data and access links are shown in Supplementary Table 1.

## Code availability

The code generated during this study is available at GitHub at https://github.com/haniffalab/skin_fibroblast_atlas.
