# Supplementary material for: A single-cell and spatial genomics atlas of human skin fibroblasts reveals shared disease-related fibroblast subtypes across tissues
Source: Nat Immunol. 2025 Sep 24;26(10):1807–20. doi: 10.1038/s41590-025-02267-8 (PMC12479362; doi:10.1038/s41590-025-02267-8)
Supplement: Supplementary file 1 — Supplementary Figs. 1–4, Tables 1–3 and Notes. [file 41590_2025_2267_MOESM1_ESM.pdf]

nature immunology

Supplementary information

https://doi.org/10.1038/s41590-025-02267-8

A single-cell and spatial genomics atlas of human skin fibroblasts reveals shared disease-related fibroblast subtypes across tissues

In the format provided by the authors and unedited

Supplementary Fig. 1

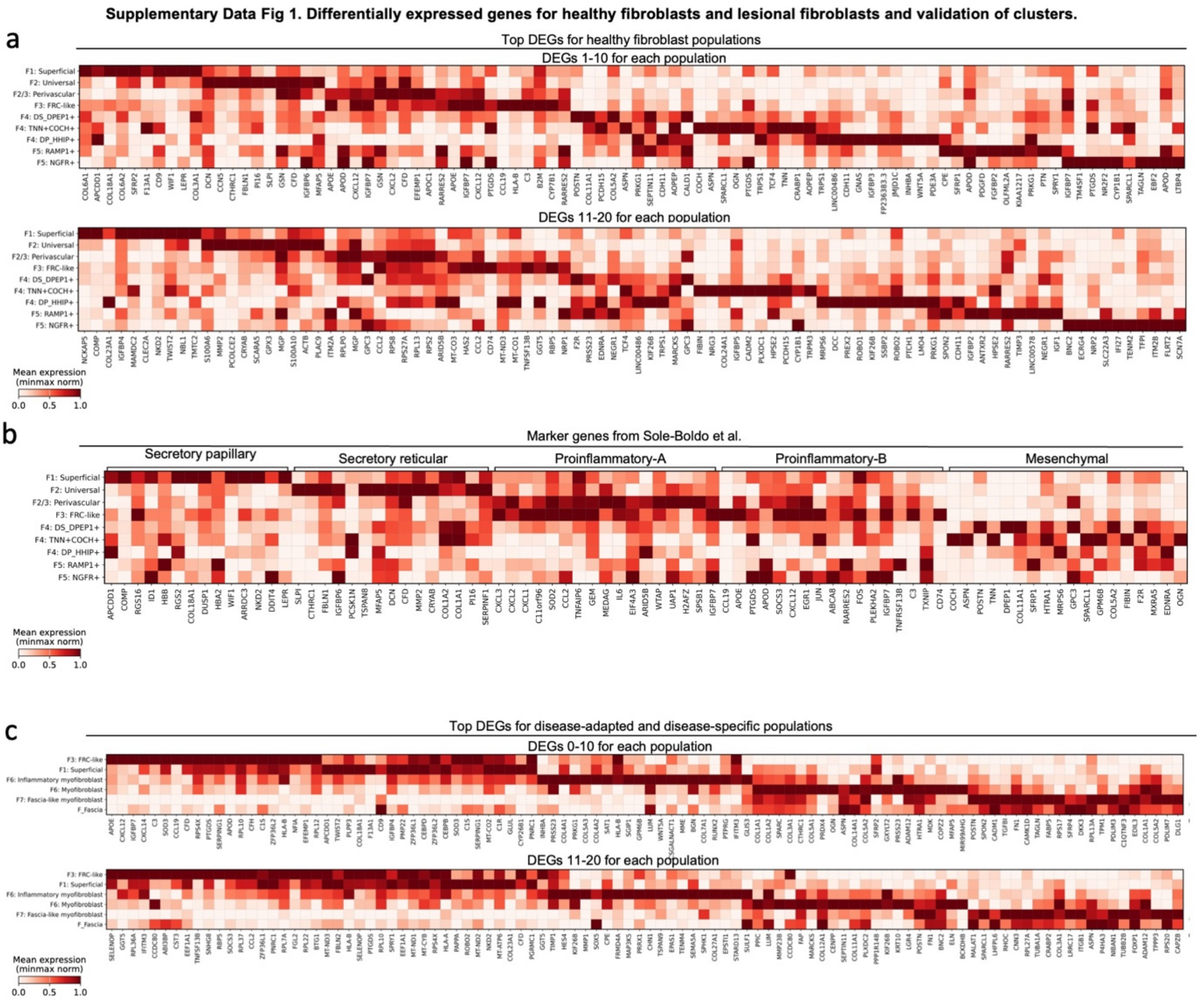

Supplementary Data Fig. 1 | Differentially expressed genes for fibroblasts and skin-specific nomenclature and validation of clusters. a) Heatmap of top differentially expressed genes (DEGs) for each fibroblast population. Top row: Top 10 DEGs. Bottom row: 11-20 top DEGs. b) Heatmap of expression of marker genes reported by Sole-Boldo *et al*. c) Heatmap of top differentially expressed genes (DEGs) for disease-specific and disease-adapted populations. Top row: Top 20 DEGs. Middle row: 20-40 top DEGs. Bottom row: 40-60 top DEGs.

Supplementary Fig. 2

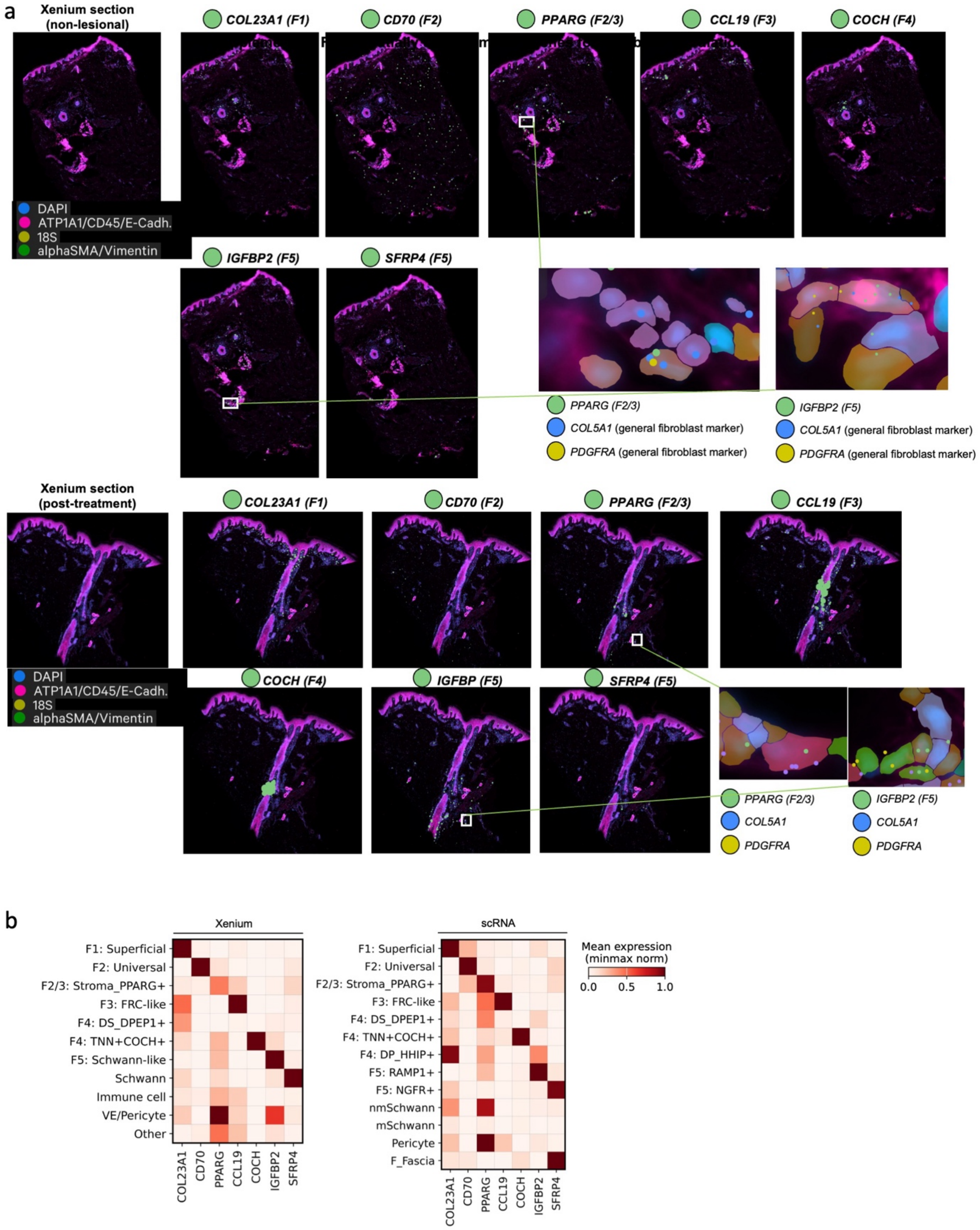

Supplementary Data Fig. 2 | Spatially distinct marker for fibroblast populations. a) Expression of fibroblast markers in non-inflamed skin from Xenium explorer. Zoomed in panels show marker genes co-occurring with general fibroblast markers for *F2/3* and *F5* marker genes. b) Heatmap of expression of marker genes used in panel a in Xenium and scRNA data.

Supplementary Fig. 3

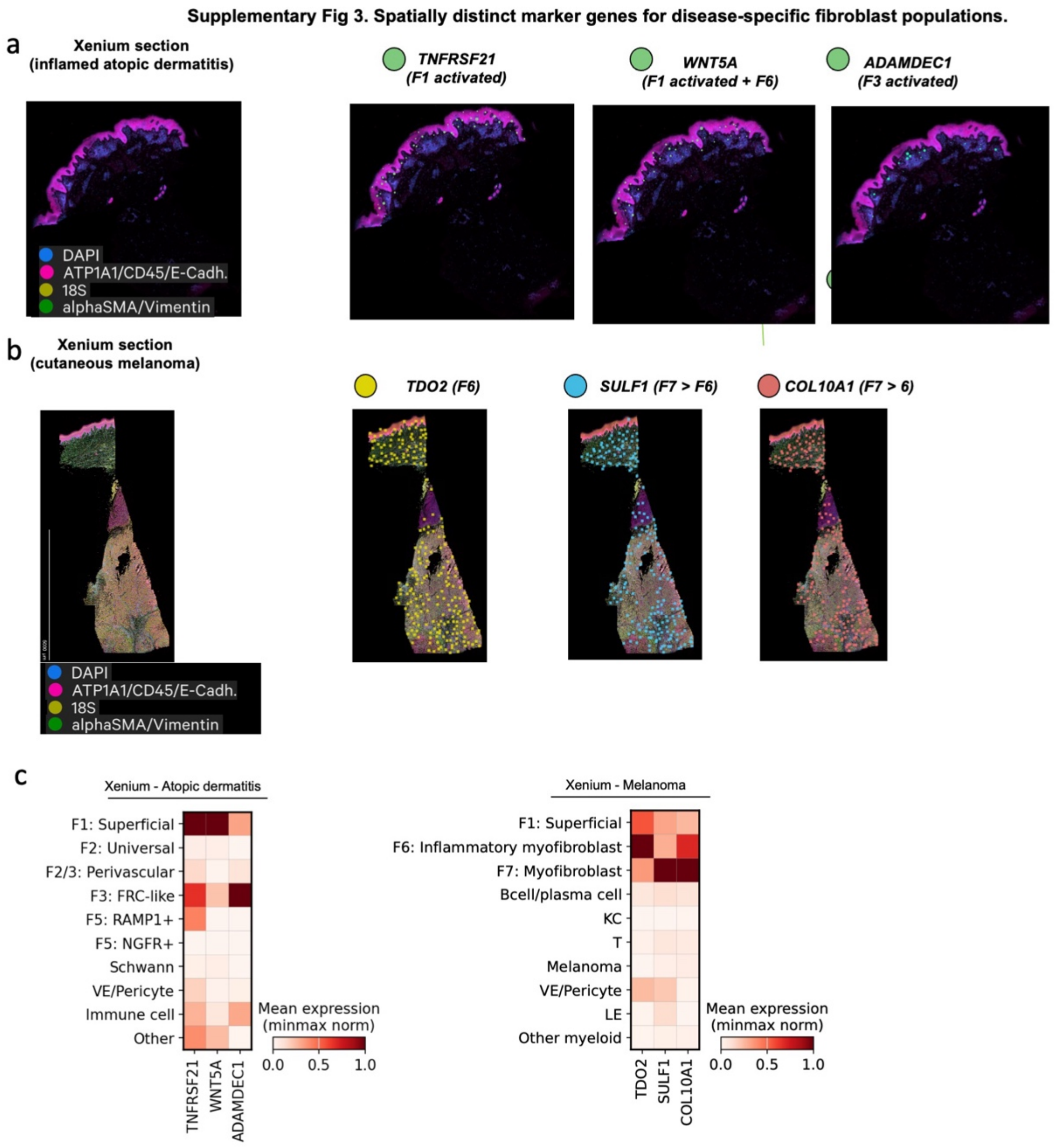

Supplementary Fig. 3 | Spatially distinct marker genes for disease-specific fibroblast populations. a) Expression of activation markers of F1 and F3 disease-adapted fibroblasts in inflamed atopic dermatitis skin. b) Expression of F6 and F6/7 markers in cutaneous melanoma. c) Dot plot of gene expression in fibroblasts and other cell types in atopic dermatitis (inflamed) and cutaneous melanoma.

Supplementary Fig. 4

Supplementary Figure 4. Sensitivity analysis integration and external mapping of unseen dataset.

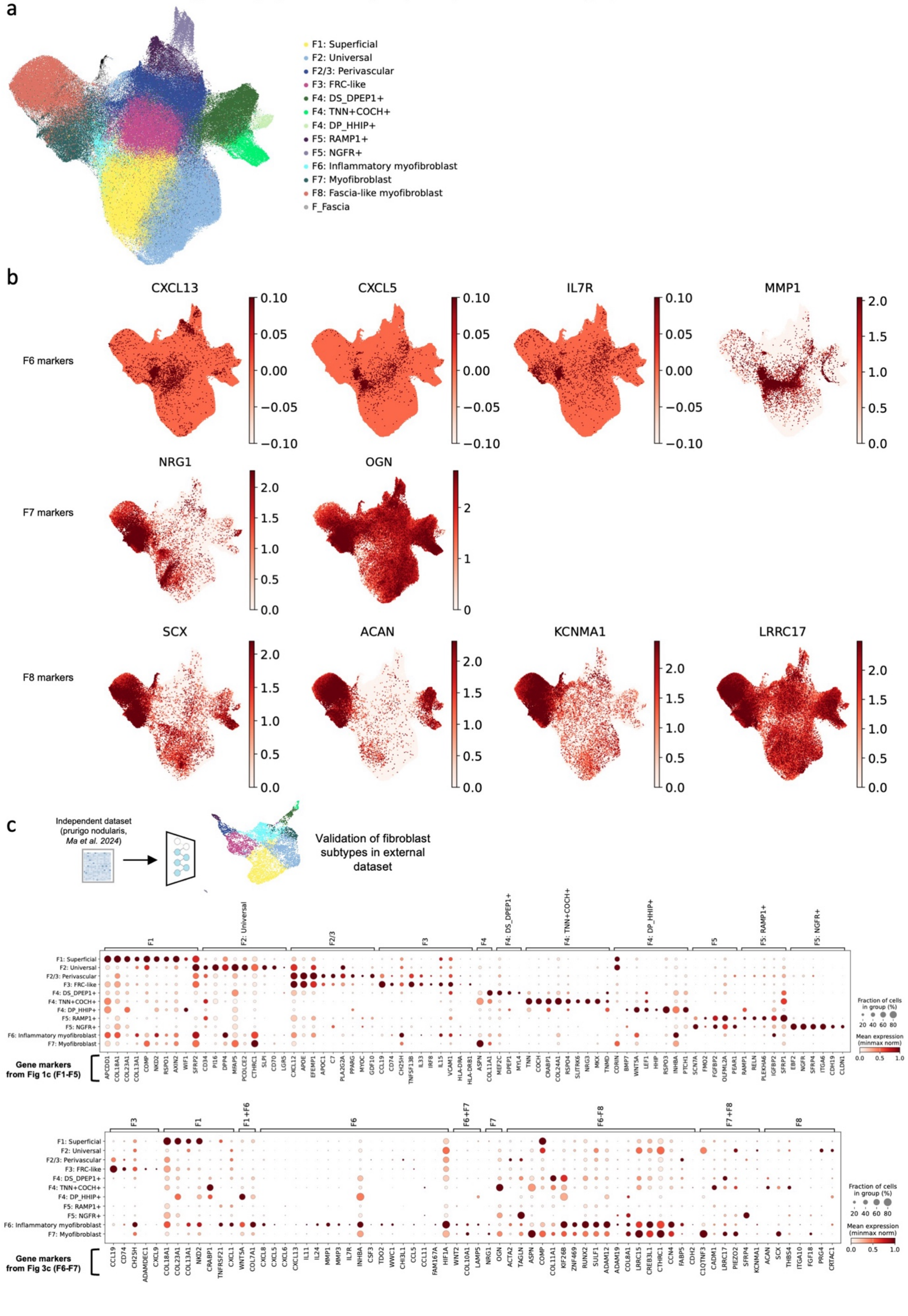

Supplementary Fig. 4 | Sensitivity analysis integration and external mapping of unseen dataset. a) Integration of all fibroblasts with scVI with inclusion of hypoxic genes. b) UMAP visualisation of marker genes for disease-specific fibroblast clusters (*F6*, *F7, F8*). c) Dotplot of a marker genes for healthy and disease fibroblast subtypes in an external dataset (prurigo nodularis with non-inflamed and inflamed samples).

Supplementary Table 1

GSE or other link	First author	Pubmed ID (PMID)	Year of publication	Diseases included
GSE202352	Wiedemann	36732947	2023	
GSE228421	Francis	38291032	2024	Psoriasis
GSE181316	Direder	36333467	2022	Keloid
GSE173252	Dobie	34274346	2022	Dupuytren contracture
GSE173706	Ma	38051587	2024	Psoriasis
GSE138669	Tabib	34042322	2022	Systemic sclerosis
GSE144236	Ji	32579974	2020	Squamous cell carcinoma
GSE186476	Dunlap	35290245	2022	Lupus
GSE249279	Ma	38172207	2024	Systemic sclerosis
PRJNA754272	Ahlers	35069694	2022	
GSE212450	Ober-Reybolds	37500727	2023	Alopecia areata
GSE175990	Mariottoni	34504848	2021	Hidradenitis suppurativa
GSE158924	Wang	33317858	2021	Granuloma annulare
GSE166796	Jiang	34061047	2021	Lyme disease (erythema migrans)
https://spatial-skin-atlas.cellgeni.sanger.ac.uk/	Ganier	38165934	2024	Basal cell carcinoma
GSE130973	Sole-Boldo	32327715	2020	
https://developmentcellatlas.ncl.ac.uk/datasets/hca_skin_portal.	Reynolds	33479125	2021	Atopic dermatitis, psoriasis
GSE229279	Damo	37344588	2023	Immunotherapy reaction (lichenoid cutaneous)
GSE132802	Kim	31959990	2020	DRESS
GSE175817	Do	35171653	2022	Acne
GSE181297	Shim	35123990	2022	Keloid
GSE215120	Zhang	36433984	2022	Acral+cutaneous melanoma
GSE123813	Yost	31359002	2019	Basal cell carcinoma and squamous cell carcinoma
GSE163028	Neurofibroma	33413690	2021	Neurofibroma
GSE169147	Damsky	35668129	2022	Sarcoidosis
GSE234987	Xing	37471168	2023	Pansclerotic morphoea
GSE163973	Deng	34140509	2021	Keloid
GSE233280	Patel	38246584	2024	Prurigo nodularis
GSE154775	Gudjonsson	32853177	2020	Hidradenitis suppurativa
PRJNA607098	Layton	32488016	2020	Dupuytren contracture
GSE189889	Li	35247927	2022	Acral melanoma
GSE181907	Yerly	35986012	2022	Basal cell carcinoma

Supplementary Table 1 | scRNA datasets used.

Supplementary Table 2

Supplementary Table 2 | Differentially expressed genes for healthy fibroblasts.

SupplementaryTable2 (1)

Gene	logFC	p-value	Group
**COL6A1**	2.028954	0E+00	F1: Superficial
**APCDD1**	3.7028298	0E+00	F1: Superficial
**COL18A1**	3.613811	0E+00	F1: Superficial
**COL6A2**	1.2061229	0E+00	F1: Superficial
**SFRP2**	2.1225624	0E+00	F1: Superficial
**F13A1**	3.2275982	0E+00	F1: Superficial
**CD9**	1.4832735	0E+00	F1: Superficial
**WIF1**	3.1227763	0E+00	F1: Superficial
**LEPR**	2.6276667	0E+00	F1: Superficial
**COL3A1**	1.6713915	0E+00	F1: Superficial
**NCKAP5**	2.6882675	0E+00	F1: Superficial
**COMP**	3.2188938	0E+00	F1: Superficial
**COL23A1**	4.017613	0E+00	F1: Superficial
**IGFBP4**	1.652801	0E+00	F1: Superficial
**MAMDC2**	2.1375833	0E+00	F1: Superficial
**CLEC2A**	4.3528147	0E+00	F1: Superficial
**NKD2**	3.8156025	0E+00	F1: Superficial
**TWIST2**	1.3576341	0E+00	F1: Superficial
**NBL1**	1.3843836	0E+00	F1: Superficial
**TMTC2**	2.207969	0E+00	F1: Superficial
**TCF4**	1.3994658	0E+00	F1: Superficial
**GREM2**	2.4612331	0E+00	F1: Superficial
**HSPB3**	4.411438	0E+00	F1: Superficial
**DUSP1**	1.2579638	0E+00	F1: Superficial
**C1orf198**	1.9880133	0E+00	F1: Superficial
**COL1A1**	1.3151307	0E+00	F1: Superficial
**TGFBI**	2.3641925	0E+00	F1: Superficial
**COL5A1**	1.5478323	0E+00	F1: Superficial
**CAV1**	1.1047987	0E+00	F1: Superficial
**ANTXR1**	1.4605635	0E+00	F1: Superficial
**SPARC**	1.1155849	0E+00	F1: Superficial
**DKK 3.00**	1.9081341	0E+00	F1: Superficial

**PTGDS**	2.054385	0E+00	F1: Superficial
**ID1**	1.7187655	0E+00	F1: Superficial
**COL13A1**	3.472152	0E+00	F1: Superficial
**AXIN2**	2.5300653	0E+00	F1: Superficial
**LAMC3**	1.7949047	0E+00	F1: Superficial
**SPON1**	1.9402529	0E+00	F1: Superficial
**TNFRSF19**	3.218595	0E+00	F1: Superficial
**PTK7**	2.1809254	0E+00	F1: Superficial
**ROBO2**	1.3872069	0E+00	F1: Superficial
**RSPO1**	2.9386795	0E+00	F1: Superficial
**AQP1**	1.2224329	0E+00	F1: Superficial
**MOXD1**	2.5611103	0E+00	F1: Superficial
**COL14A1**	1.3606474	0E+00	F1: Superficial
**PAM**	1.0005938	0E+00	F1: Superficial
**MYO10**	2.3089144	0E+00	F1: Superficial
**PHACTR3**	4.595881	0E+00	F1: Superficial
**LSAMP**	1.7322274	0E+00	F1: Superficial
**MEF2A**	1.213276	0E+00	F1: Superficial
**CYP26B1**	2.1429868	0E+00	F1: Superficial
**COL1A2**	0.8834083	0E+00	F1: Superficial
**PLCB4**	2.426451	0E+00	F1: Superficial
**SPRY1**	1.1983483	0E+00	F1: Superficial
**NKD1**	2.4069073	0E+00	F1: Superficial
**BGN**	1.2098932	0E+00	F1: Superficial
**NPTX2**	4.8313937	0E+00	F1: Superficial
**ARRDC3**	1.3552003	0E+00	F1: Superficial
**AKAP6**	2.8500195	0E+00	F1: Superficial
**SVIL**	1.0635065	0E+00	F1: Superficial
**ZBTB20**	0.98861694	0E+00	F1: Superficial
**SEMA6D**	4.2302494	0E+00	F1: Superficial
**CLEC11A**	1.287591	0E+00	F1: Superficial
**PREX2**	1.7662374	0E+00	F1: Superficial
**ISM1**	1.7860656	0E+00	F1: Superficial
**NTM**	2.6606362	0E+00	F1: Superficial

**MT-CO1**	0.6650337	0E+00	F1: Superficial
**GNG11**	1.2078081	0E+00	F1: Superficial
**EFNA5**	1.4719862	0E+00	F1: Superficial
**GAPDH**	0.6630245	0E+00	F1: Superficial
**ZFP36L1**	0.7704866	0E+00	F1: Superficial
**GRIK2**	2.682892	0E+00	F1: Superficial
**COL6A5**	4.0487022	0E+00	F1: Superficial
**FST**	1.5365963	0E+00	F1: Superficial
**COL6A3**	0.95874405	0E+00	F1: Superficial
**LINC01091**	1.7214837	0E+00	F1: Superficial
**PHLDA1**	1.4390583	0E+00	F1: Superficial
**LGALS1**	0.52130294	0E+00	F1: Superficial
**STC2**	4.2620335	0E+00	F1: Superficial
**ROR2**	1.5112475	0E+00	F1: Superficial
**LINC00327**	3.8322685	0E+00	F1: Superficial
**ITM2C**	1.5191864	0E+00	F1: Superficial
**F10**	1.1593957	0E+00	F1: Superficial
**RGS3**	2.1392124	0E+00	F1: Superficial
**HMCN1**	1.4842919	0E+00	F1: Superficial
**CCBE1**	1.2819012	0E+00	F1: Superficial
**AC068587.4**	1.2317728	0E+00	F1: Superficial
**DCHS2**	4.247016	0E+00	F1: Superficial
**PAPPA**	1.7312536	0E+00	F1: Superficial
**PTBP2**	1.4550042	0E+00	F1: Superficial
**RAB31**	1.5114827	0E+00	F1: Superficial
**AC079298.3**	2.0121064	0E+00	F1: Superficial
**PSD3**	1.1171944	0E+00	F1: Superficial
**OSBP2**	3.2203383	0E+00	F1: Superficial
**NFATC2**	1.5358809	0E+00	F1: Superficial
**ANPEP**	1.3071092	0E+00	F1: Superficial
**COL21A1**	1.9777274	0E+00	F1: Superficial
**RND3**	0.9403119	0E+00	F1: Superficial
**ZNF608**	1.8306848	0E+00	F1: Superficial
**AHRR**	2.7456636	0E+00	F1: Superficial

**DCN**	1.6106381	0E+00	F2: Universal
**CCN5**	2.8638284	0E+00	F2: Universal
**CTHRC1**	3.0100284	0E+00	F2: Universal
**FBLN1**	2.1041753	0E+00	F2: Universal
**PI16**	2.6601024	0E+00	F2: Universal
**SLPI**	4.0258694	0E+00	F2: Universal
**GSN**	1.6008888	0E+00	F2: Universal
**CFD**	1.7638402	0E+00	F2: Universal
**IGFBP6**	2.4021833	0E+00	F2: Universal
**MFAP5**	2.5973125	0E+00	F2: Universal
**S100A6**	1.0292933	0E+00	F2: Universal
**MMP2**	1.7312342	0E+00	F2: Universal
**PCOLCE2**	2.6928658	0E+00	F2: Universal
**CRYAB**	1.9090284	0E+00	F2: Universal
**SCARA5**	2.2684956	0E+00	F2: Universal
**GPX3**	2.719557	0E+00	F2: Universal
**MGP**	1.5587647	0E+00	F2: Universal
**S100A10**	0.9882741	0E+00	F2: Universal
**ACTB**	1.086161	0E+00	F2: Universal
**PLAC9**	1.2348548	0E+00	F2: Universal
**TSPAN8**	2.577873	0E+00	F2: Universal
**SH3BGRL3**	1.4984418	0E+00	F2: Universal
**CLU**	1.7094722	0E+00	F2: Universal
**FTH1**	0.9335756	0E+00	F2: Universal
**SEMA3C**	2.2591882	0E+00	F2: Universal
**TMSB4X**	1.1194419	0E+00	F2: Universal
**MGST1**	1.9565477	0E+00	F2: Universal
**CPE**	1.6719332	0E+00	F2: Universal
**CD70**	3.6898108	0E+00	F2: Universal
**CD99**	1.0449524	0E+00	F2: Universal
**SOD3**	1.135919	0E+00	F2: Universal
**CST3**	0.96624833	0E+00	F2: Universal
**CTSK**	1.2177515	0E+00	F2: Universal
**CYB5R3**	1.2970082	0E+00	F2: Universal

**CYBRD1**	1.2479725	0E+00	F2: Universal
**GPX4**	1.0533689	0E+00	F2: Universal
**QPCT**	1.8457034	0E+00	F2: Universal
**IGFBP5**	1.4076688	0E+00	F2: Universal
**MTRNR2L8**	1.4485601	0E+00	F2: Universal
**TMSB10**	0.73923707	0E+00	F2: Universal
**S100A4**	0.9683212	0E+00	F2: Universal
**CRIP1**	1.1289053	0E+00	F2: Universal
**ADIRF**	1.0244935	0E+00	F2: Universal
**ANXA2**	0.8125742	0E+00	F2: Universal
**FBLN2**	1.1918566	0E+00	F2: Universal
**LGALS3**	0.91754675	0E+00	F2: Universal
**CD34**	1.8732611	0E+00	F2: Universal
**TPM1**	1.3567045	0E+00	F2: Universal
**NPC2**	1.1575879	0E+00	F2: Universal
**ACTG1**	0.88525635	0E+00	F2: Universal
**CD55**	1.4383478	0E+00	F2: Universal
**FAM180B**	2.1693728	0E+00	F2: Universal
**THBS2**	1.2102741	0E+00	F2: Universal
**FTL**	0.65843177	0E+00	F2: Universal
**CXCL14**	1.1341965	0E+00	F2: Universal
**DPP4**	2.1925902	0E+00	F2: Universal
**MFAP4**	1.0630645	0E+00	F2: Universal
**TIMP3**	1.0587039	0E+00	F2: Universal
**DBN1**	2.0283859	0E+00	F2: Universal
**TIMP2**	0.9908762	0E+00	F2: Universal
**GABARAP**	0.89555854	0E+00	F2: Universal
**C1QTNF3**	2.2708685	0E+00	F2: Universal
**PDGFRL**	1.3740938	0E+00	F2: Universal
**SERPINF1**	0.9334651	0E+00	F2: Universal
**CHRDL1**	1.9458612	0E+00	F2: Universal
**POLR2L**	0.9479291	0E+00	F2: Universal
**LGALS1**	0.65154535	0E+00	F2: Universal
**MATN4**	4.2829194	0E+00	F2: Universal

**LGR5**	2.5844126	0E+00	F2: Universal
**FP700111.1**	1.4863367	0E+00	F2: Universal
**RPL27A**	0.7237508	0E+00	F2: Universal
**XG**	1.2484443	0E+00	F2: Universal
**GALNT15**	2.9082296	0E+00	F2: Universal
**PRG4**	4.71854	0E+00	F2: Universal
**ELN**	1.2264076	0E+00	F2: Universal
**GSTP1**	0.82772136	0E+00	F2: Universal
**HPGD**	1.9226574	0E+00	F2: Universal
**VAT1**	1.369873	0E+00	F2: Universal
**GYPC**	1.1830108	0E+00	F2: Universal
**KLF6**	0.99374574	0E+00	F2: Universal
**ITGB1BP1**	1.1105381	0E+00	F2: Universal
**CLTB**	1.1233715	0E+00	F2: Universal
**SLC29A1**	1.5007366	0E+00	F2: Universal
**NOVA1**	1.0772443	0E+00	F2: Universal
**RPLP2**	0.6038998	0E+00	F2: Universal
**MAP1B**	0.97841865	0E+00	F2: Universal
**RPL13A**	0.69739217	0E+00	F2: Universal
**CILP**	1.9228475	0E+00	F2: Universal
**CD151**	1.1821316	0E+00	F2: Universal
**CAPS**	1.8403206	0E+00	F2: Universal
**CADM3**	1.6101635	0E+00	F2: Universal
**LOX**	1.1387013	0E+00	F2: Universal
**ANGPTL5**	1.3559483	0E+00	F2: Universal
**TGFBR3**	1.047601	0E+00	F2: Universal
**ANGPTL1**	1.2187468	0E+00	F2: Universal
**ADM**	1.2490009	0E+00	F2: Universal
**SEMA3E**	2.882339	0E+00	F2: Universal
**RPS17**	0.70093614	0E+00	F2: Universal
**DDAH2**	0.90501255	0E+00	F2: Universal
**RPL37A**	0.55500853	0E+00	F2: Universal
**APOE**	3.3281872	0E+00	F2/3: Perivascular
**APOD**	1.9495009	0E+00	F2/3: Perivascular

**CXCL12**	1.7135028	0E+00	F2/3: Perivascular
**IGFBP7**	1.4643209	0E+00	F2/3: Perivascular
**GSN**	0.93616754	0E+00	F2/3: Perivascular
**CXCL2**	2.097966	0E+00	F2/3: Perivascular
**CFD**	0.8746497	0E+00	F2/3: Perivascular
**EFEMP1**	1.6387869	0E+00	F2/3: Perivascular
**APOC1**	3.4580748	0E+00	F2/3: Perivascular
**RARRES2**	1.578725	0E+00	F2/3: Perivascular
**ITM2A**	2.3238502	0E+00	F2/3: Perivascular
**RPLP0**	0.8431701	0E+00	F2/3: Perivascular
**MGP**	0.96425927	0E+00	F2/3: Perivascular
**GPC3**	2.0542274	0E+00	F2/3: Perivascular
**CCL2**	1.6634421	0E+00	F2/3: Perivascular
**RPS8**	0.5327155	0E+00	F2/3: Perivascular
**RPS27A**	0.5054674	0E+00	F2/3: Perivascular
**RPL13**	0.4980249	0E+00	F2/3: Perivascular
**RPS2**	0.653422	0E+00	F2/3: Perivascular
**ARID5B**	1.0547991	0E+00	F2/3: Perivascular
**RPS18**	0.53579944	0E+00	F2/3: Perivascular
**SOD2**	1.0987084	0E+00	F2/3: Perivascular
**CYGB**	2.3659518	0E+00	F2/3: Perivascular
**IGF1**	2.2176874	0E+00	F2/3: Perivascular
**NFIB**	1.1925712	0E+00	F2/3: Perivascular
**H2AFZ**	1.0995817	0E+00	F2/3: Perivascular
**RPL11**	0.42916667	0E+00	F2/3: Perivascular
**C7**	3.110235	0E+00	F2/3: Perivascular
**TSHZ2**	1.6143961	0E+00	F2/3: Perivascular
**C3**	1.2267655	0E+00	F2/3: Perivascular
**MYOC**	3.8656697	0E+00	F2/3: Perivascular
**RPL32**	0.45033455	0E+00	F2/3: Perivascular
**GGT5**	2.1372128	0E+00	F2/3: Perivascular
**FGF7**	1.3860499	0E+00	F2/3: Perivascular
**RPLP1**	0.45638093	0E+00	F2/3: Perivascular
**PNRC1**	0.8465767	0E+00	F2/3: Perivascular

**GEM**	1.0821993	0E+00	F2/3: Perivascular
**GAS5**	0.853028	0E+00	F2/3: Perivascular
**RPL18**	0.48825508	0E+00	F2/3: Perivascular
**RPS15A**	0.45260608	0E+00	F2/3: Perivascular
**RPL21**	0.49709234	0E+00	F2/3: Perivascular
**LSP1**	1.1791825	0E+00	F2/3: Perivascular
**RPS3**	0.48018128	0E+00	F2/3: Perivascular
**ERRFI1**	1.3041248	0E+00	F2/3: Perivascular
**RPS9**	0.4708125	0E+00	F2/3: Perivascular
**CXCL3**	1.6126438	0E+00	F2/3: Perivascular
**BTG1**	0.84180605	0E+00	F2/3: Perivascular
**CFH**	0.8093515	0E+00	F2/3: Perivascular
**IGFBP3**	1.5595946	0E+00	F2/3: Perivascular
**RPS15**	0.39160296	0E+00	F2/3: Perivascular
**RPL24**	0.44503137	0E+00	F2/3: Perivascular
**RPS24**	0.40588707	0E+00	F2/3: Perivascular
**ABCA8**	1.2860519	6.0081E-307	F2/3: Perivascular
**RPL34**	0.38916585	5.31556E-302	F2/3: Perivascular
**NFKBIA**	0.75391346	1.87256E-297	F2/3: Perivascular
**RPS5**	0.44728813	9.12585E-296	F2/3: Perivascular
**FAU**	0.4002399	8.4443E-286	F2/3: Perivascular
**RPS14**	0.4026589	1.47832E-285	F2/3: Perivascular
**FMO1**	3.7211583	9.04368E-282	F2/3: Perivascular
**CDKN1A**	0.90072143	2.8454E-280	F2/3: Perivascular
**PLA2G2A**	1.4884609	1.07266E-278	F2/3: Perivascular
**RPL8**	0.40885824	1.7802E-279	F2/3: Perivascular
**PPARG**	3.3090441	4.28845E-272	F2/3: Perivascular
**RPS23**	0.3588319	2.16674E-271	F2/3: Perivascular
**CCDC80**	0.5921372	7.61477E-269	F2/3: Perivascular
**RPS13**	0.3871498	4.95283E-268	F2/3: Perivascular
**KDM6B**	1.3307867	2.29597E-264	F2/3: Perivascular
**PRRX1**	0.7619974	9.83368E-263	F2/3: Perivascular
**RPS27**	0.4214169	6.29253E-263	F2/3: Perivascular
**TPT1**	0.40802965	1.47252E-261	F2/3: Perivascular

**SPSB1**	1.2205986	7.47913E-257	F2/3: Perivascular
**RPS16**	0.44977066	1.41561E-257	F2/3: Perivascular
**HSPE1**	0.7815056	1.82817E-250	F2/3: Perivascular
**RPL10A**	0.43560544	8.46324E-251	F2/3: Perivascular
**WTAP**	0.83481795	1.76518E-249	F2/3: Perivascular
**FAM241A**	1.7819755	2.77471E-246	F2/3: Perivascular
**EBF1**	0.89271635	2.91187E-247	F2/3: Perivascular
**RPL23**	0.50841826	3.35076E-243	F2/3: Perivascular
**PPP1R15A**	0.6846835	3.80896E-242	F2/3: Perivascular
**RPL37**	0.38560417	3.03626E-241	F2/3: Perivascular
**RPL14**	0.36906108	1.62221E-236	F2/3: Perivascular
**RPL30**	0.3432008	2.70302E-231	F2/3: Perivascular
**RPL35A**	0.3468602	5.03448E-231	F2/3: Perivascular
**CYP1B1**	1.158707	2.56752E-229	F2/3: Perivascular
**SRPX**	1.0182854	4.13093E-228	F2/3: Perivascular
**RPS12**	0.34133607	1.31147E-228	F2/3: Perivascular
**RPL23A**	0.39167303	2.53906E-223	F2/3: Perivascular
**RPL3**	0.38198012	1.37644E-221	F2/3: Perivascular
**MT1A**	1.4590722	1.37537E-219	F2/3: Perivascular
**RPS3A**	0.35344118	1.49492E-219	F2/3: Perivascular
**RPL9**	0.38911197	3.58624E-219	F2/3: Perivascular
**RPS29**	0.4628942	8.42299E-219	F2/3: Perivascular
**COL4A1**	1.2517049	1.28755E-217	F2/3: Perivascular
**COL4A2**	1.1397034	8.94395E-217	F2/3: Perivascular
**PPP1R15B**	1.2453206	9.38405E-213	F2/3: Perivascular
**RPL29**	0.35227764	4.00398E-213	F2/3: Perivascular
**RPL17**	0.3767666	2.87012E-210	F2/3: Perivascular
**ARL6IP1**	0.8232906	9.19942E-209	F2/3: Perivascular
**RPL10**	0.30433878	2.59424E-208	F2/3: Perivascular
**TMEM176A**	1.0038916	5.07067E-206	F2/3: Perivascular
**APOE**	4.497994	0E+00	F3: FRC-like
**IGFBP7**	2.0730798	0E+00	F3: FRC-like
**CXCL12**	2.0472832	0E+00	F3: FRC-like
**PTGDS**	3.0800304	0E+00	F3: FRC-like

**CCL19**	6.802455	0E+00	F3: FRC-like
**HLA-B**	1.7890172	0E+00	F3: FRC-like
**C3**	2.343156	0E+00	F3: FRC-like
**B2M**	0.8652237	0E+00	F3: FRC-like
**CYP7B1**	2.4625828	0E+00	F3: FRC-like
**RARRES2**	1.8302442	0E+00	F3: FRC-like
**MT-CO3**	0.9751386	0E+00	F3: FRC-like
**HAS2**	1.8157134	0E+00	F3: FRC-like
**CCL2**	1.9952043	0E+00	F3: FRC-like
**CD74**	4.0201793	0E+00	F3: FRC-like
**MT-ND3**	1.2099371	0E+00	F3: FRC-like
**MT-CO1**	0.84148395	0E+00	F3: FRC-like
**TNFSF13B**	2.9530957	0E+00	F3: FRC-like
**GGT5**	2.7322147	0E+00	F3: FRC-like
**RBP5**	4.10238	0E+00	F3: FRC-like
**NRP1**	1.7268828	0E+00	F3: FRC-like
**UGCG**	2.0972524	0E+00	F3: FRC-like
**IFITM3**	0.9031154	0E+00	F3: FRC-like
**SNED1**	1.5136539	0E+00	F3: FRC-like
**BIRC3**	2.5861742	0E+00	F3: FRC-like
**TMEM176B**	1.4334277	0E+00	F3: FRC-like
**CTSH**	2.3398502	0E+00	F3: FRC-like
**RGS16**	1.5211133	0E+00	F3: FRC-like
**VCAM1**	2.2982059	0E+00	F3: FRC-like
**ID4**	2.1664088	0E+00	F3: FRC-like
**OSMR**	1.8858984	0E+00	F3: FRC-like
**CXCL3**	2.0662286	0E+00	F3: FRC-like
**HLA-A**	0.8990794	0E+00	F3: FRC-like
**TMEM176A**	1.5166705	0E+00	F3: FRC-like
**CXCL1**	2.3843865	0E+00	F3: FRC-like
**FILIP1L**	1.4648033	0E+00	F3: FRC-like
**IL32**	2.2613275	0E+00	F3: FRC-like
**IGFBP3**	1.9968487	0E+00	F3: FRC-like
**C7**	3.1937544	0E+00	F3: FRC-like

**MT-CO2**	0.67009103	0E+00	F3: FRC-like
**CH25H**	3.0127919	0E+00	F3: FRC-like
**TNFAIP3**	1.8101258	0E+00	F3: FRC-like
**JUN**	1.0437189	0E+00	F3: FRC-like
**LIFR**	1.7368567	0E+00	F3: FRC-like
**IRF8**	4.3714747	0E+00	F3: FRC-like
**SLC2A3**	1.2823269	0E+00	F3: FRC-like
**SOD2**	1.079637	0E+00	F3: FRC-like
**OLFM2**	2.0828152	0E+00	F3: FRC-like
**CLSTN3**	2.5150018	0E+00	F3: FRC-like
**IL33**	2.9453652	0E+00	F3: FRC-like
**TMEM132C**	2.486509	0E+00	F3: FRC-like
**LAMA4**	1.1645154	0E+00	F3: FRC-like
**GEM**	1.1691661	0E+00	F3: FRC-like
**CYGB**	2.2675211	0E+00	F3: FRC-like
**PLEKHH2**	1.4067948	0E+00	F3: FRC-like
**APOC1**	2.4363575	0E+00	F3: FRC-like
**MT-ATP6**	0.7468905	0E+00	F3: FRC-like
**IL34**	2.565944	0E+00	F3: FRC-like
**SDK1**	1.6050925	0E+00	F3: FRC-like
**CTSC**	2.1446576	0E+00	F3: FRC-like
**ABCA10**	1.8697191	0E+00	F3: FRC-like
**HLA-F**	2.452352	0E+00	F3: FRC-like
**HES4**	2.4414883	0E+00	F3: FRC-like
**CCDC80**	0.6963376	0E+00	F3: FRC-like
**ST8SIA1**	3.1248007	0E+00	F3: FRC-like
**TNFSF10**	1.6631163	0E+00	F3: FRC-like
**AHR**	1.0778766	0E+00	F3: FRC-like
**VEGFA**	1.697629	0E+00	F3: FRC-like
**DCLK1**	1.0524374	0E+00	F3: FRC-like
**LDB2**	1.3129498	0E+00	F3: FRC-like
**SPARCL1**	1.4777378	0E+00	F3: FRC-like
**ENPP2**	1.4113771	0E+00	F3: FRC-like
**LSAMP**	1.3539153	0E+00	F3: FRC-like

**PHLDA1**	1.3506517	0E+00	F3: FRC-like
**SPATS2L**	1.2009093	0E+00	F3: FRC-like
**TMEM150C**	2.806382	1.09116E-306	F3: FRC-like
**ICAM1**	1.1473126	2.46293E-308	F3: FRC-like
**ADRA2A**	3.3225744	6.88624E-304	F3: FRC-like
**FLT3LG**	1.5692811	3.29759E-302	F3: FRC-like
**PTPRT**	3.7344913	1.88593E-300	F3: FRC-like
**EBF1**	1.0275695	4.6567E-299	F3: FRC-like
**RND3**	0.9302943	2.1364E-298	F3: FRC-like
**CCDC146**	1.4933532	3.28665E-295	F3: FRC-like
**NFIA**	0.8730845	1.21721E-295	F3: FRC-like
**PRRX1**	0.8287132	4.99641E-291	F3: FRC-like
**COL4A2**	1.2814623	5.59594E-290	F3: FRC-like
**IRF1**	1.0299243	1.88468E-289	F3: FRC-like
**NLGN4X**	3.4014375	7.02902E-286	F3: FRC-like
**ABI3BP**	0.9508522	1.5021E-286	F3: FRC-like
**TYMP**	1.858104	3.31037E-281	F3: FRC-like
**ZFP36**	0.8678356	2.22941E-272	F3: FRC-like
**CXCL2**	1.259788	5.3221E-271	F3: FRC-like
**JUNB**	0.72670794	5.4021E-270	F3: FRC-like
**PRKG1**	1.0815709	1.75797E-267	F3: FRC-like
**SOCS3**	0.93642986	1.28414E-262	F3: FRC-like
**SNAI2**	1.4771559	1.18996E-261	F3: FRC-like
**PSME2**	1.1918093	3.21877E-261	F3: FRC-like
**ABCA8**	1.1766895	3.74139E-260	F3: FRC-like
**IFI16**	0.8359802	8.54599E-254	F3: FRC-like
**MSC**	2.4435594	2.24158E-251	F3: FRC-like
**HES1**	0.9194683	2.71454E-252	F3: FRC-like
**POSTN**	3.6006145	0E+00	F4: DS_DPEP1+
**COL11A1**	5.9211936	0E+00	F4: DS_DPEP1+
**PCDH15**	4.459966	0E+00	F4: DS_DPEP1+
**COL5A2**	1.7169722	0E+00	F4: DS_DPEP1+
**ASPN**	3.2941723	0E+00	F4: DS_DPEP1+
**PRKG1**	1.9454099	0E+00	F4: DS_DPEP1+

**SEPTIN11**	1.7834235	0E+00	F4: DS_DPEP1+
**CDH11**	2.1387541	0E+00	F4: DS_DPEP1+
**AOPEP**	1.7403216	0E+00	F4: DS_DPEP1+
**CALD1**	1.2357894	0E+00	F4: DS_DPEP1+
**F2R**	2.248979	0E+00	F4: DS_DPEP1+
**PRSS23**	2.696621	0E+00	F4: DS_DPEP1+
**EDNRA**	3.4200323	0E+00	F4: DS_DPEP1+
**NEGR1**	1.589623	0E+00	F4: DS_DPEP1+
**TCF4**	1.2909771	0E+00	F4: DS_DPEP1+
**LINC00486**	2.3684607	0E+00	F4: DS_DPEP1+
**KIF26B**	4.7148123	0E+00	F4: DS_DPEP1+
**TRPS1**	1.8221066	0E+00	F4: DS_DPEP1+
**MARCKS**	1.4866823	0E+00	F4: DS_DPEP1+
**GPC3**	2.161621	0E+00	F4: DS_DPEP1+
**PALLD**	1.6790963	0E+00	F4: DS_DPEP1+
**TSHZ3**	2.3309925	0E+00	F4: DS_DPEP1+
**MEF2C**	2.709866	0E+00	F4: DS_DPEP1+
**MDK**	2.3135903	0E+00	F4: DS_DPEP1+
**KIAA1217**	2.7421806	0E+00	F4: DS_DPEP1+
**COLEC12**	1.9335885	0E+00	F4: DS_DPEP1+
**SGIP1**	3.4449482	0E+00	F4: DS_DPEP1+
**EDIL3**	3.4534085	0E+00	F4: DS_DPEP1+
**TPM2**	2.0506887	0E+00	F4: DS_DPEP1+
**COL15A1**	1.7618822	0E+00	F4: DS_DPEP1+
**TENM3**	3.781832	0E+00	F4: DS_DPEP1+
**MEIS2**	1.6594623	0E+00	F4: DS_DPEP1+
**JMJD1C**	1.2416978	0E+00	F4: DS_DPEP1+
**HTRA1**	1.3715874	0E+00	F4: DS_DPEP1+
**COL6A3**	1.2235156	0E+00	F4: DS_DPEP1+
**PMEPA1**	1.9518852	0E+00	F4: DS_DPEP1+
**SFRP1**	2.558155	0E+00	F4: DS_DPEP1+
**COL1A1**	1.224367	0E+00	F4: DS_DPEP1+
**ROR2**	1.8485222	0E+00	F4: DS_DPEP1+
**MCC**	2.0952122	0E+00	F4: DS_DPEP1+

**WWTR1**	1.3721281	0E+00	F4: DS_DPEP1+
**DKK 2.00**	1.6342927	0E+00	F4: DS_DPEP1+
**COL3A1**	1.1698706	0E+00	F4: DS_DPEP1+
**THSD4**	2.849416	0E+00	F4: DS_DPEP1+
**PTN**	1.5953535	0E+00	F4: DS_DPEP1+
**TNN**	4.4369454	0E+00	F4: DS_DPEP1+
**PPP1R14A**	5.174145	0E+00	F4: DS_DPEP1+
**PRRX1**	1.0632117	0E+00	F4: DS_DPEP1+
**ADAMTS9-/**	1.7294703	0E+00	F4: DS_DPEP1+
**FMNL2**	1.6064209	0E+00	F4: DS_DPEP1+
**OGN**	2.0638797	0E+00	F4: DS_DPEP1+
**DPEP1**	7.2088013	0E+00	F4: DS_DPEP1+
**LRRC17**	2.410362	0E+00	F4: DS_DPEP1+
**ADAMTS12**	2.3339865	0E+00	F4: DS_DPEP1+
**IGF1**	2.442187	0E+00	F4: DS_DPEP1+
**TBX15**	1.7870073	0E+00	F4: DS_DPEP1+
**MXRA5**	1.5569202	0E+00	F4: DS_DPEP1+
**MME**	4.0029526	0E+00	F4: DS_DPEP1+
**EMP2**	1.392177	0E+00	F4: DS_DPEP1+
**RUNX1T1**	1.437739	0E+00	F4: DS_DPEP1+
**SESN3**	2.1566722	0E+00	F4: DS_DPEP1+
**TANC1**	1.7014741	0E+00	F4: DS_DPEP1+
**LAMA2**	1.256245	0E+00	F4: DS_DPEP1+
**SPATS2L**	1.4567715	0E+00	F4: DS_DPEP1+
**SPON1**	1.7422497	0E+00	F4: DS_DPEP1+
**FOXP2**	1.745607	0E+00	F4: DS_DPEP1+
**COL1A2**	0.8510719	0E+00	F4: DS_DPEP1+
**THY1**	1.3213723	0E+00	F4: DS_DPEP1+
**FBXL7**	1.4564633	0E+00	F4: DS_DPEP1+
**COL6A6**	2.7703369	0E+00	F4: DS_DPEP1+
**ARHGAP42**	2.354125	0E+00	F4: DS_DPEP1+
**DOK6**	2.2582119	0E+00	F4: DS_DPEP1+
**TCF7L2**	1.1467113	0E+00	F4: DS_DPEP1+
**PDE1A**	3.1231089	0E+00	F4: DS_DPEP1+

**UTRN**	1.4066151	0E+00	F4: DS_DPEP1+
**CASC15**	3.3097568	0E+00	F4: DS_DPEP1+
**SEMA5A**	2.295691	0E+00	F4: DS_DPEP1+
**GPM6B**	2.9314508	0E+00	F4: DS_DPEP1+
**SH3KBP1**	1.3388233	0E+00	F4: DS_DPEP1+
**MEG3**	1.0222188	0E+00	F4: DS_DPEP1+
**DKK 3.00**	1.5018764	0E+00	F4: DS_DPEP1+
**RUNX2**	3.4518812	0E+00	F4: DS_DPEP1+
**RBFOX1**	3.603483	0E+00	F4: DS_DPEP1+
**TTC3**	1.0557696	0E+00	F4: DS_DPEP1+
**GNAQ**	1.3057387	8.31258E-310	F4: DS_DPEP1+
**RBMS3**	1.0875223	9.3483E-306	F4: DS_DPEP1+
**INHBA**	2.447454	1.1017E-299	F4: DS_DPEP1+
**ZBTB20**	0.9109038	4.18953E-299	F4: DS_DPEP1+
**ATP10A**	1.6271285	3.06316E-293	F4: DS_DPEP1+
**TEAD1**	1.3367894	4.62375E-293	F4: DS_DPEP1+
**TNMD**	2.7672231	8.10039E-288	F4: DS_DPEP1+
**AUTS2**	1.1837897	1.24761E-288	F4: DS_DPEP1+
**GLI2**	2.3658957	5.74382E-284	F4: DS_DPEP1+
**RAI14**	2.0962815	1.57232E-282	F4: DS_DPEP1+
**BACH2**	1.8136147	7.17029E-282	F4: DS_DPEP1+
**TMEM119**	1.786393	9.89228E-282	F4: DS_DPEP1+
**TLL1**	3.2172174	2.08532E-280	F4: DS_DPEP1+
**LTBP2**	2.562743	5.10317E-279	F4: DS_DPEP1+
**ROBO1**	1.2607552	1.19078E-279	F4: DS_DPEP1+
**ASAP1**	1.3370059	9.20938E-279	F4: DS_DPEP1+
**COCH**	9.334681	0E+00	F4: TNN+COCH+
**ASPN**	5.7819915	0E+00	F4: TNN+COCH+
**SPARCL1**	4.822926	0E+00	F4: TNN+COCH+
**OGN**	4.256744	0E+00	F4: TNN+COCH+
**PTGDS**	3.5890207	2.25093E-314	F4: TNN+COCH+
**TRPS1**	3.435415	5.0773E-291	F4: TNN+COCH+
**TCF4**	2.3737872	4.81007E-292	F4: TNN+COCH+
**TNN**	6.2317863	1.05626E-286	F4: TNN+COCH+

**CRABP1**	5.745813	1.40273E-263	F4: TNN+COCH+
**AOPEP**	2.5797725	1.51037E-238	F4: TNN+COCH+
**FIBIN**	3.301298	4.97086E-235	F4: TNN+COCH+
**NRG3**	8.812568	2.59605E-224	F4: TNN+COCH+
**COL24A1**	4.829216	8.53461E-223	F4: TNN+COCH+
**IGFBP5**	2.1256995	5.2942E-220	F4: TNN+COCH+
**CADM2**	5.594198	9.78491E-214	F4: TNN+COCH+
**PLXDC1**	3.9810557	4.3227E-205	F4: TNN+COCH+
**HPSE2**	3.432281	2.18298E-203	F4: TNN+COCH+
**PCDH15**	4.5246906	3.00201E-200	F4: TNN+COCH+
**CYP1B1**	2.7927096	2.63244E-196	F4: TNN+COCH+
**TRPM3**	5.594304	2.82708E-193	F4: TNN+COCH+
**DKK 2.00**	2.6092691	9.43716E-190	F4: TNN+COCH+
**EMID1**	3.7318587	8.42729E-188	F4: TNN+COCH+
**PLPP5**	4.1931853	2.3215E-186	F4: TNN+COCH+
**MALAT1**	0.65804493	7.35048E-180	F4: TNN+COCH+
**PXDNL**	5.6009784	1.29563E-170	F4: TNN+COCH+
**FZD1**	3.6047206	1.27721E-170	F4: TNN+COCH+
**TNMD**	4.2455597	8.81955E-167	F4: TNN+COCH+
**COL1A1**	1.2167025	1.85118E-169	F4: TNN+COCH+
**MXRA5**	2.4120784	2.41707E-166	F4: TNN+COCH+
**ROBO2**	2.1558917	3.4972E-166	F4: TNN+COCH+
**F13A1**	2.5974982	1.25378E-164	F4: TNN+COCH+
**COL1A2**	0.9769378	2.16651E-163	F4: TNN+COCH+
**SFRP1**	3.1274254	4.4782E-159	F4: TNN+COCH+
**MKX**	5.3866196	9.26775E-158	F4: TNN+COCH+
**MEIS2**	2.3853967	9.91968E-157	F4: TNN+COCH+
**ARHGAP15**	4.6131053	5.56913E-154	F4: TNN+COCH+
**LIMCH1**	2.6702096	1.20035E-153	F4: TNN+COCH+
**MEOX2**	3.0536585	8.21454E-153	F4: TNN+COCH+
**MARCKS**	1.8291738	2.0294E-145	F4: TNN+COCH+
**RSPO4**	4.318102	2.7542E-141	F4: TNN+COCH+
**GPM6B**	3.8524313	3.93847E-141	F4: TNN+COCH+
**HSPA2**	3.0787885	1.02869E-137	F4: TNN+COCH+

**SRPX**	2.0337672	4.40282E-133	F4: TNN+COCH+
**ZBTB20**	1.4801713	3.44635E-133	F4: TNN+COCH+
**FMOD**	2.2707932	4.23804E-132	F4: TNN+COCH+
**KREMEN1**	2.4798784	3.86361E-130	F4: TNN+COCH+
**CALM2**	1.0946656	7.49099E-124	F4: TNN+COCH+
**COL11A1**	3.6388085	2.91355E-122	F4: TNN+COCH+
**NDNF**	4.926656	3.31597E-120	F4: TNN+COCH+
**FGF13**	5.162684	3.71899E-119	F4: TNN+COCH+
**PTPRK**	2.3504639	4.45594E-119	F4: TNN+COCH+
**PTPRD**	4.6422215	1.07962E-117	F4: TNN+COCH+
**NBEA**	2.7463534	1.16675E-117	F4: TNN+COCH+
**PTGER3**	2.8988116	6.70414E-116	F4: TNN+COCH+
**PLPP1**	1.7681619	4.25255E-115	F4: TNN+COCH+
**HTRA1**	1.6746576	2.74021E-113	F4: TNN+COCH+
**PCSK1N**	3.3795063	8.10806E-113	F4: TNN+COCH+
**AC090994.1**	6.677214	1.12899E-112	F4: TNN+COCH+
**PGRMC1**	1.627202	1.25977E-112	F4: TNN+COCH+
**EDNRA**	3.094733	1.85508E-110	F4: TNN+COCH+
**ADAMTS6**	5.12269	1.29481E-105	F4: TNN+COCH+
**CTSK**	0.99281996	5.82084E-106	F4: TNN+COCH+
**DLG2**	2.0471957	1.44464E-105	F4: TNN+COCH+
**ITGA11**	2.5231917	2.05237E-104	F4: TNN+COCH+
**NECAB1**	4.9754715	3.08703E-103	F4: TNN+COCH+
**THY1**	1.6668464	6.69807E-103	F4: TNN+COCH+
**SLITRK6**	7.8290615	6.75607E-102	F4: TNN+COCH+
**PRELP**	1.6478897	2.44484E-101	F4: TNN+COCH+
**TSHZ3**	2.3921423	1.38642E-100	F4: TNN+COCH+
**PCDH7**	3.724125	4.25393E-100	F4: TNN+COCH+
**MAFB**	1.7728367	1.15135E-99	F4: TNN+COCH+
**FN1**	1.2867404	1.11506E-97	F4: TNN+COCH+
**LINC00486**	2.547641	2.26842E-97	F4: TNN+COCH+
**BTBD11**	5.812877	1.92036E-95	F4: TNN+COCH+
**APCDD1**	1.7105112	3.96606E-95	F4: TNN+COCH+
**ENHO**	2.13182	7.40228E-95	F4: TNN+COCH+

**PTK7**	2.204	9.32784E-94	F4: TNN+COCH+
**CRABP2**	1.4485763	7.52422E-94	F4: TNN+COCH+
**PTGFR**	1.7356392	2.72734E-93	F4: TNN+COCH+
**AC079760.1**	4.889637	5.24128E-93	F4: TNN+COCH+
**AL049830.3**	7.1133466	5.15934E-92	F4: TNN+COCH+
**FOXP2**	2.0005357	8.17074E-92	F4: TNN+COCH+
**TENM3**	3.4163957	4.1297E-91	F4: TNN+COCH+
**SMAD1**	2.9818108	7.75817E-91	F4: TNN+COCH+
**PRKCB**	6.9856086	3.26101E-89	F4: TNN+COCH+
**CEMIP2**	2.1816194	3.20116E-89	F4: TNN+COCH+
**PTH1R**	2.4935725	4.56986E-89	F4: TNN+COCH+
**TANC1**	1.8845443	1.01998E-87	F4: TNN+COCH+
**CPNE5**	5.1188135	2.28473E-87	F4: TNN+COCH+
**BHLHE41**	2.4805527	7.23346E-86	F4: TNN+COCH+
**FAM3C**	1.4357088	8.54821E-86	F4: TNN+COCH+
**AUTS2**	1.518631	9.75741E-86	F4: TNN+COCH+
**LRIG1**	2.0652292	2.7601E-85	F4: TNN+COCH+
**AP001347.1**	2.5600958	3.56605E-84	F4: TNN+COCH+
**CNTN1**	1.8113996	1.98287E-83	F4: TNN+COCH+
**MRPS6**	1.5589485	2.397E-83	F4: TNN+COCH+
**NR2F1-AS1**	3.5377772	4.71811E-83	F4: TNN+COCH+
**SLC22A16**	6.902042	6.50066E-83	F4: TNN+COCH+
**CDH12**	4.935088	1.16697E-82	F4: TNN+COCH+
**LTBP4**	1.2353424	2.44621E-82	F4: TNN+COCH+
**TRPS1**	4.6500063	6.16132E-43	F4: DP_HHIP+
**LINC00486**	3.9519114	6.87647E-33	F4: DP_HHIP+
**CDH11**	3.8482141	3.11655E-31	F4: DP_HHIP+
**GNAS**	2.1812625	7.66203E-28	F4: DP_HHIP+
**IGFBP3**	5.3872557	1.69008E-27	F4: DP_HHIP+
**FP236383.3**	3.4086158	1.98329E-27	F4: DP_HHIP+
**JMJD1C**	2.2025652	5.31534E-26	F4: DP_HHIP+
**INHBA**	5.1704807	5.71153E-26	F4: DP_HHIP+
**WNT5A**	6.7405386	1.1776E-23	F4: DP_HHIP+
**PDE3A**	5.470429	5.42715E-22	F4: DP_HHIP+

**MRPS6**	2.709895	1.15354E-21	F4: DP_HHIP+
**DCC**	5.4949307	2.78825E-21	F4: DP_HHIP+
**PREX2**	3.3215222	2.79127E-21	F4: DP_HHIP+
**ROBO1**	2.7479897	5.09289E-21	F4: DP_HHIP+
**KIF26B**	5.232901	6.05382E-21	F4: DP_HHIP+
**SSBP2**	2.6089773	1.51278E-20	F4: DP_HHIP+
**ROBO2**	2.4352214	1.61459E-20	F4: DP_HHIP+
**PTCH1**	5.3368793	1.73478E-20	F4: DP_HHIP+
**LMO4**	2.439209	2.06437E-20	F4: DP_HHIP+
**PRKG1**	2.8023806	2.54787E-20	F4: DP_HHIP+
**SERPINE2**	3.2097914	3.91618E-20	F4: DP_HHIP+
**SPARCL1**	3.2610104	4.8682E-20	F4: DP_HHIP+
**MT-ATP6**	1.5256144	5.41917E-20	F4: DP_HHIP+
**APCDD1**	2.810203	7.94392E-20	F4: DP_HHIP+
**PTMA**	1.2415239	1.44374E-19	F4: DP_HHIP+
**MT-ND5**	1.4811528	1.54324E-19	F4: DP_HHIP+
**SPON1**	3.202745	2.01668E-19	F4: DP_HHIP+
**EDNRA**	4.219293	3.95929E-19	F4: DP_HHIP+
**TPD52**	7.94048	4.06149E-19	F4: DP_HHIP+
**DKK 3.00**	2.8832998	6.23855E-19	F4: DP_HHIP+
**DIO2**	3.589259	1.44792E-18	F4: DP_HHIP+
**MT-ND3**	1.5165973	4.28534E-18	F4: DP_HHIP+
**CPE**	1.8405486	5.043E-18	F4: DP_HHIP+
**CNTN4**	4.7527514	6.06747E-18	F4: DP_HHIP+
**TFAP2A**	7.6994815	9.37891E-18	F4: DP_HHIP+
**PRKD1**	3.5737503	2.69425E-17	F4: DP_HHIP+
**TCF4**	1.5419021	4.74817E-17	F4: DP_HHIP+
**AOPEP**	2.1473627	6.45003E-17	F4: DP_HHIP+
**COL23A1**	3.3585083	1.02226E-16	F4: DP_HHIP+
**LEF1**	6.145187	5.84781E-16	F4: DP_HHIP+
**SPIDR**	2.4559608	6.02161E-16	F4: DP_HHIP+
**DCXR**	3.8108099	1.24437E-15	F4: DP_HHIP+
**MT-CO1**	1.161598	1.35929E-15	F4: DP_HHIP+
**CENPW**	4.898286	2.08371E-15	F4: DP_HHIP+

**LINC01117**	4.0307097	9.92742E-15	F4: DP_HHIP+
**PRDM1**	4.2496605	1.27696E-14	F4: DP_HHIP+
**MT-CYB**	1.043815	1.33732E-14	F4: DP_HHIP+
**PRLR**	5.015764	1.43145E-14	F4: DP_HHIP+
**EDIL3**	4.456506	2.28161E-14	F4: DP_HHIP+
**LUZP2**	7.357299	2.34997E-14	F4: DP_HHIP+
**ALX4**	5.581495	3.17693E-14	F4: DP_HHIP+
**GRIK1**	5.8135867	3.59945E-14	F4: DP_HHIP+
**CBLB**	2.2804084	4.57652E-14	F4: DP_HHIP+
**ZBTB20**	1.4410939	4.90023E-14	F4: DP_HHIP+
**TANC1**	2.3904815	5.75989E-14	F4: DP_HHIP+
**PRRX1**	1.3774844	8.27189E-14	F4: DP_HHIP+
**ANKRD50**	3.59395	8.86391E-14	F4: DP_HHIP+
**HMGB1**	1.15336	1.36525E-13	F4: DP_HHIP+
**PKP4**	2.919456	1.46915E-13	F4: DP_HHIP+
**AL450332.1**	8.499667	1.54951E-13	F4: DP_HHIP+
**MCC**	2.956165	1.87948E-13	F4: DP_HHIP+
**IQGAP2**	5.07596	1.90467E-13	F4: DP_HHIP+
**CD81**	1.3860495	2.15898E-13	F4: DP_HHIP+
**PCMTD1**	2.0431705	2.62755E-13	F4: DP_HHIP+
**ADGRB3**	4.389613	2.89343E-13	F4: DP_HHIP+
**CDK19**	3.0146365	3.6744E-13	F4: DP_HHIP+
**PTPRG**	1.6793491	4.16683E-13	F4: DP_HHIP+
**CCDC3**	3.6767833	4.54715E-13	F4: DP_HHIP+
**TWIST1**	1.8703189	4.91941E-13	F4: DP_HHIP+
**RSRC1**	2.2818208	5.06165E-13	F4: DP_HHIP+
**FOXP2**	2.7179267	7.72647E-13	F4: DP_HHIP+
**LDLRAD3**	3.6859756	8.49228E-13	F4: DP_HHIP+
**PCM1**	1.7556778	9.71116E-13	F4: DP_HHIP+
**RERE**	2.0705533	1.29189E-12	F4: DP_HHIP+
**BMP7**	5.212531	1.40262E-12	F4: DP_HHIP+
**RBFOX2**	1.5420961	1.48463E-12	F4: DP_HHIP+
**MGAT5**	3.8203855	1.71158E-12	F4: DP_HHIP+
**CASC15**	4.084214	1.87823E-12	F4: DP_HHIP+

**SPON2**	2.0891793	1.92023E-12	F4: DP_HHIP+
**LINC01578**	1.8884956	2.18277E-12	F4: DP_HHIP+
**FTX**	2.1538453	3.20611E-12	F4: DP_HHIP+
**GLI2**	3.39641	4.70729E-12	F4: DP_HHIP+
**NRXN3**	6.8987355	5.04076E-12	F4: DP_HHIP+
**FBXO32**	3.6129975	6.13243E-12	F4: DP_HHIP+
**LAMB1**	2.6952176	6.33325E-12	F4: DP_HHIP+
**ADGRL3**	4.7009964	6.81738E-12	F4: DP_HHIP+
**ASPH**	1.7758676	6.78484E-12	F4: DP_HHIP+
**PGM2L1**	3.6657631	9.09892E-12	F4: DP_HHIP+
**SNHG14**	2.7265446	1.05258E-11	F4: DP_HHIP+
**PUM2**	2.0611768	1.10012E-11	F4: DP_HHIP+
**HNRNPA2B**	1.1468383	1.14625E-11	F4: DP_HHIP+
**NLGN1**	3.550895	1.37516E-11	F4: DP_HHIP+
**SOX18**	8.250438	1.39885E-11	F4: DP_HHIP+
**MEIS2**	1.8714856	1.47157E-11	F4: DP_HHIP+
**DGKH**	3.3848553	1.56056E-11	F4: DP_HHIP+
**SON**	1.2056338	1.57944E-11	F4: DP_HHIP+
**TNPO1**	2.2706494	2.81819E-11	F4: DP_HHIP+
**ARID1B**	1.7356796	2.82705E-11	F4: DP_HHIP+
**CHD6**	1.9366665	3.09813E-11	F4: DP_HHIP+
**CLIC4**	1.7119726	3.27349E-11	F4: DP_HHIP+
**CPE**	2.7108064	6.12935E-207	F5: RAMP1+
**SFRP1**	4.615651	4.38141E-199	F5: RAMP1+
**APOD**	2.7096035	8.49014E-199	F5: RAMP1+
**PDGFD**	3.1857245	7.65439E-156	F5: RAMP1+
**FGFBP2**	3.8035104	1.8709E-145	F5: RAMP1+
**OLFML2A**	4.2703204	3.81047E-144	F5: RAMP1+
**KIAA1217**	3.9681568	2.65E-137	F5: RAMP1+
**PRKG1**	2.5822122	3.6957E-131	F5: RAMP1+
**PTN**	2.6095266	2.52451E-125	F5: RAMP1+
**SPRY1**	2.1984427	4.17713E-121	F5: RAMP1+
**SPON2**	2.9326475	1.2745E-119	F5: RAMP1+
**CDH11**	2.7445562	1.82039E-117	F5: RAMP1+

**IGFBP2**	4.847574	5.38319E-106	F5: RAMP1+
**ANTXR2**	2.7186363	7.94525E-101	F5: RAMP1+
**HPSE2**	3.1372588	4.66891E-100	F5: RAMP1+
**RARRES2**	2.052736	1.29968E-99	F5: RAMP1+
**TIMP3**	1.4811488	2.59479E-97	F5: RAMP1+
**LINC00578**	4.760951	1.65593E-96	F5: RAMP1+
**NEGR1**	1.8912281	3.78347E-95	F5: RAMP1+
**IGF1**	3.2668061	1.65885E-94	F5: RAMP1+
**KIF13A**	2.230775	7.78264E-94	F5: RAMP1+
**EDNRA**	3.6458738	3.18633E-93	F5: RAMP1+
**COL15A1**	2.2589781	4.48358E-93	F5: RAMP1+
**PTPRG**	1.891112	4.06568E-91	F5: RAMP1+
**ADAMTS9-/**	2.4436026	9.66977E-90	F5: RAMP1+
**TCF7L2**	1.78483	2.52497E-89	F5: RAMP1+
**SPON1**	2.5105956	8.66219E-89	F5: RAMP1+
**SLC22A3**	3.9715083	1.43554E-88	F5: RAMP1+
**LAMA2**	1.832982	1.68027E-84	F5: RAMP1+
**RORA**	1.7188562	5.87722E-83	F5: RAMP1+
**PRSS23**	2.674041	2.378E-80	F5: RAMP1+
**A2M**	4.6642222	1.30239E-77	F5: RAMP1+
**ZFHX3**	2.297787	6.19899E-76	F5: RAMP1+
**MMP16**	3.2055914	5.93199E-75	F5: RAMP1+
**PLCB1**	2.821247	8.5137E-75	F5: RAMP1+
**ITM2A**	2.3339674	9.84856E-74	F5: RAMP1+
**CALD1**	1.1501491	1.08669E-73	F5: RAMP1+
**TSHZ2**	2.2653224	4.22216E-73	F5: RAMP1+
**PLSCR4**	1.8958265	9.74635E-72	F5: RAMP1+
**PTPN13**	2.1788359	9.07027E-71	F5: RAMP1+
**SCN7A**	4.072284	4.01691E-70	F5: RAMP1+
**NECAB1**	4.9422064	2.11682E-69	F5: RAMP1+
**LDB2**	2.0174382	3.8911E-68	F5: RAMP1+
**LAMC3**	2.0715022	5.6144E-67	F5: RAMP1+
**TXNIP**	1.672203	4.01256E-66	F5: RAMP1+
**FMNL2**	1.9569192	4.96673E-66	F5: RAMP1+

**RAMP1**	4.3244467	8.9651E-64	F5: RAMP1+
**TNRC6C**	2.0470035	1.97512E-63	F5: RAMP1+
**AL590807.1**	5.6660147	3.0047E-63	F5: RAMP1+
**BX284613.2**	4.1893487	6.68425E-63	F5: RAMP1+
**TBX15**	2.173059	6.74542E-61	F5: RAMP1+
**FTX**	1.7771686	2.36828E-60	F5: RAMP1+
**UACA**	2.2134902	2.28673E-59	F5: RAMP1+
**SEPTIN11**	1.5403756	1.12659E-58	F5: RAMP1+
**SIPA1L1**	2.6847064	1.53681E-58	F5: RAMP1+
**FBXL7**	1.6962965	5.90387E-58	F5: RAMP1+
**DOK6**	2.6043243	2.1244E-57	F5: RAMP1+
**PLEKHA6**	3.3227963	1.75701E-56	F5: RAMP1+
**DIO2**	2.1429198	1.3532E-55	F5: RAMP1+
**COL21A1**	2.4927018	1.7027E-55	F5: RAMP1+
**LTBP2**	3.0046604	1.56523E-54	F5: RAMP1+
**NRP1**	1.7449331	1.73929E-54	F5: RAMP1+
**COLEC12**	1.8485513	1.8267E-54	F5: RAMP1+
**CBLB**	1.7093624	2.12089E-54	F5: RAMP1+
**SLIT2**	1.4540563	2.87724E-54	F5: RAMP1+
**SEMA5A**	2.5734386	4.45631E-54	F5: RAMP1+
**N4BP2L2**	1.3797768	5.88075E-54	F5: RAMP1+
**ALX4**	4.7541523	6.98916E-54	F5: RAMP1+
**ACVR2A**	2.2415154	1.14135E-53	F5: RAMP1+
**SAMD5**	4.187462	1.55828E-53	F5: RAMP1+
**DLC1**	1.268169	6.23765E-53	F5: RAMP1+
**TEAD1**	1.744384	8.55611E-53	F5: RAMP1+
**CHN1**	3.1937191	6.08341E-52	F5: RAMP1+
**LRP1**	1.1457659	6.24463E-52	F5: RAMP1+
**CNTN1**	1.8046492	1.91172E-51	F5: RAMP1+
**NTRK3**	3.757165	3.8186E-51	F5: RAMP1+
**PIK3R1**	1.4366982	3.57721E-51	F5: RAMP1+
**GAREM1**	2.4636927	4.73072E-51	F5: RAMP1+
**FAM20A**	2.5044262	7.24624E-51	F5: RAMP1+
**PTPRK**	1.8808511	7.68229E-51	F5: RAMP1+

**LINC01091**	1.8952498	9.7145E-51	F5: RAMP1+
**MAF**	1.7513603	1.07013E-50	F5: RAMP1+
**AC005064.1**	6.080785	3.03289E-50	F5: RAMP1+
**ITGA9**	2.7165067	7.1802E-50	F5: RAMP1+
**GPM6B**	3.0366073	1.60744E-49	F5: RAMP1+
**LINC00486**	1.8309982	3.09275E-49	F5: RAMP1+
**LDLRAD4**	1.838149	4.47477E-49	F5: RAMP1+
**PID1**	1.3489625	4.81695E-49	F5: RAMP1+
**PPFIBP1**	1.630906	4.39786E-48	F5: RAMP1+
**PLEKHH2**	1.5573262	1.31193E-47	F5: RAMP1+
**PRRX1**	1.1136009	2.29755E-47	F5: RAMP1+
**ITM2B**	0.7897295	2.8344E-47	F5: RAMP1+
**SH3BP5**	1.4925358	2.41622E-46	F5: RAMP1+
**PLCL2**	2.0389192	5.04734E-46	F5: RAMP1+
**DKK 3.00**	1.6826997	6.40057E-46	F5: RAMP1+
**EBF1**	1.3310238	1.02877E-45	F5: RAMP1+
**CASC15**	3.0736978	2.31588E-45	F5: RAMP1+
**TCF4**	1.0362293	2.87809E-45	F5: RAMP1+
**FMO2**	4.968974	9.21183E-45	F5: RAMP1+
**ABCA6**	1.6945636	8.7985E-45	F5: RAMP1+
**IGFBP7**	1.9019275	2.38926E-247	F5: NGFR+
**TM4SF1**	7.6524873	1.60996E-233	F5: NGFR+
**PTGDS**	3.6143024	1.7867E-229	F5: NGFR+
**NR2F2**	3.9681187	8.73202E-216	F5: NGFR+
**CYP1B1**	3.3529193	1.3243E-211	F5: NGFR+
**SPARCL1**	3.2063544	9.74905E-200	F5: NGFR+
**TAGLN**	5.3336554	7.40694E-196	F5: NGFR+
**EBF2**	7.513187	3.8973E-180	F5: NGFR+
**APOD**	3.0171087	2.81992E-168	F5: NGFR+
**LTBP4**	1.8993534	2.18625E-168	F5: NGFR+
**BNC2**	4.280805	1.02481E-161	F5: NGFR+
**ECRG4**	6.0153303	1.52554E-149	F5: NGFR+
**NRP2**	4.3127604	1.0493E-148	F5: NGFR+
**SLC22A3**	4.5516376	7.31068E-147	F5: NGFR+

**IFI27**	2.7252092	9.68901E-141	F5: NGFR+
**TENM2**	5.5621862	3.32533E-140	F5: NGFR+
**TFPI**	2.2832654	4.96156E-137	F5: NGFR+
**ITM2B**	1.2536176	5.82564E-136	F5: NGFR+
**FLRT2**	3.473358	1.37962E-133	F5: NGFR+
**SCN7A**	4.7694683	5.08189E-133	F5: NGFR+
**NGFR**	5.878213	7.03401E-131	F5: NGFR+
**DKK 3.00**	2.3146317	1.22011E-130	F5: NGFR+
**MEOX2**	2.7953563	1.34472E-127	F5: NGFR+
**ABCA8**	2.4415562	3.40988E-127	F5: NGFR+
**CSRP1**	2.8237462	1.12556E-126	F5: NGFR+
**MRAS**	3.9246001	2.20191E-122	F5: NGFR+
**CLDN1**	8.490206	1.88246E-121	F5: NGFR+
**SFRP4**	4.1691275	5.12651E-121	F5: NGFR+
**ITGA6**	7.560823	6.54317E-119	F5: NGFR+
**VIT**	2.5948758	1.5579E-116	F5: NGFR+
**SDCBP**	1.5383112	8.10374E-114	F5: NGFR+
**KLF5**	3.1601741	2.41519E-112	F5: NGFR+
**NLGN1**	3.3095605	2.86573E-112	F5: NGFR+
**NR2F2-AS1**	4.207209	9.15319E-111	F5: NGFR+
**SBSPON**	5.511517	8.06876E-110	F5: NGFR+
**EGFR**	1.9757911	1.04358E-108	F5: NGFR+
**CAV2**	2.4813037	8.05747E-108	F5: NGFR+
**TGFBI**	2.5937345	1.16898E-107	F5: NGFR+
**LUM**	1.5309803	4.6119E-107	F5: NGFR+
**CNN3**	1.5186801	1.21135E-104	F5: NGFR+
**P2RY14**	4.7438703	3.7636E-104	F5: NGFR+
**CNTN4**	3.5432212	1.63418E-103	F5: NGFR+
**SYNE2**	2.762469	3.13611E-100	F5: NGFR+
**DAAM1**	2.7701826	2.45995E-99	F5: NGFR+
**A2M**	4.4753575	2.67499E-96	F5: NGFR+
**EPS8**	2.1906276	8.17493E-96	F5: NGFR+
**AKAP12**	1.8226304	1.84323E-93	F5: NGFR+
**MTSS1**	2.2536201	3.32637E-92	F5: NGFR+

**PLEKHA4**	2.3497498	6.73944E-92	F5: NGFR+
**JAM3**	2.1438782	2.67826E-90	F5: NGFR+
**AC116345.1**	6.6172047	7.23856E-90	F5: NGFR+
**SLC2A1**	5.3293796	7.58477E-90	F5: NGFR+
**PDZRN4**	4.3140216	8.1172E-90	F5: NGFR+
**GPC6**	3.862742	1.41422E-88	F5: NGFR+
**ARHGAP10**	1.8346773	1.85359E-88	F5: NGFR+
**FOXD1**	3.6171975	5.42868E-88	F5: NGFR+
**BMPR1B**	5.020911	2.0291E-87	F5: NGFR+
**ARHGEF28**	6.031961	1.23122E-86	F5: NGFR+
**ARHGAP42**	2.9147663	2.4199E-86	F5: NGFR+
**RGS6**	3.1515415	6.58598E-86	F5: NGFR+
**ANGPTL7**	6.6366453	7.79281E-84	F5: NGFR+
**CAV1**	1.3053923	8.22519E-84	F5: NGFR+
**ITGB4**	5.5887027	5.54496E-83	F5: NGFR+
**RBMS3**	1.4292278	8.93806E-82	F5: NGFR+
**PEAR1**	5.578268	1.831E-81	F5: NGFR+
**MATN2**	2.2189198	3.58193E-81	F5: NGFR+
**MCTP1**	6.1464586	1.1158E-80	F5: NGFR+
**OAF**	2.2417116	1.75278E-80	F5: NGFR+
**CDO1**	3.233971	3.46505E-80	F5: NGFR+
**CDH19**	9.026956	2.30585E-79	F5: NGFR+
**SPTBN1**	1.3150477	1.67979E-79	F5: NGFR+
**UBE2E2**	1.6825596	2.6065E-79	F5: NGFR+
**UACA**	2.1146486	4.42709E-79	F5: NGFR+
**TJP1**	1.9548823	1.33141E-77	F5: NGFR+
**RARRES2**	1.4566232	2.39186E-77	F5: NGFR+
**GAB1**	2.2903576	6.90601E-77	F5: NGFR+
**NFE2L2**	1.2993903	9.53212E-77	F5: NGFR+
**LINC01197**	6.0044675	1.97351E-76	F5: NGFR+
**HRH1**	3.584936	5.32515E-75	F5: NGFR+
**COL8A1**	4.0849347	1.18821E-73	F5: NGFR+
**KIAA1217**	2.444594	1.97321E-73	F5: NGFR+
**ADAMTS9-/**	1.8316848	4.60212E-73	F5: NGFR+

**STMN1**	2.1997616	5.08314E-73	F5: NGFR+
**AFDN**	2.4695845	7.22221E-73	F5: NGFR+
**GPC3**	1.8819532	9.20215E-73	F5: NGFR+
**FRMD4B**	2.8770478	1.14598E-72	F5: NGFR+
**EZR**	2.9092255	5.91344E-72	F5: NGFR+
**SREBF2**	2.8323107	1.42118E-71	F5: NGFR+
**SOX5**	2.9122872	1.10426E-70	F5: NGFR+
**ITM2A**	1.8191636	1.70828E-70	F5: NGFR+
**PTCH2**	3.012389	2.70986E-70	F5: NGFR+
**TXN**	1.0039232	1.05463E-69	F5: NGFR+
**CAVIN2**	5.9464126	3.01211E-68	F5: NGFR+
**TXNIP**	1.4852674	2.13703E-67	F5: NGFR+
**TNNC1**	5.01287	8.21286E-67	F5: NGFR+
**BTG2**	1.774419	1.14664E-66	F5: NGFR+
**PALMD**	4.13833	2.40906E-66	F5: NGFR+
**PLK2**	2.4901223	1.03036E-64	F5: NGFR+
**CALD1**	0.9079677	3.62238E-64	F5: NGFR+
**ETV1**	3.5104527	5.50549E-64	F5: NGFR+

Supplementary Table 3

Supplementary Table 3 | Differentially expressed genes for diseased/lesional fibroblasts.

SupplementaryTable3

Gene	logFC	p-value	Group
APOE	5.1067867	0E+00	F3: FRC-like
CXCL12	4.576376	0E+00	F3: FRC-like
IGFBP7	2.4296026	0E+00	F3: FRC-like
CXCL14	3.6259897	0E+00	F3: FRC-like
C3	4.124011	0E+00	F3: FRC-like
SOD3	2.6876626	0E+00	F3: FRC-like
CCL19	6.143259	0E+00	F3: FRC-like
CFD	3.1460001	0E+00	F3: FRC-like
RPS4X	1.6930614	0E+00	F3: FRC-like
PTGDS	3.3595457	0E+00	F3: FRC-like
SERPING1	1.9744971	0E+00	F3: FRC-like
APOD	3.0829244	0E+00	F3: FRC-like
RPL10	1.6851487	0E+00	F3: FRC-like
CFH	2.141725	0E+00	F3: FRC-like
C1S	1.7139579	0E+00	F3: FRC-like
ZFP36L2	2.0755208	0E+00	F3: FRC-like
HLA-B	1.7841635	0E+00	F3: FRC-like
NFIA	2.6087303	0E+00	F3: FRC-like
EFEMP1	2.4361463	0E+00	F3: FRC-like
RPL12	1.1900324	0E+00	F3: FRC-like
SELENOP	2.1187491	0E+00	F3: FRC-like
GGT5	2.824583	0E+00	F3: FRC-like
RPL36A	1.6982213	0E+00	F3: FRC-like
IFITM3	1.3066607	0E+00	F3: FRC-like
CCDC80	1.4023626	0E+00	F3: FRC-like
ABI3BP	2.0801103	0E+00	F3: FRC-like
CST3	1.329478	0E+00	F3: FRC-like
EEF1A1	1.229287	0E+00	F3: FRC-like
TNFSF13B	4.2528353	0E+00	F3: FRC-like
SNHG8	1.7935537	0E+00	F3: FRC-like
RBP5	4.185686	0E+00	F3: FRC-like
SOCS3	2.183965	0E+00	F3: FRC-like
RPL37	1.0356717	0E+00	F3: FRC-like
CCL2	2.5804255	0E+00	F3: FRC-like
ZFP36L1	1.4109243	0E+00	F3: FRC-like
PNRC1	1.5689389	0E+00	F3: FRC-like
RPL7A	1.0511293	0E+00	F3: FRC-like
FGL2	2.8343484	0E+00	F3: FRC-like
RPL22	1.467029	0E+00	F3: FRC-like
BTG1	1.7201961	0E+00	F3: FRC-like
MT-ND3	1.7332804	0E+00	F3: FRC-like
RPS8	0.88268596	0E+00	F3: FRC-like
B2M	0.82725877	0E+00	F3: FRC-like
SNED1	2.4378529	0E+00	F3: FRC-like
ADH1B	3.2496774	0E+00	F3: FRC-like
RPL39	2.1253448	0E+00	F3: FRC-like
TMEM176A	1.8793625	0E+00	F3: FRC-like
ABCA8	3.489617	0E+00	F3: FRC-like
RPS23	0.8500003	0E+00	F3: FRC-like
FGF7	2.466828	0E+00	F3: FRC-like
CLU	2.126194	0E+00	F3: FRC-like

**RPL32**	0.8658211	0E+00	F3: FRC-like
**TMEM176B**	1.6552441	0E+00	F3: FRC-like
**CYP7B1**	1.7531179	0E+00	F3: FRC-like
**VCAN**	1.391159	0E+00	F3: FRC-like
**MT-ND1**	1.4436312	0E+00	F3: FRC-like
**RARRES2**	1.4983369	0E+00	F3: FRC-like
**TFPI**	2.1359746	0E+00	F3: FRC-like
**IFITM2**	1.3171508	0E+00	F3: FRC-like
**RPL34**	0.83089477	0E+00	F3: FRC-like
**RPL26**	0.8093146	0E+00	F3: FRC-like
**CTSH**	2.1901522	0E+00	F3: FRC-like
**GSN**	1.4904473	0E+00	F3: FRC-like
**HLA-A**	1.1075895	0E+00	F3: FRC-like
**HSPA1A**	1.6382103	0E+00	F3: FRC-like
**RPS27**	0.98138434	0E+00	F3: FRC-like
**CD74**	2.7438998	0E+00	F3: FRC-like
**MT2A**	1.7172005	0E+00	F3: FRC-like
**SOD2**	1.4603517	0E+00	F3: FRC-like
**ZFP36**	1.6574479	0E+00	F3: FRC-like
**RPS28**	0.9209929	0E+00	F3: FRC-like
**CEBPD**	1.5496979	0E+00	F3: FRC-like
**CYP1B1**	2.7370863	0E+00	F3: FRC-like
**RPL35A**	0.80691093	0E+00	F3: FRC-like
**DCLK1**	1.6987743	0E+00	F3: FRC-like
**RPL35**	0.9041439	0E+00	F3: FRC-like
**CYGB**	2.487532	0E+00	F3: FRC-like
**COL15A1**	1.7110229	0E+00	F3: FRC-like
**LGALS3**	0.9222501	0E+00	F3: FRC-like
**RPL41**	0.82604337	0E+00	F3: FRC-like
**TNFSF10**	2.3338177	0E+00	F3: FRC-like
**TPT1**	0.79245055	0E+00	F3: FRC-like
**RPS13**	0.79163957	0E+00	F3: FRC-like
**LAMA4**	1.3829535	0E+00	F3: FRC-like
**IL11RA**	1.7808115	0E+00	F3: FRC-like
**MGP**	1.3582753	0E+00	F3: FRC-like
**APOC1**	4.2021947	0E+00	F3: FRC-like
**NFIB**	1.800682	0E+00	F3: FRC-like
**NOP53**	1.2626119	0E+00	F3: FRC-like
**PPA1**	1.295371	0E+00	F3: FRC-like
**RPS12**	0.72982574	0E+00	F3: FRC-like
**TXNIP**	1.7042633	0E+00	F3: FRC-like
**MT-CO2**	1.1712618	0E+00	F3: FRC-like
**RPL29**	0.80938905	0E+00	F3: FRC-like
**CH25H**	2.3984685	0E+00	F3: FRC-like
**TMSB4X**	0.9549993	0E+00	F3: FRC-like
**EBF1**	1.6435168	0E+00	F3: FRC-like
**MT-CYB**	1.1687857	0E+00	F3: FRC-like
**RPL14**	0.74294573	0E+00	F3: FRC-like
**RPS24**	0.64145833	0E+00	F3: FRC-like
**APCDD1**	4.323153	0E+00	F1: Superficial
**TWIST2**	2.5716825	0E+00	F1: Superficial
**PLPP3**	2.4345284	0E+00	F1: Superficial
**COL18A1**	2.4910228	0E+00	F1: Superficial

**F13A1**	4.6078205	0E+00	F1: Superficial
**CD9**	1.9829272	0E+00	F1: Superficial
**IGFBP4**	2.0483513	0E+00	F1: Superficial
**PMP22**	1.783317	0E+00	F1: Superficial
**ZFP36L1**	1.8787786	0E+00	F1: Superficial
**CEBPD**	2.356711	0E+00	F1: Superficial
**ZFP36L2**	2.191186	0E+00	F1: Superficial
**CEBPB**	2.2817197	0E+00	F1: Superficial
**SOD3**	2.223636	0E+00	F1: Superficial
**C1S**	1.5569468	0E+00	F1: Superficial
**SERPING1**	1.6888565	0E+00	F1: Superficial
**MT-CO2**	1.7758929	0E+00	F1: Superficial
**C1R**	1.6417977	0E+00	F1: Superficial
**GLUL**	2.0528276	0E+00	F1: Superficial
**CYP26B1**	3.4968753	0E+00	F1: Superficial
**PNRC1**	1.8160245	0E+00	F1: Superficial
**MT-ND3**	2.0322149	0E+00	F1: Superficial
**FBLN2**	1.685356	0E+00	F1: Superficial
**HLA-B**	1.6391743	0E+00	F1: Superficial
**SELENOP**	2.020506	0E+00	F1: Superficial
**PTGDS**	2.7591386	0E+00	F1: Superficial
**RPL10**	1.494345	0E+00	F1: Superficial
**SPRY1**	2.449008	0E+00	F1: Superficial
**EEF1A1**	1.1700206	0E+00	F1: Superficial
**MT-ND1**	1.6775846	0E+00	F1: Superficial
**MT-CYB**	1.6312568	0E+00	F1: Superficial
**RPS4X**	1.2779554	0E+00	F1: Superficial
**HLA-A**	1.3236567	0E+00	F1: Superficial
**PAPPA**	2.618173	0E+00	F1: Superficial
**ROBO2**	2.3233554	0E+00	F1: Superficial
**MT-ND2**	1.543447	0E+00	F1: Superficial
**NKD2**	3.192101	0E+00	F1: Superficial
**MT-ATP6**	1.6961379	0E+00	F1: Superficial
**COL23A1**	3.4213758	0E+00	F1: Superficial
**CFD**	2.024292	0E+00	F1: Superficial
**PGRMC1**	1.6531785	0E+00	F1: Superficial
**AHR**	1.6057773	0E+00	F1: Superficial
**SFRP2**	1.6288083	0E+00	F1: Superficial
**F10**	2.342929	0E+00	F1: Superficial
**SUMO2**	1.9174938	0E+00	F1: Superficial
**MT-CO3**	1.5663284	0E+00	F1: Superficial
**B2M**	0.8555882	0E+00	F1: Superficial
**ANPEP**	2.0618784	0E+00	F1: Superficial
**DCN**	1.0950426	0E+00	F1: Superficial
**RPL22**	1.3118671	0E+00	F1: Superficial
**DUSP1**	1.4597999	0E+00	F1: Superficial
**DIO2**	2.279153	0E+00	F1: Superficial
**RPL36A**	1.3157895	0E+00	F1: Superficial
**RPL39**	1.9049826	0E+00	F1: Superficial
**HLA-E**	1.3261088	0E+00	F1: Superficial
**SOD2**	1.6695116	0E+00	F1: Superficial
**TSC22D3**	1.829453	0E+00	F1: Superficial
**COL6A5**	3.0317192	0E+00	F1: Superficial

**LEPR**	3.4580116	0E+00	F1: Superficial
**APOD**	1.8082026	0E+00	F1: Superficial
**TCF4**	1.2341632	0E+00	F1: Superficial
**CAV1**	1.2904688	0E+00	F1: Superficial
**THBS1**	1.9968832	0E+00	F1: Superficial
**ZBTB20**	1.3332394	0E+00	F1: Superficial
**H3F3A**	2.026882	0E+00	F1: Superficial
**ADIRF**	1.3240945	0E+00	F1: Superficial
**MT-CO1**	1.2128273	0E+00	F1: Superficial
**COL4A1**	1.7746089	0E+00	F1: Superficial
**TWIST1**	1.482495	0E+00	F1: Superficial
**IL6ST**	1.3975673	0E+00	F1: Superficial
**IFI27**	1.948659	0E+00	F1: Superficial
**PIK3R1**	1.5800555	0E+00	F1: Superficial
**AHNAK**	1.1413997	0E+00	F1: Superficial
**MTRNR2L12**	2.0197144	0E+00	F1: Superficial
**PRRX2**	1.5612884	0E+00	F1: Superficial
**MT-ND4L**	1.5725341	0E+00	F1: Superficial
**C1orf198**	1.7224232	0E+00	F1: Superficial
**LAMC3**	3.0713944	0E+00	F1: Superficial
**TNXB**	1.9601775	0E+00	F1: Superficial
**ARRDC3**	1.7272041	0E+00	F1: Superficial
**STC2**	3.7835076	0E+00	F1: Superficial
**NCKAP5**	2.7417917	0E+00	F1: Superficial
**DUSP4**	3.0852478	0E+00	F1: Superficial
**CHCHD10**	1.6343426	0E+00	F1: Superficial
**RPS28**	0.86764973	0E+00	F1: Superficial
**LY6E**	1.2250836	0E+00	F1: Superficial
**PID1**	1.8453714	0E+00	F1: Superficial
**RASD1**	1.9641663	0E+00	F1: Superficial
**HSPA1A**	1.4989344	0E+00	F1: Superficial
**PTGS2**	2.5647535	0E+00	F1: Superficial
**IFITM3**	0.9554633	0E+00	F1: Superficial
**PDGFRA**	1.3376889	0E+00	F1: Superficial
**FST**	2.1590345	0E+00	F1: Superficial
**RSPO1**	3.509917	0E+00	F1: Superficial
**SVIL**	1.4791844	0E+00	F1: Superficial
**RPL12**	0.81707567	0E+00	F1: Superficial
**ARHGAP29**	1.8570001	0E+00	F1: Superficial
**ITIH5**	1.9623355	0E+00	F1: Superficial
**ZFAND5**	1.3661475	0E+00	F1: Superficial
**RND3**	1.2465212	0E+00	F1: Superficial
**GPNMB**	1.0818149	0E+00	F1: Superficial
**INHBA**	2.6958618	0E+00	F6: Inflammatory myofibroblast
**PRSS23**	1.8807056	0E+00	F6: Inflammatory myofibroblast
**COL4A1**	1.9616207	0E+00	F6: Inflammatory myofibroblast
**PRKG1**	1.7724618	0E+00	F6: Inflammatory myofibroblast
**COL5A3**	2.1116602	0E+00	F6: Inflammatory myofibroblast
**COL4A2**	1.688526	0E+00	F6: Inflammatory myofibroblast
**SAT1**	1.8636863	0E+00	F6: Inflammatory myofibroblast
**HLA-B**	1.3460884	0E+00	F6: Inflammatory myofibroblast
**SGIP1**	2.388894	0E+00	F6: Inflammatory myofibroblast
**GPM6B**	2.9743686	0E+00	F6: Inflammatory myofibroblast

**LUM**	1.1958944	0E+00	F6: Inflammatory myofibroblast
**WNT5A**	2.6485476	0E+00	F6: Inflammatory myofibroblast
**CSGALNACT**	2.2000995	0E+00	F6: Inflammatory myofibroblast
**MME**	2.5109093	0E+00	F6: Inflammatory myofibroblast
**BGN**	1.1580427	0E+00	F6: Inflammatory myofibroblast
**COL7A1**	2.4928918	0E+00	F6: Inflammatory myofibroblast
**RUNX2**	2.0250444	0E+00	F6: Inflammatory myofibroblast
**PTPRG**	1.4914118	0E+00	F6: Inflammatory myofibroblast
**IFITM3**	0.97576284	0E+00	F6: Inflammatory myofibroblast
**GLIS3**	1.6590002	0E+00	F6: Inflammatory myofibroblast
**GGT5**	1.582822	0E+00	F6: Inflammatory myofibroblast
**TIMP1**	1.1588503	0E+00	F6: Inflammatory myofibroblast
**HES4**	1.8838204	0E+00	F6: Inflammatory myofibroblast
**KIF26B**	1.9877422	0E+00	F6: Inflammatory myofibroblast
**MMP1**	4.55709	0E+00	F6: Inflammatory myofibroblast
**SOX5**	3.1383862	0E+00	F6: Inflammatory myofibroblast
**CPE**	1.5815095	0E+00	F6: Inflammatory myofibroblast
**FRMD4A**	2.1087987	0E+00	F6: Inflammatory myofibroblast
**MAP3K5**	2.6026967	0E+00	F6: Inflammatory myofibroblast
**PRRX1**	0.827302	0E+00	F6: Inflammatory myofibroblast
**CHN1**	1.9016978	0E+00	F6: Inflammatory myofibroblast
**TSPAN9**	1.6981586	0E+00	F6: Inflammatory myofibroblast
**EPAS1**	1.5031518	0E+00	F6: Inflammatory myofibroblast
**TENM4**	2.393796	0E+00	F6: Inflammatory myofibroblast
**SEMA5A**	1.7676203	0E+00	F6: Inflammatory myofibroblast
**SPHK1**	1.5553426	0E+00	F6: Inflammatory myofibroblast
**COL27A1**	1.5554938	0E+00	F6: Inflammatory myofibroblast
**EPSTI1**	2.0466168	0E+00	F6: Inflammatory myofibroblast
**STARD13**	1.6697224	0E+00	F6: Inflammatory myofibroblast
**SULF1**	1.8295788	0E+00	F6: Inflammatory myofibroblast
**GUCY1A1**	1.5084609	0E+00	F6: Inflammatory myofibroblast
**PMEPA1**	1.1549543	0E+00	F6: Inflammatory myofibroblast
**DPYD**	1.386184	0E+00	F6: Inflammatory myofibroblast
**TSPAN5**	1.5756277	0E+00	F6: Inflammatory myofibroblast
**CHST11**	1.6004336	1.45004E-309	F6: Inflammatory myofibroblast
**PITPNC1**	1.8757064	6.87493E-304	F6: Inflammatory myofibroblast
**LAMB1**	1.2197872	1.14257E-304	F6: Inflammatory myofibroblast
**TDO2**	3.7714515	4.0266E-298	F6: Inflammatory myofibroblast
**CD82**	1.5468645	8.77706E-290	F6: Inflammatory myofibroblast
**C4orf48**	1.3856821	2.18406E-285	F6: Inflammatory myofibroblast
**PRDM1**	1.8415577	4.77907E-279	F6: Inflammatory myofibroblast
**ASAP1**	1.0475726	2.11106E-274	F6: Inflammatory myofibroblast
**PLD1**	1.9898561	3.11591E-270	F6: Inflammatory myofibroblast
**TNFRSF21**	2.2570424	8.25367E-270	F6: Inflammatory myofibroblast
**CA12**	2.255897	6.41572E-269	F6: Inflammatory myofibroblast
**AOPEP**	1.0366243	3.70105E-268	F6: Inflammatory myofibroblast
**SH3RF3**	1.6541066	5.29114E-265	F6: Inflammatory myofibroblast
**TRPS1**	1.1423122	6.00698E-265	F6: Inflammatory myofibroblast
**MMP3**	5.1715403	2.70774E-262	F6: Inflammatory myofibroblast
**FP236383.3**	1.5416298	1.33963E-263	F6: Inflammatory myofibroblast
**DIO2**	1.4376601	4.62947E-263	F6: Inflammatory myofibroblast
**UNC5B**	1.665223	3.0857E-261	F6: Inflammatory myofibroblast
**PLEKHA5**	1.2854282	7.60726E-259	F6: Inflammatory myofibroblast

**PTPRE**	2.1124017	4.57257E-258	F6: Inflammatory myofibroblast
**CYTOR**	1.0597994	6.21155E-259	F6: Inflammatory myofibroblast
**BICC1**	0.9868387	3.8375E-259	F6: Inflammatory myofibroblast
**BMP1**	1.1288034	4.28462E-255	F6: Inflammatory myofibroblast
**MSC-AS1**	1.5576873	1.89245E-254	F6: Inflammatory myofibroblast
**GFPT2**	1.5672665	4.72929E-254	F6: Inflammatory myofibroblast
**PRR16**	1.6988724	8.70665E-253	F6: Inflammatory myofibroblast
**CCN4**	1.6157064	8.18361E-251	F6: Inflammatory myofibroblast
**ANKRD28**	1.2427306	1.93014E-249	F6: Inflammatory myofibroblast
**ST5**	1.3362043	3.82718E-247	F6: Inflammatory myofibroblast
**ELOVL5**	1.3821392	2.82965E-246	F6: Inflammatory myofibroblast
**PDE3A**	2.5298047	1.98248E-245	F6: Inflammatory myofibroblast
**CBLB**	1.299563	3.11929E-244	F6: Inflammatory myofibroblast
**COL6A3**	0.6547348	4.29033E-245	F6: Inflammatory myofibroblast
**ADAMTS2**	0.91728896	5.38393E-244	F6: Inflammatory myofibroblast
**PHLDA1**	1.1702586	1.18702E-238	F6: Inflammatory myofibroblast
**HLA-A**	0.7762304	8.90556E-239	F6: Inflammatory myofibroblast
**ARHGAP24**	1.201702	5.40948E-238	F6: Inflammatory myofibroblast
**EDNRA**	1.5635837	1.44003E-237	F6: Inflammatory myofibroblast
**LMCD1**	1.5772519	3.04597E-236	F6: Inflammatory myofibroblast
**MIR4435-2H**	1.2124777	2.43539E-235	F6: Inflammatory myofibroblast
**GRAMD1A**	1.6693207	8.9493E-235	F6: Inflammatory myofibroblast
**ETV6**	1.1696799	1.03896E-230	F6: Inflammatory myofibroblast
**LY6E**	0.86272395	9.49154E-227	F6: Inflammatory myofibroblast
**NOTCH3**	1.939418	1.167E-225	F6: Inflammatory myofibroblast
**PSME2**	1.0024394	3.00705E-224	F6: Inflammatory myofibroblast
**F3**	2.4554906	2.81485E-223	F6: Inflammatory myofibroblast
**PTMS**	0.768319	5.00126E-224	F6: Inflammatory myofibroblast
**ITGA1**	1.5597681	9.24297E-223	F6: Inflammatory myofibroblast
**ISG15**	1.3648654	5.87803E-222	F6: Inflammatory myofibroblast
**PDGFRB**	0.8301962	1.53522E-222	F6: Inflammatory myofibroblast
**HLA-F**	1.2691072	5.53264E-220	F6: Inflammatory myofibroblast
**RBPMS**	1.2313572	1.13899E-218	F6: Inflammatory myofibroblast
**TMEM165**	0.93980503	9.6931E-219	F6: Inflammatory myofibroblast
**CACNA1C**	1.1128141	1.00202E-217	F6: Inflammatory myofibroblast
**COL5A1**	0.7417554	5.19657E-218	F6: Inflammatory myofibroblast
**PAG1**	1.5866561	4.12175E-214	F6: Inflammatory myofibroblast
**COL1A1**	2.2306933	0E+00	F7: Myofibroblast
**COL1A2**	2.2047234	0E+00	F7: Myofibroblast
**SPARC**	1.99527	0E+00	F7: Myofibroblast
**COL3A1**	1.8008479	0E+00	F7: Myofibroblast
**CTHRC1**	2.3359032	0E+00	F7: Myofibroblast
**COL5A1**	1.8954477	0E+00	F7: Myofibroblast
**PRDX4**	2.5311801	0E+00	F7: Myofibroblast
**OGN**	3.4726238	0E+00	F7: Myofibroblast
**ASPN**	2.4268184	0E+00	F7: Myofibroblast
**COL14A1**	1.7988192	0E+00	F7: Myofibroblast
**COL5A2**	1.284501	0E+00	F7: Myofibroblast
**SFRP2**	1.9098601	0E+00	F7: Myofibroblast
**GXYLT2**	2.1567233	0E+00	F7: Myofibroblast
**PRSS23**	2.2808368	0E+00	F7: Myofibroblast
**ADAM12**	2.052579	0E+00	F7: Myofibroblast
**HTRA1**	1.4398735	0E+00	F7: Myofibroblast

**MDK**	1.8253778	0E+00	F7: Myofibroblast
**COPZ2**	1.8701892	0E+00	F7: Myofibroblast
**MFAP5**	2.7368667	0E+00	F7: Myofibroblast
**MIR99AHG**	2.0284996	0E+00	F7: Myofibroblast
**PPIC**	1.4469078	0E+00	F7: Myofibroblast
**LUM**	1.2852353	0E+00	F7: Myofibroblast
**MMP23B**	2.0597858	0E+00	F7: Myofibroblast
**CCDC80**	1.3920984	0E+00	F7: Myofibroblast
**FAP**	1.5239515	0E+00	F7: Myofibroblast
**MARCKS**	1.3815775	0E+00	F7: Myofibroblast
**COL12A1**	1.4962387	0E+00	F7: Myofibroblast
**CENPP**	2.3820546	0E+00	F7: Myofibroblast
**SEPTIN11**	1.5109216	0E+00	F7: Myofibroblast
**COL11A1**	2.8984735	0E+00	F7: Myofibroblast
**PLXDC2**	1.5630836	0E+00	F7: Myofibroblast
**PPP1R14B**	1.9098611	0E+00	F7: Myofibroblast
**KIF26B**	2.49664	0E+00	F7: Myofibroblast
**KRT10**	1.39174	0E+00	F7: Myofibroblast
**LGR4**	2.253887	0E+00	F7: Myofibroblast
**POSTN**	1.4333525	0E+00	F7: Myofibroblast
**FN1**	1.1769091	0E+00	F7: Myofibroblast
**BNC2**	1.7537265	0E+00	F7: Myofibroblast
**ELN**	1.8135302	0E+00	F7: Myofibroblast
**BCKDHB**	2.5661256	0E+00	F7: Myofibroblast
**HMCN1**	1.5159303	0E+00	F7: Myofibroblast
**GOLM1**	1.7217025	0E+00	F7: Myofibroblast
**LOX**	1.4756968	0E+00	F7: Myofibroblast
**SULF2**	1.8486274	0E+00	F7: Myofibroblast
**BGN**	1.198804	0E+00	F7: Myofibroblast
**H19**	4.498432	0E+00	F7: Myofibroblast
**COL8A1**	1.8716991	0E+00	F7: Myofibroblast
**CD99**	1.0099342	0E+00	F7: Myofibroblast
**NREP**	1.436919	0E+00	F7: Myofibroblast
**DPT**	1.1386058	0E+00	F7: Myofibroblast
**SMYD3**	1.676233	0E+00	F7: Myofibroblast
**PTN**	1.501669	0E+00	F7: Myofibroblast
**CERCAM**	1.3579024	0E+00	F7: Myofibroblast
**IGF1**	1.9578431	0E+00	F7: Myofibroblast
**RASAL2**	1.4645798	0E+00	F7: Myofibroblast
**PIEZO2**	2.0730755	0E+00	F7: Myofibroblast
**NRG1**	2.7143037	0E+00	F7: Myofibroblast
**PPIB**	0.8941524	0E+00	F7: Myofibroblast
**IGFBP6**	1.6754459	0E+00	F7: Myofibroblast
**PALLD**	1.1739303	0E+00	F7: Myofibroblast
**CPE**	1.3872732	0E+00	F7: Myofibroblast
**PRKG1**	1.2977244	0E+00	F7: Myofibroblast
**TTC3**	1.0334327	0E+00	F7: Myofibroblast
**ITGBL1**	1.4145417	0E+00	F7: Myofibroblast
**TENM3**	1.764915	0E+00	F7: Myofibroblast
**SGCD**	1.3392756	0E+00	F7: Myofibroblast
**TSPAN13**	2.553722	0E+00	F7: Myofibroblast
**SSR4**	1.005466	0E+00	F7: Myofibroblast
**MFAP4**	1.0664054	0E+00	F7: Myofibroblast

**TXNL4A**	1.4126381	0E+00	F7: Myofibroblast
**CCN2**	1.3483568	0E+00	F7: Myofibroblast
**ARHGAP28**	2.3697536	0E+00	F7: Myofibroblast
**PRICKLE1**	1.6637743	0E+00	F7: Myofibroblast
**PDGFRL**	1.234624	0E+00	F7: Myofibroblast
**METRN**	1.9639505	0E+00	F7: Myofibroblast
**EDIL3**	1.2154009	0E+00	F7: Myofibroblast
**MRPS6**	1.4367664	0E+00	F7: Myofibroblast
**KIAA1217**	1.2963613	0E+00	F7: Myofibroblast
**TIMP1**	0.9388238	0E+00	F7: Myofibroblast
**AUTS2**	1.1628613	0E+00	F7: Myofibroblast
**SULF1**	1.6824774	0E+00	F7: Myofibroblast
**MGP**	1.0375048	0E+00	F7: Myofibroblast
**MXRA5**	1.0695505	0E+00	F7: Myofibroblast
**PDZRN4**	3.2011318	0E+00	F7: Myofibroblast
**FOXP1**	1.078305	0E+00	F7: Myofibroblast
**SEC61G**	0.8433767	0E+00	F7: Myofibroblast
**PCOLCE**	0.76491606	0E+00	F7: Myofibroblast
**OSTC**	0.8781052	0E+00	F7: Myofibroblast
**LINC02544**	2.7890394	0E+00	F7: Myofibroblast
**FBN1**	0.8814653	0E+00	F7: Myofibroblast
**VEGFB**	1.3216959	0E+00	F7: Myofibroblast
**BRI3**	0.9658423	0E+00	F7: Myofibroblast
**JUND**	0.88506216	0E+00	F7: Myofibroblast
**RUNX2**	1.5281537	0E+00	F7: Myofibroblast
**ADAMTS6**	2.6207325	0E+00	F7: Myofibroblast
**STARD4-AS1**	2.4558377	0E+00	F7: Myofibroblast
**PHPT1**	0.89385283	0E+00	F7: Myofibroblast
**PDE1A**	2.1708562	0E+00	F7: Myofibroblast
**C1QTNF6**	1.3843178	0E+00	F7: Myofibroblast
**PTGER3**	1.5859609	0E+00	F7: Myofibroblast
**POSTN**	3.7974658	0E+00	F8: Fascia-like myofibroblast
**SPON2**	3.5428421	0E+00	F8: Fascia-like myofibroblast
**CADM1**	3.7399058	0E+00	F8: Fascia-like myofibroblast
**TGFBI**	3.38161	0E+00	F8: Fascia-like myofibroblast
**FN1**	2.3818934	0E+00	F8: Fascia-like myofibroblast
**CAMK1D**	3.1290152	0E+00	F8: Fascia-like myofibroblast
**TAGLN**	3.7701232	0E+00	F8: Fascia-like myofibroblast
**FABP5**	4.6802645	0E+00	F8: Fascia-like myofibroblast
**RPS17**	2.038429	0E+00	F8: Fascia-like myofibroblast
**SFRP4**	3.5803206	0E+00	F8: Fascia-like myofibroblast
**DKK 3.00**	2.7743852	0E+00	F8: Fascia-like myofibroblast
**RPL13A**	1.7877821	0E+00	F8: Fascia-like myofibroblast
**TPM1**	2.304859	0E+00	F8: Fascia-like myofibroblast
**PDLIM3**	3.9170084	0E+00	F8: Fascia-like myofibroblast
**C1QTNF3**	3.1645162	0E+00	F8: Fascia-like myofibroblast
**EDIL3**	2.6762483	0E+00	F8: Fascia-like myofibroblast
**COL1A1**	1.7105033	0E+00	F8: Fascia-like myofibroblast
**COL5A2**	1.5957056	0E+00	F8: Fascia-like myofibroblast
**PDLIM7**	2.9120247	0E+00	F8: Fascia-like myofibroblast
**DLG1**	2.8377407	0E+00	F8: Fascia-like myofibroblast
**MALAT1**	1.5753797	0E+00	F8: Fascia-like myofibroblast
**SPARCL1**	2.3112514	0E+00	F8: Fascia-like myofibroblast

**LHFPL6**	1.7989981	0E+00	F8: Fascia-like myofibroblast
**RHOC**	1.9936419	0E+00	F8: Fascia-like myofibroblast
**CNN3**	1.7326602	0E+00	F8: Fascia-like myofibroblast
**RPL27A**	1.4517398	0E+00	F8: Fascia-like myofibroblast
**TUBA1A**	2.0158017	0E+00	F8: Fascia-like myofibroblast
**CRABP2**	1.9652675	0E+00	F8: Fascia-like myofibroblast
**COL3A1**	1.4187343	0E+00	F8: Fascia-like myofibroblast
**LRRC17**	3.0686107	0E+00	F8: Fascia-like myofibroblast
**ITGB1**	1.564998	0E+00	F8: Fascia-like myofibroblast
**ASPN**	3.0039992	0E+00	F8: Fascia-like myofibroblast
**P4HA3**	2.9807594	0E+00	F8: Fascia-like myofibroblast
**NIBAN1**	3.2852974	0E+00	F8: Fascia-like myofibroblast
**TUBB2B**	3.2710128	0E+00	F8: Fascia-like myofibroblast
**FOXP1**	2.0690372	0E+00	F8: Fascia-like myofibroblast
**ADAM12**	2.1642318	0E+00	F8: Fascia-like myofibroblast
**TPPP3**	2.8390326	0E+00	F8: Fascia-like myofibroblast
**RPS20**	1.5679573	0E+00	F8: Fascia-like myofibroblast
**CAPZB**	1.5715346	0E+00	F8: Fascia-like myofibroblast
**PDPN**	2.0664663	0E+00	F8: Fascia-like myofibroblast
**PKM**	1.6161861	0E+00	F8: Fascia-like myofibroblast
**TPM4**	1.4161328	0E+00	F8: Fascia-like myofibroblast
**CASC15**	2.8890164	0E+00	F8: Fascia-like myofibroblast
**HIP1**	2.674073	0E+00	F8: Fascia-like myofibroblast
**MYL6**	1.189563	0E+00	F8: Fascia-like myofibroblast
**THBS4**	3.141329	0E+00	F8: Fascia-like myofibroblast
**ACTG1**	1.2392994	0E+00	F8: Fascia-like myofibroblast
**GABARAPL2**	1.7437463	0E+00	F8: Fascia-like myofibroblast
**ACAN**	3.4867	0E+00	F8: Fascia-like myofibroblast
**CAPG**	2.2524354	0E+00	F8: Fascia-like myofibroblast
**CTHRC1**	1.7153925	0E+00	F8: Fascia-like myofibroblast
**ACTA2**	3.170795	0E+00	F8: Fascia-like myofibroblast
**PLAU**	2.717268	0E+00	F8: Fascia-like myofibroblast
**DGKI**	2.9079242	0E+00	F8: Fascia-like myofibroblast
**LGALS1**	1.4059808	0E+00	F8: Fascia-like myofibroblast
**SPAG9**	1.8210143	0E+00	F8: Fascia-like myofibroblast
**TRIO**	1.8690945	0E+00	F8: Fascia-like myofibroblast
**ARL4C**	2.1018853	0E+00	F8: Fascia-like myofibroblast
**ATP1B1**	3.2165797	0E+00	F8: Fascia-like myofibroblast
**CSRP2**	1.9630514	0E+00	F8: Fascia-like myofibroblast
**HTRA1**	1.3662772	0E+00	F8: Fascia-like myofibroblast
**LRRFIP1**	1.739903	0E+00	F8: Fascia-like myofibroblast
**TAGLN2**	1.4626133	0E+00	F8: Fascia-like myofibroblast
**TNFRSF12A**	1.824729	0E+00	F8: Fascia-like myofibroblast
**PLPP4**	2.7088833	0E+00	F8: Fascia-like myofibroblast
**GPC6**	2.1918013	0E+00	F8: Fascia-like myofibroblast
**ARPC2**	1.3665786	0E+00	F8: Fascia-like myofibroblast
**CALD1**	1.1833436	0E+00	F8: Fascia-like myofibroblast
**NCAM1**	3.6327353	0E+00	F8: Fascia-like myofibroblast
**ACTB**	1.2748365	0E+00	F8: Fascia-like myofibroblast
**ENAH**	1.5476233	0E+00	F8: Fascia-like myofibroblast
**CFL1**	1.2400322	0E+00	F8: Fascia-like myofibroblast
**TPM2**	1.6332642	0E+00	F8: Fascia-like myofibroblast
**NBL1**	1.4518698	0E+00	F8: Fascia-like myofibroblast

**ENO1**	1.3985786	0E+00	F8: Fascia-like myofibroblast
**CRY1**	2.0596657	0E+00	F8: Fascia-like myofibroblast
**LOXL2**	1.716715	0E+00	F8: Fascia-like myofibroblast
**PGM2L1**	2.620942	0E+00	F8: Fascia-like myofibroblast
**FNDC1**	2.2222972	0E+00	F8: Fascia-like myofibroblast
**MYL9**	1.4720716	0E+00	F8: Fascia-like myofibroblast
**ANGPTL2**	1.8625422	0E+00	F8: Fascia-like myofibroblast
**TMED9**	1.4776655	0E+00	F8: Fascia-like myofibroblast
**EVI2A**	3.837005	0E+00	F8: Fascia-like myofibroblast
**SYCE1L**	2.8354263	0E+00	F8: Fascia-like myofibroblast
**ITGA10**	3.3948631	0E+00	F8: Fascia-like myofibroblast
**AKAP13**	1.4193492	0E+00	F8: Fascia-like myofibroblast
**GOLIM4**	1.6330067	0E+00	F8: Fascia-like myofibroblast
**PFN1**	1.2055415	0E+00	F8: Fascia-like myofibroblast
**ACTN1**	1.490603	0E+00	F8: Fascia-like myofibroblast
**CTSB**	1.3845613	0E+00	F8: Fascia-like myofibroblast
**PTPRD**	2.4800363	0E+00	F8: Fascia-like myofibroblast
**LSP1**	1.9702451	0E+00	F8: Fascia-like myofibroblast
**UBE2E1**	1.6646061	0E+00	F8: Fascia-like myofibroblast
**CD70**	3.7735188	0E+00	F8: Fascia-like myofibroblast
**KCNMA1**	2.710709	0E+00	F8: Fascia-like myofibroblast
**RPLP2**	0.77414787	0E+00	F8: Fascia-like myofibroblast
**PSD3**	1.5101352	0E+00	F8: Fascia-like myofibroblast
**RABAC1**	1.075763	0E+00	F8: Fascia-like myofibroblast
**RLF**	1.7547435	0E+00	F8: Fascia-like myofibroblast
**COMP**	4.210525	0E+00	F_Fascia
**THBS4**	4.40946	0E+00	F_Fascia
**SFRP4**	3.40453	0E+00	F_Fascia
**LHFPL6**	2.1232116	0E+00	F_Fascia
**MALAT1**	1.4320302	0E+00	F_Fascia
**CD9**	2.1785376	0E+00	F_Fascia
**DKK 3.00**	2.2870576	9.2514E-296	F_Fascia
**S100A4**	1.3086138	5.85341E-273	F_Fascia
**CADM1**	2.176897	5.30988E-269	F_Fascia
**PRELP**	2.887861	6.34642E-254	F_Fascia
**CDH13**	2.5596805	5.14599E-245	F_Fascia
**AQP1**	2.337244	4.81833E-232	F_Fascia
**RPL13A**	1.1519212	1.31427E-228	F_Fascia
**IGFBP6**	3.0464706	4.58461E-223	F_Fascia
**MGP**	1.5463631	3.91201E-222	F_Fascia
**ADIRF**	1.6110848	4.83297E-221	F_Fascia
**P4HA3**	2.2837029	3.5404E-212	F_Fascia
**C1QTNF3**	2.0839648	1.62222E-209	F_Fascia
**ASPN**	2.6145942	3.80886E-202	F_Fascia
**CAMK1D**	1.7553657	1.80116E-189	F_Fascia
**MFGE8**	1.87796	1.41778E-186	F_Fascia
**CCN3**	3.5656614	2.51623E-176	F_Fascia
**OAF**	2.4430404	1.25255E-174	F_Fascia
**FNDC1**	2.085148	4.04652E-172	F_Fascia
**RPS17**	1.1515987	7.89024E-170	F_Fascia
**CILP2**	3.7344131	1.60466E-167	F_Fascia
**FMOD**	1.6823578	6.69676E-165	F_Fascia
**SPON2**	1.3979038	1.6335E-165	F_Fascia

**RPS20**	1.0986509	3.46266E-164	F_Fascia
**COL14A1**	1.3307773	4.01291E-160	F_Fascia
**FN1**	1.2343749	9.44314E-159	F_Fascia
**ACTB**	0.9640067	1.86057E-155	F_Fascia
**AEBP1**	1.1160355	2.48829E-151	F_Fascia
**PDGFRL**	1.6489707	4.77996E-149	F_Fascia
**TM4SF1**	3.8058374	2.14928E-148	F_Fascia
**FOXP1**	1.3789443	4.57002E-139	F_Fascia
**COL12A1**	1.3222148	1.57157E-137	F_Fascia
**GABARAPL2**	1.3936877	1.37817E-136	F_Fascia
**SOX5**	3.2607954	3.51614E-135	F_Fascia
**ACAN**	2.1666877	1.08143E-134	F_Fascia
**PSD3**	1.4335655	2.75442E-134	F_Fascia
**KCNMA1**	2.2064831	1.01012E-133	F_Fascia
**SEMA3C**	1.8370051	2.84143E-129	F_Fascia
**ANGPTL2**	1.6013712	4.54226E-129	F_Fascia
**SCRG1**	4.4375377	9.69664E-128	F_Fascia
**RPL31**	0.93275666	3.76181E-127	F_Fascia
**HIP1**	1.7547174	1.46569E-126	F_Fascia
**TGFBI**	1.3531042	1.92866E-126	F_Fascia
**CRIP1**	1.2102804	2.99965E-126	F_Fascia
**NDFIP1**	1.2824308	1.03616E-125	F_Fascia
**NBL1**	1.1857702	4.84874E-123	F_Fascia
**BMPR1B**	2.7216666	5.10726E-120	F_Fascia
**CLU**	1.9845396	6.31332E-120	F_Fascia
**PRG4**	4.7547364	2.83543E-118	F_Fascia
**PDLIM3**	1.6149781	9.17E-115	F_Fascia
**PIEZO2**	2.0392423	4.48394E-112	F_Fascia
**CRISPLD1**	2.1913798	1.08762E-111	F_Fascia
**IGF1**	1.823736	7.11699E-109	F_Fascia
**PTGIS**	1.9920648	8.20805E-107	F_Fascia
**MATN2**	2.3173106	1.09819E-106	F_Fascia
**DGKI**	1.85427	3.96071E-105	F_Fascia
**NIBAN1**	1.6868916	9.25474E-105	F_Fascia
**PLPP1**	1.1459891	9.55363E-105	F_Fascia
**LUM**	1.009897	1.2119E-104	F_Fascia
**MT1X**	1.2483386	3.27123E-101	F_Fascia
**PDLIM5**	1.4886847	2.07686E-97	F_Fascia
**RPL7**	0.83851016	2.799E-97	F_Fascia
**CCN5**	1.6571542	1.09651E-96	F_Fascia
**ENPP1**	2.4653409	1.80472E-95	F_Fascia
**MMP2**	0.8247049	1.34497E-95	F_Fascia
**CCN2**	1.587395	2.53289E-95	F_Fascia
**PTN**	1.4422106	8.05698E-95	F_Fascia
**RPL27A**	0.7721324	5.31254E-94	F_Fascia
**LIMA1**	1.0979769	3.84734E-92	F_Fascia
**CRTAC1**	4.6007614	4.12027E-91	F_Fascia
**SULF1**	1.7308729	4.5067E-91	F_Fascia
**MTSS1**	2.0351002	4.97643E-91	F_Fascia
**DPP4**	1.6044074	4.77403E-89	F_Fascia
**ANGPTL7**	7.8115973	1.26678E-88	F_Fascia
**CAPG**	1.3091903	1.05314E-88	F_Fascia
**CCPG1**	1.2548851	1.15396E-88	F_Fascia

**EZR**	2.2224233	1.64055E-88	F_Fascia
**THBS3**	1.6330616	2.13615E-88	F_Fascia
**GALNT15**	2.3800054	3.99904E-87	F_Fascia
**FXYD6**	1.7433507	1.55697E-86	F_Fascia
**EDIL3**	1.1539118	1.89576E-86	F_Fascia
**RBP4**	2.9855456	2.02794E-84	F_Fascia
**ADK**	1.2636675	1.63351E-84	F_Fascia
**DLC1**	1.1641797	1.42598E-83	F_Fascia
**ANKH**	1.6323258	2.65514E-83	F_Fascia
**RETREG1**	1.4935449	4.76988E-83	F_Fascia
**UACA**	1.4934962	4.87864E-83	F_Fascia
**CPXM2**	2.2270007	7.33126E-83	F_Fascia
**CNN3**	0.86480355	9.16399E-82	F_Fascia
**PPP3CA**	1.1560755	2.61906E-81	F_Fascia
**GALNT18**	2.6094947	1.88887E-80	F_Fascia
**ACTG1**	0.665369	2.58931E-80	F_Fascia
**CDO1**	2.9316754	7.2733E-80	F_Fascia
**ELMO1**	2.0210888	3.49995E-77	F_Fascia
**COL8A1**	1.4285455	8.53552E-77	F_Fascia

Supplementary Note 1

Fibroblast subtypes were observed across different datasets consistently (Extended Data Fig. 1b), with the exception of a dataset by Ji et al., which used a dissociation protocol containing trypsin.

To attempt to disentangle regional site effects from other covariates, we used CellDISECT - a novel method for disentangled representations in multi-batch, multi-covariate single-cell data analysis [113]. We used anatomic site, dataset ID, and fibroblast subtype as covariate inputs and interrogated the anatomical site latent space, coloured by cell type and anatomic site (Extended Data Fig. 1f). Across locations, *F5: Schwann-like* fibroblasts appeared more common on acral sites. *F1: Superficial* appeared less common on the face and acral sites. Face samples were predominantly from advanced age skin (collection related to basal cell carcinoma excision), and thus the decreased *F1: Superficial* could be associated with age, particularly given the association of age with thinner epidermis and the proposed role of *F1: Superficial* in epithelial patterning. Distinct locations formed distinct clusters without overlap. Distinct gene expression by anatomical region was observed, including HOX genes (Extended Data Fig. 1g). *HOXA13* was expressed in acral, arm, and hip sites, which may reflect imperfect reporting of anatomic locations as *HOXA13* is associated with acral and genital sites[114]. A further analysis of anatomic location using samples with minimal biological and technical differences will be valuable in future studies.

Supplementary Note 2

We performed an additional integration using the same integration strategy but including fascial fibroblasts, Schwann cells, and pericytes to ensure that fibroblast clusters showed distinct gene expression to these. We also included fascial fibroblasts to understand their

relationship to *F2: Universal*. We included fibroblasts obtained from human non-lesional palmar fascia (specific hand anatomical site with minimal fat).

While *Universal PI16+* fibroblasts are postulated to represent a precursor fibroblast cell state[50], skin reticular fibroblasts are not recognised as precursors in skin. Instead, fascial fibroblasts, found deep beneath the fat layer of the skin and thus not typically captured from skin biopsies, have been reported as potential progenitor cells in mouse skin[53]. *F_Fascia* formed a subset of *F2: Universal* (Extended Data Fig. 1i), expressing *F2: Universal* marker genes such as *CD34*, *PI16*, *DPP4*, and *LGR5*. *F_Fascia* also showed very high expression of marker genes for *F2/3: Perivascular,* namely *MYOC* and *GDF10*. *F_Fascia* additionally expressed unique marker genes that were not expressed by *F2: Universal* and *F2/3: Perivascular,* including *ITGA10*, *THBS4*, and *SCX*. Overall, these results suggest that *F2: Universal* in skin and fascial fibroblasts are transcriptomically similar and may have a progenitor role. Further work is needed to distinguish site-related genes from fascia-related genes as all fascia fibroblasts were obtained from the hand.

We also sought to distinguish fibroblast subtypes from Schwann cells and pericytes as *F5* fibroblasts were Schwann-like and *F2/3* and *F3* fibroblast subtypes were enriched in perivascular regions. *F5: Schwann-like* fibroblasts and Schwann cells formed separate clusters that were distinguished by the differential expression of fibroblast markers (*PDGFRA*, *LUM*) in *F5: Schwann-like fibroblasts* and *SOX10*/*S100B* in Schwann cells (Extended Data Fig. 1k,j). Pericytes were distinguished from fibroblasts by *RGS5* expression (Extended Data Fig. 1k).

Supplementary Note 3

Given the identification of *F3* and *F6* fibroblasts in IBD, we performed a joint integration of our skin data with IBD with annotated cells from the original publication from Elmentaite et al.[78] to both 1) confirm cell similarity for *F3* and *F6* subtypes and 2) utilise insights gained from the original study on cell identity to confirm appropriate labelling of these populations in our work.

In this integration, we identified similar clustering for three populations (Extended Data Fig. 8g). First, *F3: FRC-like* fibroblasts were equivalent to intestinal "*T reticular cells*". T zone reticular cells are a subtype of FRC, suggesting our results were correct to predict FRC-like cells in IBD. Second, *F6: Inflammatory myofibroblasts* were equivalent to intestinal "*Stroma (MMP1+)*". Fibroblasts in other IBD studies with similar gene expression to *Stroma (MMP1+)* are termed inflammatory myofibroblasts[80], suggesting correct attribution of this fibroblast phenotype. Finally, we observed that skin *F2/3: Perivascular* fibroblasts were similar to several stromal populations (*Stromal 3 (C7+)*, *mLN Stroma (FMO2+)*, *Transitional Stromal 3 (C3+)*) (Extended Data Fig. 8g). mLN Stroma cells were suggested to possibly represent an adipose stromal cell population in the original study[78], pointing towards a pre-adipocyte enriched population in different human tissues.

For lung, we assessed gene expression of reported lung fibroblast subtypes from the Human Lung Cell Atlas (HLCA)[8] (Extended Data Fig. 8f). Adventitial and subpleural fibroblasts expressed *F2: Universal* markers (*CD34*, *PI16*, *MFAP5*, *DPP4*, *CD70*). *Universal* fibroblasts are also known as adventitial fibroblasts[50]. *F2/3: Perivascular* fibroblast marker genes were expressed by *Lipofibroblasts*, consistent with the pre-adipocyte enrichment we identified in skin (Extended Data Fig. 8f).

In lung, *CCL19* expression is implicated in idiopathic pulmonary fibrosis (IPF) and COVID19 pathogenesis, recruiting dendritic cell populations to fibrotic foci and mediating the formation

of ectopic lymphoid structures, respectively[115,116117]. We observed *F3: FRC-like* cells were observed in interstitial lung disease, pulmonary fibrosis, and COVID-19 (Fig. 7a). However, we did not identify a clear *FRC-like* subtype in lung (Extended Data Fig. 8f). We re-clustered HLCA fibroblasts and observed high expression of *F3: FRC-like* marker genes in one cluster that was predominantly formed of *Adventitial* fibroblasts (Extended Data Fig. 8e), but also fibroblasts with no label, suggesting the possibility of an *F3: FRC-like* subtype in human lung.

We also identified *Nerve-associated fibroblast* in lung that expressed *F5: NGFR+* (Schwann-like) fibroblast markers (Extended Data Fig. 8f). Lung *Activated myofibroblasts* expressed select skin myofibroblast markers (*CTHRC1*, *SFRP4*, *ASPN*, *ACTA2*, *OGN*, *ADAM12*, *FABP5*, *LRRC17*).

Not all genes could be assessed as the cross-tissue dataset contained ~17 000 genes due to a limited number of genes in the endometrial dataset.

Supplementary Note 4

We identified major differences between adult skin and prenatal skin fibroblasts (Fig.8a-b), with many genes uniquely expressed by adult and prenatal skin fibroblasts (Extended Data Fig. 10b). This concurs with prior work in mice of major shifts between prenatal and postnatal fibroblast populations[118119]. However, we hypothesised that despite global differences, transcriptomic similarity would be evident with adult fibroblast subtypes.

Using our defined marker genes for adult fibroblast subtypes, we observed select shared gene expression with prenatal skin populations (Extended Data Fig. 10c). For example, prenatal *HOXC5+* and *Pre-dermal condensate* fibroblasts showed transcriptomic similarity to *F1: Superficial* fibroblasts in adult skin (*APCDD1*, *WIF1*, *RSPO1*) (Extended Data Fig. 10c).

Interestingly, prenatal *HOXC5+* and *Pre-dermal condensate* fibroblasts were reported to give rise to hair follicle fibroblast populations[66]. A transcriptomic similarity with *F1: Superficial* would thus be consistent with a “regenerative”/scarless profile for *F1: Superficial* fibroblasts in adult skin.

Prenatal *WNT2+* fibroblasts, which have a deep location in prenatal skin[66], showed the highest expression of *F2: Universal* markers (*CD34*, *PI16*, *MFAP5*, *LGR5*) (Extended Data Fig. 10c). A deep pro-scarring population is reported in prenatal mouse skin[55,120], and postnatal skin fibroblasts wounds have been observed to reactivate prenatal gene expression during scar formation[120]. Consistent with this finding, we observed that *WNT2+* fibroblasts populations in human prenatal skin expressed select gene markers of disease-specific adult skin myofibroblast populations, including *WNT2*, *ASPN*, *CDH2*, and *SFRP4* (Extended Data Fig. 10c).

For other populations, we observed that prenatal *PEAR1+* fibroblasts, which have a perivascular location in prenatal skin, also expressed *Universal* markers as well as *F2/F3* markers (*CXCL12*, *APOE*, *PPARG*, *APOC1*), and thus may represent progenitor to *F2/3: Perivascular*. We additionally showed these similarities in an unbiased way, calculating gene module scores using both the top 50 and top 1000 DEGs for each adult skin fibroblast population (Fig. 8b and Extended Data Fig. 10d).

Supplementary Note only references

113. Shamsaie, K., Megas, S., Asadollahzadeh, H., Teichmann, S. A. & Lotfollahi, M. Disentangling Covariates to Predict Counterfactuals for single-cell data. *https://openreview.net › forumhttps://openreview.net › forum* (2023).

114. Rinn, J. L. *et al.* A dermal HOX transcriptional program regulates site-specific epidermal fate. *Genes Dev.* **22**, 303–307 (2008).

115. Luther, S. A. *et al.* Differing activities of homeostatic chemokines CCL19, CCL21, and CXCL12 in lymphocyte and dendritic cell recruitment and lymphoid neogenesis. *J. Immunol.* **169**, 424–433 (2002).

116. Russo, R. C., Quesniaux, V. F. J. & Ryffel, B. Homeostatic chemokines as putative therapeutic targets in idiopathic pulmonary fibrosis. *Trends Immunol.* **44**, 1014–1030 (2023).

117. Mothes, R. *et al.* Distinct tissue niches direct lung immunopathology via CCL18 and CCL21 in severe COVID-19. *Nat. Commun.* **14**, 791 (2023).

118. Qiu, C. *et al.* A single-cell time-lapse of mouse prenatal development from gastrula to birth. *Nature* **626**, 1084–1093 (2024).

119. Rognoni, E. *et al.* Fibroblast state switching orchestrates dermal maturation and wound healing. *Mol. Syst. Biol.* **14**, e8174 (2018).

120. Rinkevich, Y. *et al.* Skin fibrosis. Identification and isolation of a dermal lineage with intrinsic fibrogenic potential. *Science* **348**, aaa2151 (2015).
