# Supplementary material for: A single-cell and spatial genomics atlas of human skin fibroblasts reveals shared disease-related fibroblast subtypes across tissues
Source: Nat Immunol. 2025 Sep 24;26(10):1807–20. doi: 10.1038/s41590-025-02267-8 (PMC12479362; doi:10.1038/s41590-025-02267-8)
Supplement: Supplementary file 2 — Reporting Summary [file 41590_2025_2267_MOESM2_ESM.pdf]

nature portfolio

Corresponding author(s): Muzlifah Haniffa

Last updated by author(s): 17/07/25

Reporting Summary

Nature Portfolio wishes to improve the reproducibility of the work that we publish. This form provides structure for consistency and transparency in reporting. For further information on Nature Portfolio policies, see our Editorial Policies and the Editorial Policy Checklist.

Statistics

For all statistical analyses, confirm that the following items are present in the figure legend, table legend, main text, or Methods section.

n/a	Confirmed	
☐	☒	The exact sample size (*n*) for each experimental group/condition, given as a discrete number and unit of measurement
☐	☒	A statement on whether measurements were taken from distinct samples or whether the same sample was measured repeatedly
☒	☐	The statistical test(s) used AND whether they are one- or two-sided<br>*Only common tests should be described solely by name; describe more complex techniques in the Methods section.*
☐	☒	A description of all covariates tested
☐	☒	A description of any assumptions or corrections, such as tests of normality and adjustment for multiple comparisons
☐	☒	A full description of the statistical parameters including central tendency (e.g. means) or other basic estimates (e.g. regression coefficient) AND variation (e.g. standard deviation) or associated estimates of uncertainty (e.g. confidence intervals)
☐	☒	For null hypothesis testing, the test statistic (e.g. *F*, *t*, *r*) with confidence intervals, effect sizes, degrees of freedom and *P* value noted<br>*Give P values as exact values whenever suitable.*
☒	☐	For Bayesian analysis, information on the choice of priors and Markov chain Monte Carlo settings
☒	☐	For hierarchical and complex designs, identification of the appropriate level for tests and full reporting of outcomes
☒	☐	Estimates of effect sizes (e.g. Cohen's *d*, Pearson's *r*), indicating how they were calculated

Our web collection on statistics for biologists contains articles on many of the points above.

Software and code

Policy information about availability of computer code

Data collection	https://github.com/cellgeni/reprocess_public_10x was used to reprocess scRNA datasets. Software used for data alignment, quantification, quality control and downsteam analysis of<br>single cell datasets in this study are described in detail in manuscript 'Methods'.
Data analysis	Single-cell sequencing data were processed and analysed using publicly available software packages. Further information on analytical approaches used in the study are further detailed in manuscript 'Methods' section.<br>Software and packages versions used for computational analysis are as follows:<br>STARsolo (version v2.7.10a_alpha_220818); cellbender (version 0.3); Spaceranger (version 1.3.0); Scanpy (version 1.8.1 and 1.9.3); anndata (version 0.8); scCODA (version 0.1.9), Scrublet (version 0.2.3); pandas (version 2.2.1), anndata (version 0.10.6), scanpy (version 1.9.8), numpy (version 1.24.0), scrublet (version 0.2.3); CellPhoneDB (version 5) , Monocle 3 (v 1.3.7),decoupler (version 1.6.0),squidpy (version 1.4.1), scarches including scpoli (version 0.6.1); scipy (version 1.13.1); scvi (version 1.1.2) cell2location (version 0.1.3), Cellrank (version 2); CellDISECT (0.2.0b1.)<br><br>Code Availability:<br>Single-cell sequencing data were processed and analysed using publicly available software packages. The code generated during this study is available at Github: https://github.com/haniffalab/skin_fibroblast_atlas

For manuscripts utilizing custom algorithms or software that are central to the research but not yet described in published literature, software must be made available to editors and reviewers. We strongly encourage code deposition in a community repository (e.g. GitHub). See the Nature Portfolio guidelines for submitting code & software for further information.

Data

Policy information about availability of data

All manuscripts must include a data availability statement. This statement should provide the following information, where applicable:

- Accession codes, unique identifiers, or web links for publicly available datasets
- A description of any restrictions on data availability
- For clinical datasets or third party data, please ensure that the statement adheres to our policy

Newly-generated sequencing data (Visium) for inflamed atopic dermatitis from this study is available from EGA: EGAS00001006482 (sample EGAN00004379723). Healthy Visium data is available from Array Express : E-MTAB-15458. Xenium data is available from https://www.ebi.ac.uk/biostudies/bioimages/studies/S-BIAD2214. Our processed data can be downloaded and explored on an online webportal: https://collections.cellatlas.io/skin-fibroblast. Publicly-available scRNA-seq data and access links are shown in Supplementary Table 1.

Research involving human participants, their data, or biological material

Policy information about studies with human participants or human data. See also policy information about sex, gender (identity/presentation), and sexual orientation and race, ethnicity and racism.

Reporting on sex and gender	Extended Data Fig.1
Reporting on race, ethnicity, or other socially relevant groupings	N/A
Population characteristics	Extended Data Fig 1.
Recruitment	Visium data: All research ethics committee and regulatory approval were in place for the collection of research samples at Newcastle and for their storage at the Newcastle Dermatology Biobank (REC reference number: 19/NE/0004). Adult healthy skin was sampled from normally discarded surplus skin from skin surgery during defect reconstructions. Patients were provided with a patient information leaflet (PIL) relevant to donating normally discarded surplus skin for research and an associated consent form was reviewed and signed by the patient. Patients with atopic dermatitis were also recruited for this study who had not had systemic treatment (such as methotrexate) for at least 4 weeks prior to donating skin and no topical steroids to the biopsy site for at least one week before the biopsy was taken. Patients were provided with a PIL relevant to donating skin for use in research and an associated consent form was reviewed and signed by the patient prior to recruitment<br><br>Xenium data: All research ethics committees and regulatory approvals were in place for the collection and storage of research samples at St John's Institute of Dermatology, Guy's Hospital, London (REC reference number: EC00/128). Patients were provided with a PIL relevant to donating skin for use in research and an associated consent form was reviewed and signed by the patient prior to recruitment
Ethics oversight	Visium: All research ethics committee and regulatory approval were in place for the collection of research samples at Newcastle and for their storage at the Newcastle Dermatology Biobank (REC reference number: 19/NE/0004).<br><br>Xenium: All research ethics committees and regulatory approvals were in place for the collection and storage of research samples at St John's Institute of Dermatology, Guy's Hospital, London (REC reference number: EC00/128).<br><br>Immunofluorescence: All research ethics committees and regulatory approvals were in place for the collection and storage of Atopic Dermatitis skin samples at St John's Institute of Dermatology, Guy's Hospital, London (REC reference number: EC00/128) and Hidradenitis Suppurativa skin samples at Newcastle Dermatology Biobank (REC reference number: 19/NE/0004).

Note that full information on the approval of the study protocol must also be provided in the manuscript.

Field-specific reporting

Please select the one below that is the best fit for your research. If you are not sure, read the appropriate sections before making your selection.

☒ Life sciences ☐ Behavioural & social sciences ☐ Ecological, evolutionary & environmental sciences

For a reference copy of the document with all sections, see nature.com/documents/nr-reporting-summary-flat.pdf

Life sciences study design

All studies must disclose on these points even when the disclosure is negative.

Sample size	As per the Human Cell Atlas white paper (https://www.humancellatlas.org/wp-content/uploads/2019/11/HCA_WhitePaper_18Oct2017-

Sample size	copyright.pdf), sample size was determined by recent experience using these technologies in relevant tissues. The sample size was made as large as it could be possible based on the availability of suitable materials.
Data exclusions	In this study, data exclusions were limited to removal of low quality single cells of downstream analysis of 'good quality' cells in scRNA-seq datasets. Any such data filtering steps are detailed in full in manuscript 'Methods'.
Replication	We included scRNA-seq data from 32 datasets and 251 samples. For healthy spatial transcriptomic data we included 2 healthy donors (Visium), 2 non-lesional atopic dermatitis donors (Xenium), and 2 lesional atopic dermatitis donors (1 Visium, 1 Xenium).
Randomization	Randomization was not applicable in this study, because we describe an exploratory analysis in a discovery cohort.
Blinding	Blinding for scRNA-seq data generation was not necessary as data were analyzed together using unbiased clustering and annotation of clusters. Analyses with sample characteristics as a variable were not performed until after data were annotated. For other experiments, blinding is not applicable, because we did not perform a clinical study with specific clinical questions.

Reporting for specific materials, systems and methods

We require information from authors about some types of materials, experimental systems and methods used in many studies. Here, indicate whether each material, system or method listed is relevant to your study. If you are not sure if a list item applies to your research, read the appropriate section before selecting a response.

Materials & experimental systems

n/a	Involved in the study
☐	☒ Antibodies
☒	☐ Eukaryotic cell lines
☒	☐ Palaeontology and archaeology
☒	☐ Animals and other organisms
☒	☐ Clinical data
☒	☐ Dual use research of concern
☒	☐ Plants

Methods

n/a	Involved in the study
☒	☐ ChIP-seq
☒	☐ Flow cytometry
☒	☐ MRI-based neuroimaging

Antibodies

Antibodies used	Fresh frozen OCT-embedded skin samples were sectioned at 10 µm thickness directly onto the 10X Genomics xenium slide kept at -20°C. We then ran the slides through the 10x Genomics Xenium prime in situ gene expression protocol and imaged using the 10X Genomics Xenium Analyzer.
Validation	n/a

Plants

Seed stocks	*Report on the source of all seed stocks or other plant material used. If applicable, state the seed stock centre and catalogue number. If plant specimens were collected from the field, describe the collection location, date and sampling procedures.*
Novel plant genotypes	*Describe the methods by which all novel plant genotypes were produced. This includes those generated by transgenic approaches, gene editing, chemical/radiation-based mutagenesis and hybridization. For transgenic lines, describe the transformation method, the number of independent lines analyzed and the generation upon which experiments were performed. For gene-edited lines, describe the editor used, the endogenous sequence targeted for editing, the targeting guide RNA sequence (if applicable) and how the editor was applied.*
Authentication	*Describe any authentication procedures for each seed stock used or novel genotype generated. Describe any experiments used to assess the effect of a mutation and, where applicable, how potential secondary effects (e.g. second site T-DNA insertions, mosiacism, off-target gene editing) were examined.*
